# Macrophages Drive Ferroptosis Resistance after Radiotherapy

**DOI:** 10.34133/cancomm.0039

**Published:** 2026-09-04

**Authors:** Hyewon Chung, Sang Wha Kim, Jae Won Oh, Gyu Mi Park, Yurim Cho, Hae Suk Choi, MinJi Kim, Kwang Pyo Kim, Yi Rang Na, Hye Seung Lee, Hak Jae Kim, Seung Hyeok Seok

**Affiliations:** ^1^ Department of Microbiology and Immunology, Seoul National University College of Medicine, Seoul, South Korea.; ^2^ Institute of Endemic Diseases, Seoul National University Medical Research Center, Seoul, South Korea.; ^3^ College of Veterinary Medicine and Institute of Veterinary Science, Kangwon National University, Chuncheon, Gangwon, South Korea.; ^4^ Department of Applied Chemistry, Institute of Natural Science, Global Center for Pharmaceutical Ingredient Materials, Kyung Hee University, Yongin, South Korea.; ^5^ Department of Biomedical Sciences, Seoul National University College of Medicine, Seoul, South Korea.; ^6^ Cancer Research Institute, Seoul National University, Seoul, South Korea.; ^7^ Department of Biomedical Science and Technology, Kyung Hee Medical Science Research Institute, Kyung Hee University, Seoul, South Korea.; ^8^ Translational Immunology Lab, Department of Transdisciplinary Medicine, Seoul National University Hospital, Seoul, South Korea.; ^9^ Immunology Core Facility, Advanced Omics Research Core, Biomedical Research Institute, Seoul National University Hospital, Seoul, South Korea.; ^10^ Department of Pathology, Seoul National University Hospital, Seoul National University College of Medicine, Seoul, South Korea.; ^11^ Department of Radiation Oncology, Seoul National University College of Medicine and Hospital, Seoul, South Korea.

## Abstract

**Background:** Radiotherapy eliminates most tumor cells but spares persister tumor cells that evade cell death and drive relapse. Increasing evidence suggests that stromal components of the tumor microenvironment influence treatment responses, yet whether macrophages actively reprogram tumor-intrinsic stress responses to promote radioresistance remains unclear. Here, we investigated the mechanisms by which macrophage–tumor cell interactions regulate ferroptosis and tumor survival after irradiation. **Methods:** We used macrophage–tumor cell coculture systems, Transwell separation assays, and 3D microfluidic models to examine contact-dependent effects on tumor survival following irradiation. Kinome-wide small interfering RNA screening, RNA sequencing, lipidomic profiling, and quantitative proteomic analysis of secretomes were performed to identify signaling pathways and metabolic changes. Genetic and pharmacological perturbation of Ephrin receptor b4 (Ephb4) signaling were evaluated in vitro and in syngeneic mouse tumor models. Clinical relevance was assessed using transcriptomic analyses and immunohistochemical staining of patient tumor specimens. **Results:** Macrophage contact reduced lipid peroxidation and cell death in irradiated tumor cells in a contact-dependent manner. Kinome screening identified Ephb4 as a key mediator induced by irradiation in tumor cells. Ephb4 engagement with ephrinb2 on macrophages initiated bidirectional signaling that increased expression of ferroptosis-protective genes (solute carrier family 7 member *11* [*Slc7a11*], solute carrier family 3 member 2 [*Slc3a2*], and glutathione peroxidase 4 [*Gpx4*]) in tumor cells while activating the toll-like receptor 2- nuclear factor-kappa B pathway and interleukin-6 (IL-6) production in macrophages. Macrophage-derived IL-6 further sustained ferroptosis resistance in tumor cells, and Ephb4-driven secretion of cathepsin S amplified macrophage IL-6 production through a feedforward loop. Genetic or pharmacological inhibition of Ephb4 restored lipid peroxidation and markedly enhanced radiosensitivity in vitro and in vivo. Analysis of patient datasets demonstrated increased EPHB4 expression following radiotherapy and an association between high EPHB4 expression, reduced ferroptosis signatures, and poor treatment response. **Conclusions:** These findings identify a macrophage-driven ferroptosis evasion program that enables tumor cell survival after irradiation and demonstrate that Ephb4 coordinates bidirectional tumor–macrophage signaling to sustain this resistance. Targeting the Ephb4–ephrinb2 axis represents a potential strategy to enhance ferroptosis and improve radiotherapy efficacy in resistant tumors.

## Background

Radiotherapy (RT) is a primary treatment modality administered with curative or palliative intent in over half of patients with cancer [1–3]. Although effective in achieving local tumor control, the high doses required for complete tumor eradication are constrained by unavoidable collateral damage to adjacent normal tissues. To mitigate such toxicity, RT is commonly delivered in fractionated doses over multiple sessions [4–6]. While this approach facilitates normal tissue repair, it also permits a subset of tumor cells to persist and adapt during treatment-free intervals [7–9]. These surviving populations, termed persistent cancer cells, exhibit phenotypic plasticity and stress tolerance and are regarded as precursors of therapy-resistant clones that drive tumor relapse [7,10,11]. Understanding how persistent cancer cells evade RT-induced cytotoxicity during this early adaptive window is crucial for identifying actionable targets to prevent the emergence of durable radioresistance.

One of the key adaptations that enables residual tumor cells to resist RT involves dynamic remodeling of the tumor microenvironment (TME) [12,13]. Beyond its direct DNA-damaging effects on tumor cells, RT induces profound immunologic perturbations, including vascular disruption, cytokine release, and immunogenic cell death [13,14]. These changes can activate host antitumor immunity, providing a rationale for combining RT with immune checkpoint blockade [15,16]. However, these immunogenic effects are often counteracted by the concurrent recruitment of immunosuppressive myeloid populations, particularly tumor-associated macrophages (TAMs), which dampen antitumor responses and promote tumor regrowth [17–21]. Recent studies have begun to elucidate how RT programs macrophage phenotypes in the TME. For example, tumor-derived amphiregulin, up-regulated upon irradiation (IR), drives monocyte differentiation into immunosuppressive TAMs [22]. Additionally, stromal fibroblast growth factor signaling [23] and macrophage-intrinsic signal transducer and activator of transcription 3 activation [24] foster tumor-supportive macrophage phenotypes. These findings highlight TAMs as key regulators of the post-IR immunosuppressive niche.

A critical unresolved question, however, is whether macrophages also actively reprogram tumor-intrinsic stress responses to confer radioresistance. Notably, spatial and single-cell transcriptomic analyses of esophageal squamous cell carcinoma (ESCA) patient tissues revealed that TAMs infiltrate more deeply into the tumor core following RT [18]. This spatial rearrangement positions them in close proximity to residual tumor cells, raising the possibility of direct macrophage–tumor cell crosstalk that could affect tumor fate after IR. A well-characterized mechanism involves the up-regulation of the CD47–signal regulatory protein alpha axis in irradiated tumor cells, which helps them escape phagocytic clearance by macrophages [22,25,26]. While such immune evasion may allow the persistence of damaged tumor cells, passive survival alone cannot fully explain how these surviving cells adapt to withstand subsequent radiation exposure. Of particular interest, macrophages are capable of engaging in juxtacrine signaling through physical contact, a mode of communication implicated in sustaining tumor cell survival and stemness [27,28]. These observations led us to hypothesize that RT-induced spatial juxtaposition between TAMs and tumor cells triggers a contact-driven signaling cascade that suppresses cell death pathway activation, thereby establishing a protective niche for the emergence of therapy-resistant clones.

In this study, we uncovered a critical mechanism by which macrophages and tumor cells cooperate to promote ferroptosis resistance and tumor regrowth after RT. Using a kinome-wide small interfering RNA (siRNA) screen in a macrophage–tumor coculture model, we identified Ephrin receptor b4 (Ephb4) as a key mediator up-regulated in irradiated tumor cells. Upon physical contact with ephrinb2-expressing macrophages, Ephb4 initiates bidirectional signaling that coordinates both tumor-intrinsic antioxidant defenses and supportive paracrine cues from macrophages. These interactions form a feedback loop that shields irradiated tumor cells from ferroptotic death. Together, our findings elucidate a previously unrecognized ferroptosis-protective circuit mediated by macrophage–tumor cell contact and nominate the Ephb4–ephrinb2 axis as a therapeutic target to disrupt this niche and enhance radiosensitivity.

## Materials and Methods

### Mice

Female wild-type (WT) C57BL/6 and BALB/c mice (aged 8 to 10 weeks) were purchased from Orient Bio (Korea). Toll-like receptor 2 (*Tlr2*)-deficient mice were kindly provided by Professor Jong-Hwan Park. All mice were housed in a specific pathogen-free facility at Seoul National University College of Medicine under controlled conditions (23°C, 55% humidity, 12-h light/dark cycle) with free access to food and water. All animal experiments were conducted in accordance with protocols approved by the Institutional Animal Care and Use Committee (IACUC) of Seoul National University (IACUC No. SNU-210122-3-3).

### Cell lines and culture

Murine breast carcinoma 4T1, murine melanoma B16F10 cells, and human embryonic kidney 293T (HEK293T) cells were obtained from the American Type Culture Collection. Murine colorectal adenocarcinoma MC38 cells were purchased from Kerafast (Cat. No. ENH204-FP), and CT26 cells were acquired from the Korean Cell Line Bank (Cat. No. 80009). MC38 cells expressing green fluorescent protein (GFP) were kindly provided by Prof. Nam-Hyuk Cho. 4T1, and B16F10 cells were cultured in RPMI 1640 medium supplemented with 10% fetal bovine serum (FBS) and 1% penicillin–streptomycin (Gibco). MC38, MC38-GFP, CT26, and HEK293T cells were maintained in Dulbecco’s modified Eagle’s medium (DMEM) with 10% FBS and 1% penicillin–streptomycin. All cell lines were routinely tested for mycoplasma contamination.

### Human biospecimens

For patient sample analyses, the study cohort consisted of patients with stage II to III esophageal cancer who were considered eligible for surgical resection. All patients received neoadjuvant chemoradiotherapy followed by surgery at Seoul National University Hospital (Seoul, South Korea). The prescribed total radiation dose ranged from 41.4 to 44.0 Gy, delivered in 22 to 23 fractions using conventional fractionation. Surgical resection was performed 4 to 6 weeks after completion of RT, and tumor specimens used for analysis were obtained from post-RT surgical samples.

A total of 39 cases were included in the final analysis. Patients were selected according to the following criteria: (a) diagnosis of esophageal cancer at Seoul National University Hospital between February 2012 and August 2021; (b) receipt of neoadjuvant chemoradiotherapy followed by surgical resection; and (c) availability of both preoperative endoscopic biopsy specimens and corresponding surgical specimens. Among 102 patients who met these criteria, 39 cases with evaluable viable cancer cells remaining in the surgical specimens were ultimately included. This study was approved by the Institutional Review Board (IRB) of Seoul National University Hospital (IRB No. H-2301-100-1396) and was conducted in accordance with the Declaration of Helsinki. Written informed consent was obtained from all participants.

Tissue samples obtained from endoscopic biopsy or esophagectomy were fixed in 10% neutral buffered formalin for 6 to 72 h and embedded in paraffin. Hematoxylin and eosin staining was performed on 4-μm-thick sections. Tumor regression grade (TRG) was assessed in surgical resection specimens according to the modified Ryan scheme [29]: score 3 (no or poor response), score 2 (minimal response), score 1 (moderate response), and score 0 (complete response). For statistical analysis, patients with TRG scores of 2 or 3 were classified as nonresponders, whereas those with scores of 0 or 1 were classified as responders.

### Preparation of bone marrow-derived macrophages

Bone marrow-derived macrophages (BMDMs) were isolated from the femurs and tibias of 8- to 12-week-old C57BL/6 mice or *Tlr2*-deficient mice. Bone marrow cells were flushed and cultured in RPMI 1640 medium containing 10% FBS, 100 U/mL penicillin, 100 μg/mL streptomycin, and 2 mmol/L L-glutamine, supplemented with recombinant murine colony-stimulating factor 1 (50 ng/mL, Miltenyi Biotec). The medium was refreshed on days 3 and 5 with fresh colony-stimulating factor 1-containing medium. Fully differentiated macrophages were used after 7 d of culture.

### Generation of luciferase-expressing MC38 cells

Lentivirus was generated in HEK293T cells cultured in 24-well plates by cotransfecting the pMSCV/luciferase vector (300 ng), gag-pol vector (150 ng), and envelope vector (150 ng) using Lipofectamine 3000 (Invitrogen, Cat. No. L3000015) in Opti-MEM reduced-serum medium (Gibco, Cat. No. 31985070). Lentiviral supernatants were harvested at 50 h posttransfection and filtered through a 0.22-μm syringe filter (Sartorius, Cat. No. 16532-K) to remove cellular debris. Lentiviral supernatants were stored at −80°C until use. The filtered lentiviral supernatants were added to MC38 cells in the presence of polybrene (5 μg/mL; Sigma, Cat. No. H9268-5G). Stably transduced cells were selected with puromycin (4 μg/mL; Gibco, Cat. No. A11138-03). Subsequently, single-cell clones were isolated using a Sony SH800S cell sorter. Luciferase activity was verified by bioluminescence imaging following treatment with D-luciferin (PerkinElmer, Cat. No. 122799), and highly luminescent clones were expanded for downstream experiments.

### Bioluminescence imaging

To assess the growth of luciferase-labeled tumor cells in vitro, 2,000 cells per well were seeded in 96-well plates either alone or in coculture with BMDMs. After IR (6 Gy), luminescence was measured at the indicated time points for up to 8 d using an IVIS Spectrum imaging system (PerkinElmer). Total photon flux (photons/s) was quantified using Living Image software (PerkinElmer) and normalized to luminescence values from tumor cell-only wells for each corresponding time point.

### IR conditions and time point selection

IR doses were determined based on the experimental conditions. In 96-well plate-based assays, including bioluminescence imaging and kinome-wide siRNA screening, 6 Gy was used, as 10 Gy resulted in near-complete tumor cell loss and precluded reliable quantitative analysis. In contrast, a single dose of 10 Gy was applied in other in vitro and in vivo experiments to induce sufficient tumor cell damage.

Post-IR time points were determined based on cell viability under each condition. In tumor cell-only cultures, most cells were lost after day 4 following 10-Gy IR, and thus, day 4 was used as the latest time point for analysis. In tumor cell–macrophage coculture conditions, viable tumor cells persisted beyond this time point, allowing extended observation, with day 6 selected as a representative time point for analysis.

### Immunofluorescence staining

Frozen tissue sections or cultured cells were fixed in 4% paraformaldehyde for 15 min at room temperature and blocked in phosphate-buffered saline (PBS) containing 3% bovine serum albumin and 0.3% Triton X-100 (Sigma-Aldrich) for 1 h. Samples were then rinsed in staining buffer (1% bovine serum albumin and 0.3% Triton X-100 in PBS) and incubated overnight at 4°C with the following primary antibodies: anti-Ki67, anti-F4/80, anti-Pan-cytokeratin, anti-CD68, anti-Ephb4, anti-Ephrinb2, and anti-Ptgs2 (Cox2). A complete list of antibodies used in this study is provided in Table S1. After washing thrice with PBS, the sections were stained with the appropriate secondary antibodies and 4′,6-diamidino-2-phenylindole (Invitrogen, Cat. No. 62248) for 1 h. Fluorescent images were acquired using a Zeiss LSM980 confocal microscope.

### 3D microfluidic chip

The design of the microfluidic chip and manufacturing method have been reported in a previous study [30]. Briefly, SU-8 photoresist with a thickness of 120 to 130 μm was first photolithographed and patterned on a silicon wafer. Polydimethylsiloxane mixture (PDMS, SYLGARD 184 Silicone Elastomer Kit, Dow Corning) was prepared by mixing the curing agent and PDMS prepolymer in a weight ratio of 1:10. The PDMS mixture was poured onto the patterned wafer. Subsequently, the PDMS mixture was degassed in a vacuum chamber for 15 min to remove bubbles. The PDMS mixture was baked in a drying oven at 80°C for 4 h. The cured PDMS mold was detached, trimmed, and punched by a biopsy punch and blunt needle. The PDMS mold was autoclaved at 120°C for 30 min. The sterilized PDMS mold (upper part) and glass coverslip (bottom part) were bonded after oxygen plasma treatment (FEMTO science). Immediately, the bonded microchips were filled with poly-d-lysine solution (1 mg/mL, Sigma-Aldrich) and incubated at 37°C for 2 h. Subsequently, the microchip was washed twice with deionized water. The microchip was then dried in an oven at 80°C for over a day.

### Kinome-wide siRNA library screening

A kinome-wide siRNA screen was conducted using the Dharmacon siGENOME mouse protein kinases siRNA library (0.1 nmol; Dharmacon, Cat. No. G-013505-01) in 96-well plates, targeting 713 genes with 4 pooled siRNAs each. For transfection, 0.2 μL of Lipofectamine RNAiMAX Transfection Reagent (Invitrogen, Cat. No. 13778030) was combined with 19.8 μL of library-siRNA-OptiMEM (Gibco, Cat. No. 31985070) solution in each well and incubated for 30 min at room temperature. Subsequently, 1 × 10^4^ luciferase-tagged MC38 (MC38-luc) cells and 5 × 10^4^ BMDMs, collected on day 7 of differentiation, were seeded in 40 μL of OptiMEM onto the predispensed transfection mixture, resulting in a final concentration of 50 nmol/L pooled siRNA in a total volume of 100 μL. After 12 h of incubation at 37°C, IR (6 Gy) was applied to the plates in the IR-positive group (irradiated group), whereas plates in the IR-negative group (nonirradiated control group) did not receive IR. The IR dose (6 Gy) was selected based on the experimental conditions, including differences in cell number and the use of in vitro or in vivo experimental systems. Following 6 d of further incubation at 37°C, IVIS luminescence analysis was performed using the IVIS Spectrum Imaging System (PerkinElmer) after adding 100 μL of luciferin to each well. The library screening was conducted twice for both IR-positive and IR-negative groups. Each plate contained 8 negative control wells without siRNA, and the relative luminescence intensity (RLU) of each experimental well was normalized to the average intensity value of the negative control wells on the same plate.

### Immunohistochemistry

For immunohistochemical analysis, formalin-fixed, paraffin-embedded tissue samples were collected from pretreatment endoscopic biopsy specimens and posttreatment surgical resection specimens. Tissue microarrays were constructed using representative tumor cores (2 mm in diameter) obtained exclusively from tumor areas of donor formalin-fixed, paraffin-embedded blocks and rearranged into recipient blocks using a trephine apparatus (SuperBioChips Laboratories), as previously described [31]. Tissue microarray sections were stained for EPHB4 (1:100; Invitrogen, Cat. No. 37-1800) using the Ventana BenchMark ULTRA IHC/ISH system (Ventana Medical Systems). Staining intensity was scored as 0 (none), 1 (weak), 2 (moderate), or 3 (strong), and the proportion of positively stained tumor area was recorded as a percentage for each intensity level. An H-score was calculated by multiplying the staining intensity by the corresponding tumor area percentage, yielding a total score ranging from 0 to 300 [32]. All immunohistochemical evaluations were performed in a blinded manner to TRG.

### CRISPR/Cas9-mediated gene editing

*Ephb4*-knockout (KO) cells were generated using a dual-cut CRISPR/Cas9 plasmid designed to delete exons 2 and 3 of the *Ephb4* gene (Macrogen). The plasmid comprised 2 sgRNAs targeting the sequences 5′-AGAGACAAAGTCAGCCCCAC-3′ and 5′-CATCAAGGTACCAACAGTCT-3′. Cells were transfected at ~60% confluence with 2 to 2.5 μg of the sgRNA using standard transfection methods. After 48 h, cells were selected with 5 μg/mL puromycin. KO efficiency was confirmed by immunoblotting.

### Cell viability test

Tumor cells were seeded into 96-well plates 24 h prior to IR. Cell viability was assessed at days 1, 3, and 5 post-IRs using the cell counting kit-8 (Dojindo, Cat. No. CK04) according to the manufacturer’s instructions. Optical density was measured at 450 nm using a microplate reader (BioTek).

### Western blotting analysis

For pharmacological inhibition studies, MC38 tumor cells were treated with the hypoxia-inducible factor-1 alpha (HIF-1α) inhibitor YC-1 (50 μmol/L; Selleck Chemicals, Cat. No. S7958), followed by IR (10 Gy). Ephb4 expression was analyzed by immunoblotting 48 h post-IR.

Cells were lysed in radioimmunoprecipitation assay lysis buffer containing protease inhibitor (GenDEPOT, Cat. No. P3100-005) and phosphatase inhibitor cocktail (GenDEPOT, Cat. No. P3200-005). Cell lysates were separated on Novex 8% to 16% tris-glycine gels (Invitrogen, Cat. No. XP08165BOX) and transferred onto polyvinylidene fluoride membranes using Power Blotter Select Transfer Stacks (Invitrogen, Cat. No. PB5310). The membranes were blocked in blocking buffer (PBS with 5% skim milk and 0.05% Tween 20) for 1 h at room temperature, followed by incubation overnight at 4°C with primary antibodies against Ephb4, protease-activated receptor 2 (Par2), solute carrier family 7 member 11 (Slc7a11), solute carrier family 3 member 2 (Slc3a2), glutathione peroxidase 4 (Gpx4), Tlr2, activating transcription factor 4 (Atf4), phospho-nuclear factor-kappa B (NF-κB) p65, NF-κB p65, and β-actin. A complete list of antibodies used in this study is provided in Table S1. The following day, the membranes were incubated with horseradish peroxidase-conjugated secondary antibodies. Chemiluminescence was detected with an Amersham Imager 680 instrument (GE Healthcare).

### Flow cytometry

Single-cell suspensions from mouse tumor tissues or cells cultured in vitro were incubated with purified anti-CD16/CD32 (eBioscience, Cat. No. 14-0161-82) for 30 min at 4°C and finally processed for cell-surface staining with the appropriate antibodies at 4°C. For intracellular staining, cells were fixed and permeabilized using Perm/Wash buffer (eBioscience, Cat. No. 00-5523-00) and finally labeled with the appropriate secondary antibodies. The following antibodies were used: CD45 allophycocyanin (APC)-Cy7 (clone 30-F11), CD11b eFluor 450 (clone M1/70), Ly6G fluorescein isothiocyanate (FITC) (clone 1A8-Ly6g), Ly6C PerCP-Cy5.5 (clone HK1.4), F4/80 phycoerythrin (PE) (clone BM8), CD4 PE (clone GK1.5), Foxp3 V450 (clone FJK-16s), Granzyme B PE-Cy7 (clone QA18A28) and major histocompatibility complex (MHC) Class II APC (clone M5/114.15.2) from eBioscience; CD11c BV605 (clone N418), F4/80 FITC (clone BM8), CD3 FITC (clone 17A2), CD3 PE-Cy7 (clone 17A2), CD206 PE (clone C068C2), interleukin-6 (IL-6) APC (clone MP5-20F3), Tlr2 BV421 (clone CB225), and interferon-γ (IFN-γ) PerCP-Cy5.5 (clone XMG1.2) from BioLegend; CD45 APC (clone 30-F11) and CD8a BV711 (clone 53-6.7) from BD Biosciences.

Cell death was assessed by staining with 7-aminoactinomycin D (7-AAD) (BioLegend, Cat. No. 640934) according to the manufacturer’s instructions. For ex vivo analysis of tumor tissues, tumors were mechanically dissociated to generate single-cell suspensions and stained with anti-CD45 antibody (eBioscience, Cat. No. 47-0451-82), and analyses were performed on the CD45^−^ tumor cell-enriched population. In macrophage–tumor cell coculture experiments, cells were similarly stained with anti-CD45 antibodies to allow tumor cell-specific analysis, and CD45^−^ cells were gated as tumor cells for subsequent analysis. Cell death was quantified as the percentage of 7-AAD^+^ cells within the CD45^−^ population. Tumor cell proliferation was assessed by Ki67 staining (Cell Signaling Technology, Cat. No. 12202S) using the same gating strategy, and Ki67^+^ cells were quantified within the CD45^−^ population.

Intracellular iron was detected using BioTracker 575 Red Fe^2+^ (Merck, Cat. No. SCT030; also named as FeRhoNox-1 in the figure legend) according to the manufacturer’s instructions. Cells were subsequently stained with anti-CD45 antibody, and intracellular iron levels were analyzed in the CD45^−^ tumor cells by flow cytometry. A complete list of antibodies used in this study is provided in Table S1.

Data were acquired using the BD LSRFortessa Cell Analyzer or BD Symphony A3 Cell Analyzer (BD Biosciences) and analyzed using the FlowJo software (version 10.10.1). The gating strategy for tumor tissue-derived single-cell suspensions is shown in Fig. S1.

### BODIPY-C11 staining

For in vitro assays, 3 × 10^4^ tumor cells were seeded into 12-well plates with or without 3 × 10^5^ macrophages, and the plates were irradiated (10 Gy) the following day. On day 3 post-IR, cells were incubated with 5 μmol/L BODIPY 581/591 C11 (Thermo Fisher Scientific, Cat. No. D3861) in DMEM for 30 min at 37°C. Cells were then washed, dissociated with trypsin, neutralized with DMEM, and prepared for flow cytometry. To specifically quantify lipid peroxidation in tumor cells within the coculture, dissociated cells were stained with anti-CD45 antibodies, and tumor cells were defined as the CD45^−^ population.

For ex vivo analysis, tumor tissues were mechanically dissociated to generate single-cell suspensions and incubated with 10 μmol/L BODIPY 581/591 C11 for 40 min at 37°C. To enrich tumor cells, samples were subsequently stained with anti-CD45 antibodies, and analyses were performed on the CD45^−^ tumor cell-enriched population. Data were acquired using an LSRFortessa flow cytometer (BD Biosciences), measuring both the nonoxidized form (PE channel) and oxidized form (FITC channel) of the dye. Lipid peroxidation was quantified by calculating the ratio of the mean fluorescence intensity of FITC to PE. All values were normalized to control samples and reported as relative lipid reactive oxygen species (ROS) levels.

### In vivo tumor model and treatment

MC38 (1 × 10^6^ cells), MC38-GFP (1 × 10^6^ cells), or CT26 (5 × 10^5^ cells) tumor cells were subcutaneously injected into the right flank of 8-week-old female C57BL/6 (for MC38 and MC38-GFP) or BALB/c (for CT26) mice. Tumor-localized IR (single 10-Gy dose) was administered on day 7 postimplantation. As tumor growth divergence between WT and *Ephb4*-KO tumors became evident from day 7 post-IR, immune profiling and mechanistic analyses were performed at time points following day 7 post-IR.

For IL-6 neutralization, tumor-bearing mice were treated intraperitoneally with 300 μg of anti-IL-6 neutralizing antibody (Bio X Cell, Cat. No. BE0046) or isotype control 3 times per week for 14 d. For CD8 neutralization, tumor-bearing mice were treated intraperitoneally with 150 μg of anti-CD8 neutralizing antibody (Bio X Cell, Cat. No. BE0061) or isotype control 3 times per week for 14 d. For EphB4 inhibition, mice were administered 50 mg/kg of NVP-BHG712 (MedChemExpress, Cat. No. HY-13258A) or vehicle by daily oral gavage for 14 d, starting 1 d prior to IR. To inhibit ferroptosis, mice were treated with 30 mg/kg liproxstatin-1 (Sigma-Aldrich, Cat. No. SML1414) or vehicle by daily intraperitoneal injection, beginning 3 d prior to IR and continuing through day 14 after IR.

### RNA sequencing

For tumor cell RNA sequencing (RNA-seq), tumor cells were enriched after coculture with macrophages by magnetic negative selection using anti-CD45 microbeads (Miltenyi Biotec, Cat. No. 130-052-301). For macrophage RNA-seq, TAMs were freshly isolated by fluorescence-activated cell sorting from irradiated MC38 tumors.

Total RNA was isolated using TRIzol reagent (Invitrogen). RNA quality was assessed by the Agilent TapeStation 4000 system (Agilent Technologies), and RNA quantification was performed using the ND-2000 Spectrophotometer (Thermo Fisher Scientific). For control and test RNAs, the construction of the library was performed using QuantSeq 3′ mRNA-Seq Library Prep Kit (Lexogen, Cat. No. 015) according to the manufacturer’s instructions. Briefly, total RNA was prepared, and an oligo-dT primer containing an Illumina-compatible sequence at its 5′ end was hybridized with the RNA, followed by reverse transcription. After degradation of the RNA template, second-strand synthesis was initiated by a random primer containing an Illumina-compatible linker sequence at its 5′ end. The double-stranded library was purified using magnetic beads to remove all reaction components. The library was amplified to add the complete adapter sequences required for cluster generation. The finished library was purified from polymerase chain reaction (PCR) components. High-throughput sequencing was performed as single-end 75 sequencing using the NextSeq 550 System (Illumina). QuantSeq 3′ mRNA-Seq reads were aligned using Bowtie2 [33]. Bowtie2 indices were either generated from the genome assembly sequence or the representative transcript sequences for aligning to the genome and transcriptome (version UCSC mm 10). The alignment file was used for assembling transcripts, estimating their abundances, and detecting differential expression of genes. Differentially expressed genes were determined based on counts from unique and multiple alignments using coverage in Bedtools [34]. The Read Count data were processed based on the normalization method using the EdgeR package (version 3.42.4) in R (version 4.3.0) [35].

### Quantitative real-time PCR

Total RNA was extracted using the TRIzol reagent (Invitrogen) according to the manufacturer’s instructions. Complementary DNA was synthesized from 1 μg of total RNA using reverse transcription, and the amount of mRNA was quantified using real-time PCR analysis with SYBR Green qPCR Pre-Mix (Enzynomics, Cat. No. RT500) on an ABI real-time PCR 7500 machine (Applied Biosystems). Gene expression was normalized to the housekeeping gene 18S ribosomal RNA. The primer sequences are listed in Table S2.

### Lipidomic profiling

#### Sample preparation and lipid extraction

Cell pellets were washed 3 times with PBS. To ensure equal sample input for lipidomics, one-tenth of each cell pellet was aliquoted for lysis and protein quantification. Total protein concentration was measured using a bicinchoninic acid assay (Thermo Fisher Scientific, Cat. No. 23235) according to the manufacturer’s instructions, and lipid extraction was performed using 50 μg of total protein-equivalent material per sample.

Lipid extraction was conducted using a modified chloroform/methanol method designed to recover both neutral and acidic lipids [36]. Prior to extraction, an internal standard mixture was added to each sample. The internal standard mixture consisted of class-representative odd-chain phospholipid standards, including lysophosphatidylcholine 13:0 and phosphatidylcholine 17:0 for positive ion mode and phosphatidylserine (PS) 17:0/17:0 and lysophosphatidylserine 17:1 for negative ion mode, which were used for signal normalization.

For neutral lipid extraction, 300 μL of chloroform:methanol (1:2, v:v) was added to the pellet, followed by tip sonication 3 times for 5 s with 30-s cooling intervals on ice. An additional 700 μL of chloroform:methanol (1:2, v:v) was added, and samples were vortexed for 1 min, incubated on ice for 10 min, and centrifuged at 13,800 ×*g* for 2 min at 4°C. The supernatant was collected. For acidic lipid extraction, the remaining pellet was resuspended in 750 μL of chloroform:methanol:37% HCl (40:80:1, v:v:v), vortexed for 1 min, and incubated at room temperature for 15 min. Next, 250 μL of ice-cold chloroform and 450 μL of 0.1 mol/L HCl were added, followed by centrifugation at 6,500 ×*g* for 2 min. The bottom organic layer was collected and combined with the neutral lipid extract.

Combined organic phases were dried using a speed vacuum concentrator, and dried lipid extracts were stored at −80°C until mass spectrometry analysis.

#### Liquid chromatography-tandem mass spectrometry analysis

Targeted lipidomic profiling was performed using dynamic multiple reaction monitoring on a TSQ Altis Plus triple quadrupole mass spectrometer (Thermo Fisher Scientific) coupled to a Vanquish Flex UHPLC system (Thermo Fisher Scientific). Approximately 2,000 candidate multiple reaction monitoring (MRM) transitions were initially designed to cover major lipid classes. Following lipid extraction, 10% aliquots of each sample were pooled and used for transition optimization. Transitions were evaluated based on the following criteria: (a) signal intensity with a signal-to-noise ratio ≥ 3 relative to background; (b) chromatographic peak shape assessed using the dot product score in Skyline, with a threshold of ≥0.7; and (c) reproducibility, defined as a coefficient of variation < 20% of standards across replicate injections. Transitions with a peak area below 1,000 counts were considered low-quality and excluded. A total of 768 optimized transitions were selected for final acquisition.

All transitions were acquired in scheduled (dynamic) MRM mode. Key MRM acquisition parameters were as follows: Q1 and Q3 resolution (full width at half maximum) of 0.7 Da, collision gas pressure of 1.5 mTorr, cycle time of 3.0 s, chromatographic peak width of 6.0 s, minimum dwell time of 10 ms, and data mode set to centroid.

Lipid species were analyzed in separate ionization modes to maximize analytical coverage and sensitivity. Anionic phospholipids, including phosphatidylglycerol, phosphatidylinositol, PS, phosphatidic acid, and selected ceramide species, were analyzed in negative ion mode using a hydrophilic interaction liquid chromatography column (ZORBAX HILIC, 100 × 2.1-mm internal diameter, 1.8-μm particle size; Agilent Technologies). Neutral and zwitterionic lipid species, including phosphatidylcholine, phosphatidylethanolamine, monoacylglycerol, sterols, sphingomyelin, ceramide, triacylglycerol, and diacylglycerol, were analyzed in positive ion mode using a Hypersil GOLD column (100 × 2.1-mm internal diameter, 1.9-μm particle size; Thermo Fisher Scientific).

For reverse-phase separation (positive ion mode), the mobile phase consisted of solvent A (methanol:acetonitrile:water, 19:19:2, v:v:v, containing 20 mmol/L ammonium formate and 0.1% formic acid) and solvent B (isopropanol containing 20 mmol/L ammonium formate and 0.1% formic acid). For hydrophilic interaction liquid chromatography separation (negative ion mode), the mobile phase consisted of solvent A (acetonitrile:water, 95:5, v:v, containing 10 mmol/L ammonium acetate) and solvent B (acetonitrile:water, 50:50, v:v, containing 10 mmol/L ammonium acetate). The same gradient profile was applied in both modes, with solvent B increased from 5% to 90% over a 30-min run (0 to 5 min, 5% solvent B; 5 to 15 min, 5% to 30% solvent B; 15 to 22 min, 30% to 90% solvent B; 22 to 25 min, 90% solvent B; 25 to 26 min, 90% to 5% B (rapid return to initial conditions); 26 to 30 min, 5% solvent B (column re-equilibration), at a constant flow rate of 0.300 mL/min.

The mass spectrometer was operated in heated electrospray ionization mode with spray voltages of 3.5 kV (positive mode) and 2.5 kV (negative mode), ion transfer tube temperature of 275°C, vaporizer temperature of 350°C, sheath gas set to 50 (arb units), auxiliary gas set to 10 (arb units), collision gas pressure of 1.5 mTorr, and Q1 and Q3 resolution of 0.7 full width at half maximum.

#### Data processing, normalization, and statistical analysis

Raw liquid chromatography-tandem mass spectrometry (LC-MS/MS) data were processed in Skyline to integrate MRM peak areas for each transition. Peak areas were exported, and subsequent normalization, merging of positive/negative mode datasets, and statistical analyses were performed in R (version 4.4.5). Because lipid classes were acquired in separate polarity/LC conditions, normalization was performed independently for positive-mode and negative-mode datasets prior to merging. For normalization, intensities were normalized using max-based scaling with the internal standard (ISTD). Specifically, the summed ISTD area was calculated per sample, and each sample was scaled by the factor (max ISTD area across samples/ISTD area of that sample) to obtain normalized abundances. Normalized positive and negative lipid results were subsequently merged into a single matrix for downstream analyses. For multivariate analysis, normalized abundances were aggregated at the molecule level, and a feature-by-sample matrix was generated. Prior to principal component analysis, features with zero variance were removed, and an additional detection filter was applied such that only features detected (value > 0) in at least 30% of samples were retained. Principal component analys was performed on the log1p-transformed matrix using the prcomp function in R (stats package, R version 4.4.1), with centering and scaling enabled. Group separation was visualized using PC1 and PC2 scores. For differential abundance analysis between groups (WT vs. *Ephb4*-KO), comparisons were performed separately for the indicated subsets. For each lipid species, group-wise mean abundance was calculated, and log_2_ fold change was computed as log_2_[(mean_KO + 1) / (mean_WT + 1)] using a pseudocount of 1. Statistical significance was evaluated on log1p-transformed abundances using 2-sided Wilcoxon rank-sum tests (nonparametric). Lipids were considered significant based on a nominal *P* < 0.05. For lipid compositional summaries, lipid class was parsed from molecule identifiers, and total double bond number was calculated by summing the double bonds across acyl chains extracted from the annotation string. The proportion of polyunsaturated fatty acyl chain-containing lipids was compared between groups using Fisher’s exact test, and differences in unsaturation burden (total double bonds) were assessed using the Wilcoxon rank-sum test. *P* < 0.05 was considered statistically significant.

### Establishment of IR-resistant cell lines

To establish IR-resistant MC38 tumor cell lines, 5 × 10^5^ MC38 cells and 5 × 10^6^ BMDMs were coseeded into 100-mm cell culture dishes. After 24 h of coculture, cells were exposed to 10-Gy IR. Cultures were maintained for 6 d post-IR, with medium changed every 2 d. On day 6, CD45^+^ cells were depleted using anti-CD45 magnetic microbeads (Miltenyi Biotec) to isolate tumor cells. The recovered MC38 cells were then reseeded with freshly prepared BMDMs under identical conditions, followed by 10-Gy IR and another 6-d culture period. This cycle of macrophage coculture, IR, and tumor cell reisolation was repeated for a total of 10 rounds. Tumor cells isolated after the final cycle were maintained as IR-resistant MC38 populations for downstream analyses.

In parallel, tumor cell-only IR-resistant lines were generated by seeding MC38 cells alone at the same density and subjecting them to the identical IR and culture schedule, including serial reseeding and 10 rounds of selection, but in the absence of BMDMs. To assess the acquisition of radiation resistance, parental and IR-resistant tumor cells were irradiated with 10 Gy in the absence of macrophages, and cell death was quantified by 7-AAD staining followed by flow cytometry.

### Enzyme-linked immunosorbent assay

Levels of IL-6 and cathepsin S (Ctss) were measured in culture supernatants and tumor homogenates using a Mouse IL-6 DuoSet enzyme-linked immunosorbent assay (ELISA) kit (R&D Systems, Cat. No. DY406) and a Mouse CTSS ELISA kit (Cusabio Biotech, Cat. No. CSB-EL006204MO), respectively, following the manufacturers’ instructions.

### *Efnb2* knockdown in BMDMs

Gene knockdown was performed using P2 Primary Cell 4D-Nucleofector X Kit (Lonza, Cat. No. V4XP-2012) according to the manufacturer’s instructions with modification. Briefly, 2 × 10^6^ BMDMs in a cuvette were transfected with 500 pmol of either *Efnb2*-targeting siRNA (Dharmacon Accell SMARTpool, Cat. No. E-051250-00-0020) or nontargeting control siRNA (Dharmacon Accell nontargeting pool, Cat. No. D-001910-10-20) using the “Mouse, macrophage” preset program on the 4D-Nucleofector (Lonza). Transfected cells were cultured for 48 h prior to coculture with tumor cells and subsequent IR.

### Clonogenic survival assay

MC38 cells (3 × 10^4^) were cocultured with BMDMs (3 × 10^5^) in 6-well plates in triplicate and allowed to adhere overnight. The following day, cultures were pretreated with anti-IL-6 antibodies, followed by exposure to 10-Gy IR. Culture media were replaced every 2 d. After 10 d of incubation, colonies were fixed and stained with 0.05% crystal violet solution (Abcam, Cat. No. AB246816) and were counted manually.

### Proteomic profiling

#### Sample preparation, tandem mass tag labeling, and high-pH fractionation

MC38 tumor cells were cocultured with BMDMs and treated with either vehicle or NVP-BHG712 (NVP), followed by IR (10 Gy). On day 2 post-IR, conditioned media (CM) were collected for proteomic analysis. A total of 100 μg of protein from each sample was processed and digested using the S-Trap Micro spin column protocol (Protifi, Cat. No. C002-MICRO-0040PK). Proteins were first reduced using 5 mmol/L dithiothreitol (DTT; Thermo Fisher Scientific) for 30 min at 37°C and alkylated with 20 mmol/L iodoacetamide (IAA; Thermo Fisher Scientific) at room temperature in the dark for 30 min. Digestion was performed on-column with sequencing-grade modified trypsin (Thermo Fisher Scientific) at a 1:50 enzyme-to-substrate ratio (w:w), overnight at 37°C.

After digestion, peptides were eluted and dried under vacuum. Tandem mass tag (TMT) 6-plex labeling (TMTsixplex, Thermo Fisher Scientific) was performed according to the manufacturer’s instructions. Labeled peptides were then pooled into a single tube and dried via vacuum centrifugation. The combined sample was subjected to high-pH reversed-phase fractionation using an Ultimate 3000 HPLC system (Thermo Fisher Scientific). Peptides were separated on a C18 reversed-phase column (25 cm × 4.6 mm, 5 μm, 300 Å, Waters) into 24 fractions using a 115-min gradient.

#### LC-MS/MS acquisition

Each of the 24 fractions was desalted using in-house prepared StageTips packed with Empore C18 disks (3M) and analyzed on a Vanquish Neo UHPLC system (Thermo Fisher Scientific) coupled to an Orbitrap Exploris 240 mass spectrometer (Thermo Fisher Scientific). Peptides were separated on a C18 column (50 cm × 75 μm, 2 μm, 100 Å, Thermo Fisher Scientific). A linear gradient of mobile phase B (80% acetonitrile with 0.1% formic acid) was applied from 2% to 34% B over 108 min, followed by an increase to 90% at 109 min. The composition was maintained until 120 min at a flow rate of 300 nL/min.

The mass spectrometer was operated in data-dependent acquisition (DDA) mode. Full MS scans were acquired at a resolution of 120,000 over a mass range of 350 to 1,500 m/z. MS/MS spectra were acquired with a cycle time of 3 s at a resolution of 45,000 using higher-energy collisional dissociation (HCD) with a normalized collision energy of 36%. The dynamic exclusion duration was 30 s.

#### Database search and quantification

Raw files were analyzed using FragPipe (version 22.0) with MSFragger (version 4.1) as the search engine. Peptides were searched against the UniProt Mus musculus reference proteome database (Released in 2025.03). Search parameters included: full tryptic specificity, up to 2 missed cleavages, precursor mass tolerance of 20 ppm, and fragment ion tolerance of 0.02 Da. Static modifications were set as TMT 6-plex on lysine residues and peptide N-termini, and carbamidomethylation of cysteine; methionine oxidation was set as a variable modification. Peptide and protein-level identifications were filtered at a 1% false discovery rate. Reporter ion quantification was performed using IonQuant (part of the FragPipe suite), with TMT 6-plex reporter ion intensities extracted from MS/MS spectra.

#### Statistical and functional analysis

For quantitative analysis, only proteins identified with at least 2 peptides (including unique and razor peptides) were included to ensure reliability of quantification. Quantitative protein data were normalized using median normalization. Statistical comparisons between groups were performed using unpaired 2-tailed Student’s *t* tests in R (version 4.4.5). Differentially expressed proteins were defined as those with a *P* < 0.05 and a fold change > 1.5 or < 0.67 and were subsequently used for downstream functional analysis. Single-sample gene set enrichment analysis was performed using the GSVA package. Functional enrichment analysis was conducted using curated mouse gene sets from the Molecular Signatures Database (MSigDB, version v2025.1.Mm), including Gene Ontology (Biological Process, Cellular Component, and Molecular Function), Reactome, and WikiPathways annotations.

#### Protein–protein interaction network construction and clustering analysis

Protein–protein interaction networks were constructed using Cytoscape (version 3.10.2). Interaction data were obtained from the STRING database (version 12.0), with the interaction source restricted to “full STRING network” but excluding text mining associations. To identify functional modules within the network, K-means clustering was performed based on the negative logarithm of adjusted *P* values derived from prior statistical analyses (e.g., differential expression or enrichment analyses). The optimal number of clusters (*k* = 7) was determined empirically. Each cluster was subsequently annotated with a representative biological process or function, based on enrichment analysis of the clustered proteins. Betweenness centrality was calculated in Cytoscape using the NetworkAnalyzer plugin, which computes the centrality of nodes based on the formula:

$$g\left(v\right)=\sum_{s\neq v\neq t}\frac{\sigma_{\mathrm{st}}\left(v\right)}{\sigma_{\mathrm{st}}}$$ (1)

### The Cancer Genome Atlas expression analysis

*EPHB4* mRNA expression levels across tumor and matched normal tissues were analyzed using the gene expression profiling interactive analysis 2 (GEPIA2) web server (http://gepia2.cancer-pku.cn/), which integrates RNA-seq data from The Cancer Genome Atlas and the Genotype-Tissue Expression (GTEx) project. Expression values were presented as log_2_[transcripts per million (TPM) + 1].

### In silico validation using colon cancer datasets postradiation therapy

Genomic datasets of colon or rectal cancer tumors harvested from patients who underwent radiation therapy were obtained from the Gene Expression Omnibus database: GSE87211 (Agilent-026652 Whole Human Genome Microarray 4x44K v2), GSE190826 (RNA-seq), and GSE186940 (RNA-seq). Only samples from patients who received radiation therapy were included in the analysis. For GSE87211, raw microarray data were processed with the Agilent Feature Extraction software for quality control, normalized using the limma package in R with quantile normalization, and filtered to remove low-expression probes using the filterByExpr function with default parameters from the limma package. For GSE190826, raw RNA-seq data underwent quality control using FastQC v0.11.9, followed by trimming with Trimmomatic v0.39 and alignment to the human reference genome (GRCh38) using STAR v2.7.6a. Gene expression levels were quantified using RSEM v1.3.3, generating TPM values for downstream analysis. Normalization of expression data was conducted using the DESeq2 package in R. For GSE186940, normalized TPM data were downloaded and used for further analysis. Nineteen ferroptosis-related genes (glutathione peroxidase 4 [*GPX4*], solute carrier family 7 member 11 [*SLC7A11*], glutathione synthetase [*GSS*], nuclear factor erythroid 2-related factor 2 [*NFE2L2*], nuclear factor erythroid 2-related factor 1 [*NFE2L1*], yes-associated protein 1 [*YAP1*], apoptosis-inducing factor, mitochondria-associated 2 [*AIFM2*], GTP cyclohydrolase 1 [*GCH1*], 6-pyruvoyltetrahydropterin synthase [*PTS*], prominin-1 [*PROM1*], prominin-2 [*PROM2*], ferritin light chain [*FTL*], solute carrier family 3 member 2 [*SLC3A2*], heme oxygenase 1 [*HMOX1*], sequestosome-1 [*SQSTM1*], glutamate-cysteine ligase catalytic subunit [*GCLC*], glutamate-cysteine ligase regulatory [modifier] subunit [*GCLM*], and peroxiredoxin-6 [*PRDX6*]), which were curated from the FerrDb database [37] and further filtered based on preserved coexpression in ferroptosis signaling (Pearson *r* ≥ 0.5, *P* < 0.05), were selected. Proliferation-related genes were retrieved from the Gene Ontology Biological Process term “cell proliferation” (GO:0008283). For each gene set, expression levels were normalized using *z*-score transformation, and mean *z*-scores were calculated to generate ferroptosis and proliferation scores for each sample. Samples were then divided into ferroptosis-sensitive (ferroptosis-S) and ferroptosis-resistant (ferroptosis-R) groups based on the median scores. Samples with scores less than the median were assigned to the sensitive group, whereas those with scores greater than the median were assigned to the resistance group. Differential gene expression analysis involved comparing expression levels of *EPHB4*, *GPX4*, *SLC7A11*, *SLC3A2*, and *IL6* between these groups using box plots, with statistical significance assessed via the Mann–Whitney U test and multiple comparison corrections made using the Benjamini–Hochberg method. Pearson correlation coefficients were calculated to assess relationships between *EPHB4* expression and ferroptosis-related genes and *IL6* within the datasets, visualized using scatter plots and heatmaps. Odds ratios for associations between gene expression (*EPHB4*, *GPX4*, *SLC7A11*, *SLC3A2*, and *IL6*) and high ferroptosis or high proliferation status were calculated, and 95% confidence intervals were reported. All analyses were performed using R version 4.4.1, with visualization accomplished using ggplot2 and corrplot packages, and statistical testing conducted using the stats package.

### Statistical analysis

All statistical analyses were performed using GraphPad Prism software (version 9.0). Differences between the 2 groups were evaluated using unpaired 2-tailed Student’s *t* tests. For comparisons involving more than 2 groups, 1-way analysis of variance (ANOVA) or 2-way ANOVA was used. Data are presented as means ± standard error of the mean (SEM), unless otherwise indicated. Statistical significance was denoted in figures as follows: *P < 0.05, **P < 0.01, ***P < 0.001.

## Results

### Macrophage contact enabled tumor cell survival and proliferation after IR

**Fig. 1.**
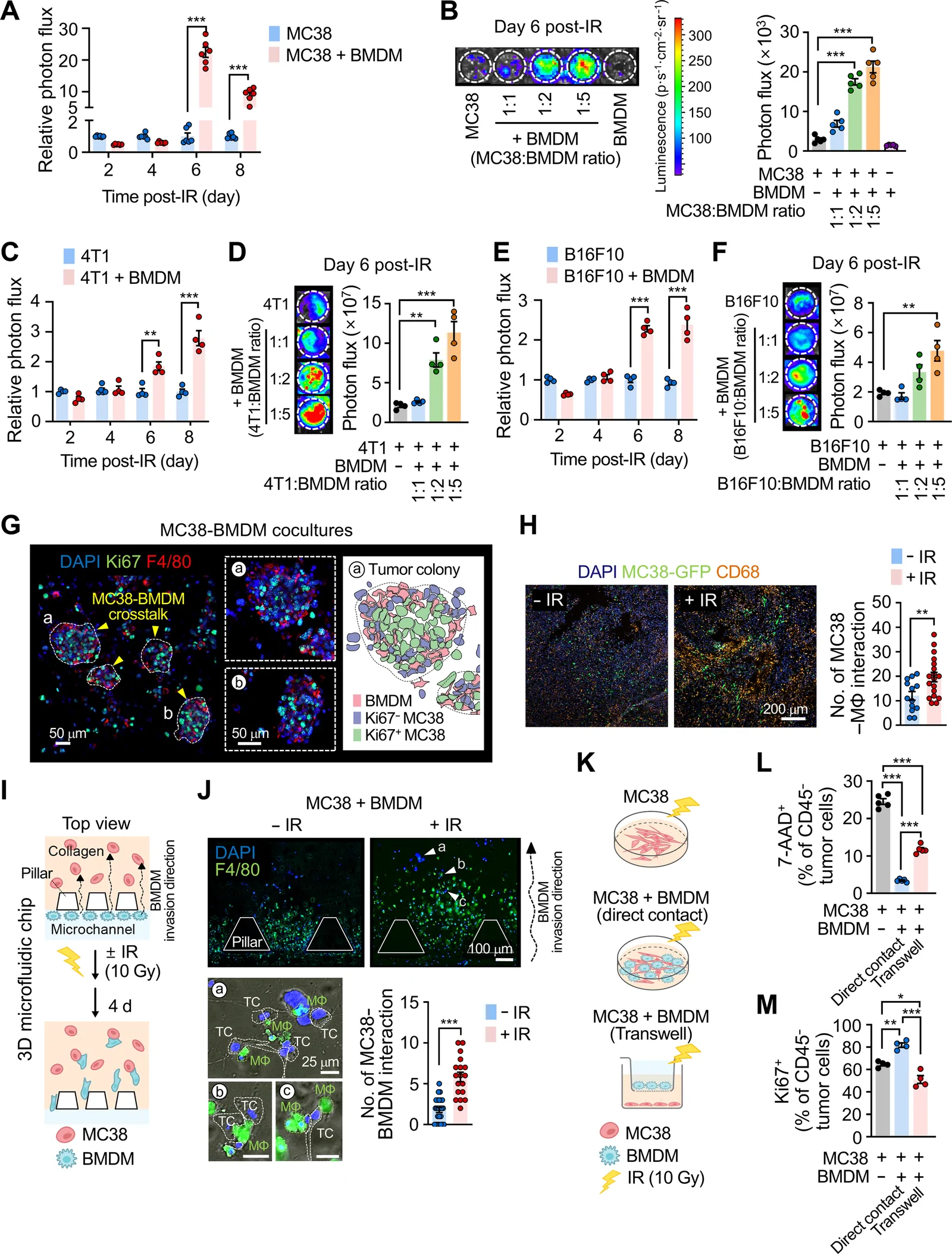
Macrophages promote sustained tumor cell survival following irradiation (IR) through contact-dependent mechanisms. (A) Bioluminescence signals of luciferase-expressing MC38 tumor cells (MC38-luc) cocultured with or without bone marrow-derived macrophages (BMDMs) (MC38-to-BMDM ratio, 1:5) following IR (6 Gy). Luminescence signals were measured at the indicated time points (day 2, 4, 6, and 8 post-IR). (B) Representative luminescence images and photon flux quantification of MC38-luc cells cocultured with BMDMs at different MC38-to-BMDM ratios (1:1, 1:2, and 1:5), as well as MC38-luc cells alone and BMDMs alone, at day 6 post-IR. (C) Bioluminescence signals of 4T1-luc cocultured with or without BMDMs (4T1-to-BMDM ratio, 1:5) following IR (6 Gy). Luminescence signals were measured at the indicated time points (day 2, 4, 6, and 8 post-IR). (D) Representative luminescence images and photon flux quantification of 4T1-luc cells cocultured with BMDMs at different 4T1-to-BMDM ratios (1:1, 1:2, and 1:5), as well as 4T1-luc cells alone, at day 6 post-IR. (E) Bioluminescence imaging of B16F10-luc cells cocultured with or without BMDMs (B16F10-to-BMDM ratio, 1:5) following IR (6 Gy). Luminescence signals were measured at the indicated time points (day 2, 4, 6, and 8 post-IR). (F) Representative luminescence images and photon flux quantification of 4T1-luc cells cocultured with BMDMs at different B16F10-to-BMDM ratios (1:1, 1:2, and 1:5), as well as B16F10-luc cells alone, at day 6 post-IR. (G) Representative immunofluorescence (IF) staining image of MC38-BMDM cocultures at 6 d post-IR (10 Gy), showing 4′,6-diamidino-2-phenylindole (DAPI) (blue), Ki67 (green), and F4/80 (red) expression. The dotted line indicates representative tumor colonies. The schematic on the right illustrates panel (a), highlighting the accumulation of BMDMs around residual tumor colonies enriched for Ki67-expressing MC38 tumor cells following IR. (H) Representative IF images of tumors derived from MC38-green fluorescent protein (GFP) tumor cells implanted in C57BL/6 mice. Tumors were established by subcutaneous injection and subjected to localized IR (10 Gy) on day 7 (+IR) or left untreated (−IR), followed by tissue collection 14 d after IR. Sections were stained for DAPI (blue) and CD68 (orange), with tumor cells identified by GFP (green). Quantification shows the number of direct contacts per field between tumor cells (MC38-GFP^+^) and tumor-associated macrophages (CD68^+^, MΦ). (I) Schematic illustration of a collagen-embedded 3D microfluidic platform designed to model macrophage recruitment and tumor cell–macrophage interactions following IR. MC38 tumor cells were embedded within a collagen matrix, while BMDMs were seeded in an adjacent microchannel. This configuration allows macrophages to migrate into the collagen matrix toward tumor cells. Following IR, macrophage infiltration and subsequent physical interactions with tumor cells were monitored and compared between irradiated and nonirradiated conditions. (J) Representative IF images from the 3D microfluidic chip platform showing MC38 tumor cells (TC) and BMDMs (MΦ) cocultures 4 d post-IR or nonirradiated. Direct contacts between tumor cells (DAPI^+^F4/80^−^) and macrophage (DAPI^+^F4/80^+^) are indicated by white arrows; Tumor cells are outlined with dashed lines in the enlarged images; Quantification represents the number of tumor cell–macrophage contact events. (K) Schematic of experimental conditions comparing tumor cells cultured alone, direct coculture with BMDMs, and Transwell coculture allowing soluble factor exchange while preventing direct cell–cell contact. (L and M) Percentage of 7-aminoactinomycin D (7-AAD)^+^ dead tumor cells (L, *n* = 5 per group) and Ki67^+^ cells (M, *n* = 4 per group) in CD45^−^ tumor cells 4 d post-IR under 3 conditions in (K): no BMDMs, direct contact coculture, and Transwell-separated coculture. Data are presented as means ± standard error of the mean (SEM) (A to F, H, J, L, and M). *P < 0.05, **P < 0.01, ***P < 0.001, by 2-way analysis of variance (ANOVA) (A, C, and E), 1-way ANOVA with Tukey’s multiple comparison test (B, D, F, L, and M), or unpaired 2-tailed Student’s *t* test (H and J).

To determine whether macrophages contribute to tumor cell survival following IR, we cocultured MC38 colon cancer cells with macrophages and monitored tumor cell growth for 8 d following IR. While irradiated tumor cells cultured alone were largely eliminated, those cocultured with macrophages exhibited sustained growth, and this effect tended to increase with macrophage ratio (Fig. 1A and B). Similar effects were observed in 4T1 breast cancer and B16F10 melanoma cells, indicating that this phenomenon was not restricted to a single tumor type (Fig. 1C to F).

We next assessed whether macrophages enhance tumor cell proliferation after IR. After IR, Ki67 expression was significantly higher in tumor cells cocultured with macrophages than in tumor cells cultured alone (Fig. S2). Notably, Ki67-expressing tumor cells were frequently observed in clusters directly adjacent to macrophages (Fig. 1G), suggesting that physical proximity to macrophages is associated with enhanced tumor cell proliferation. Consistent with this observation, irradiated tumors in vivo exhibited significantly increased spatial association and contact between TAMs and tumor cells compared to nonirradiated tumors (Fig. 1H). To visualize this interaction dynamically, we employed a 3D microfluidic chip for real-time tracking of cell–cell contact (Fig. 1I). Following IR, macrophages migrated toward tumor cells and established contact with them (Fig. 1J).

To investigate whether the macrophage-mediated reduction in tumor cell death and enhancement of tumor cell proliferation depend on direct contact, we compared tumor cell responses in direct versus Transwell-separated cocultures (Fig. 1K). After IR, direct contact with macrophages led to a greater reduction in tumor cell death and a greater increase in proliferation compared with tumor cells cultured alone, whereas Transwell-separated coculture exhibited only a modest effect (Fig. 1L to M). Thus, these findings suggested that IR enhances tumor–macrophage interactions and that this contact-dependent interaction may support tumor cell survival and regrowth following IR.

### Identification of Ephb4 as a key mediator of macrophage-driven radioresistance in tumor cells

**Fig. 2.**
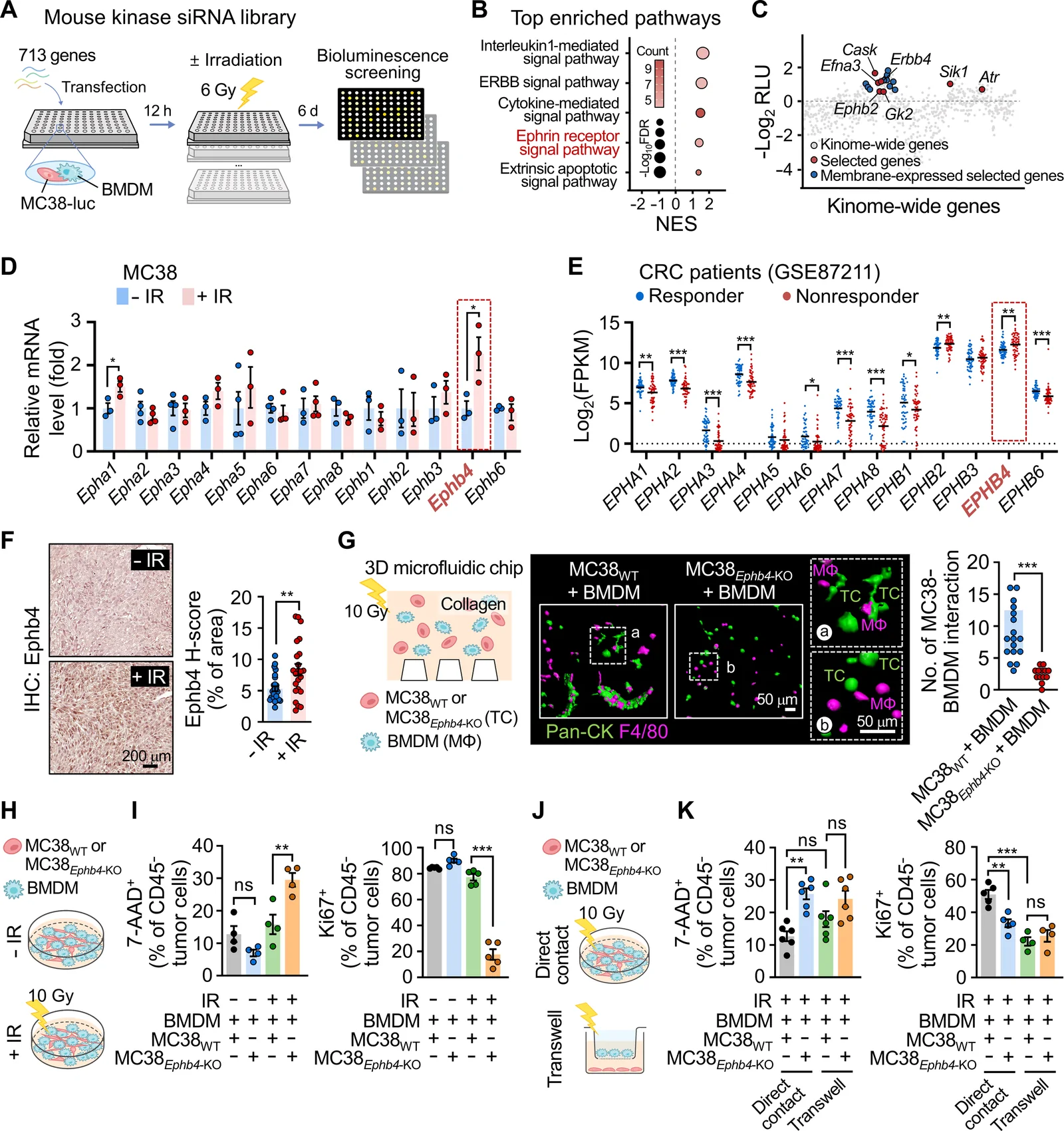
Ephrin receptor b4 (Ephb4) mediated macrophage-induced radioresistance through contact-dependent signaling. (A) Schematic illustration of a kinome-wide small interfering RNA (siRNA) screening performed in the cocultures of MC38-luciferase (luc) tumor cells and bone marrow-derived macrophages (BMDMs) exposed to irradiation (IR) (6 Gy), followed by bioluminescence-based viability readout. (B) Pathway enrichment analysis of the 167 reproducible candidate genes identified from the kinome-wide siRNA screen. The screen was performed independently twice, and genes showing concordant fold change patterns relative to the control in both runs were selected. Enrichment analysis was then performed against signaling pathway gene sets curated from the gene ontology biological process (GO BP) database. (C) Scatter plot of the relative luminescence intensity (RLU) for MC38-luc cells and BMDMs transfected with a kinome-wide library of 713 siRNAs. The x-axis represents the genes targeted by siRNAs, whereas the y-axis displays the −log_2_ RLU values. Radiosensitizer genes were selected when RLU < 0.7 and RLU ratio < 0.6 (blue and red dots). Among the identified genes, membrane-expressed genes are specifically indicated by blue dots. RLU was calculated as luminescence intensity normalized to the negative control well on the same plate; RLU ratio was calculated as the ratio of the RLU values of IR-positive/IR-negative groups of the same gene. (D) Relative mRNA expression of ephrin receptor family genes in MC38 tumor cells 3 h after IR (*n* = 3 to 4 per group), as determined by quantitative polymerase chain reaction. Expression levels were normalized to non-IR MC38 tumor cells for each gene. (E) Transcriptomic analysis of ephrin receptor gene expression in radiotherapy responders (*n* = 51) and nonresponders (*n* = 51) from a colorectal cancer patient cohort (GSE87211). (F) Representative immunohistochemistry (IHC) images (left) and quantification (right) of Ephb4 protein expression in tumors derived from a mouse model with or without IR. MC38 tumor cells were subcutaneously implanted into C57BL/6 mice, followed by IR on day 7, and tumors were analyzed 21 d post-IR (10 Gy). (G) Schematic illustration (left) and representative immunofluorescence images (middle) from a 3D microfluidic platform assessing MC38 tumor cell–BMDM interactions 6 d post-IR (10 Gy). MC38 tumor cells (TC, Pan-cytokeratin [CK], green); BMDMs (macrophage [MΦ], F4/80, magenta). Direct tumor cell–macrophage interactions were defined by manually counting the number of physical contacts between tumor cells and macrophages. The right panel shows quantification of direct tumor cell–BMDM contact events. (H) Schematic illustration of the experimental design. MC38 wild-type (WT) or *Ephb4-*knockout (KO) tumor cells were cocultured with BMDMs with or without IR (10 Gy). (I) Percentage of 7-aminoactinomycin D (7-AAD)^+^ dead cells (*n* = 4 per group) and Ki67^+^ cells (*n* = 5 per group) in CD45^−^ WT or *Ephb4-*KO MC38 tumor cells cocultured with BMDMs, under irradiated 6 d post-IR) and nonirradiated conditions. (J and K) Schematic illustration (J) and percentage of 7-AAD^+^ dead cells (*n* = 6 per group) and Ki67^+^ cells (K) in CD45^−^ tumor cells under direct contact (*n* = 5 per group) vs*.* Transwell-separated (*n* = 4 per group) conditions in WT or *Ephb4-*KO tumor cells 4 d post-IR (10 Gy). Data are presented as means ± standard error of the mean (SEM) (D to G, I, and K). ns, not significant, *P < 0.05, **P < 0.01, ***P < 0.001, 2-way analysis of variance (ANOVA) (D and E), 1-way ANOVA with Tukey’s multiple comparison test (I and K) or unpaired 2-tailed Student's *t* test (F and G). *Atr*, ataxia telangiectasia and rad3-related protein; *Cask*, calcium/calmodulin-dependent serine protein kinase; CRC, colorectal cancer; *Efna3*, ephrina3; *Ephb2*, ephrin receptor b2; ERBB, erythroblastic oncogene B; *Erbb4*, erb-b2 receptor tyrosine kinase 4; FPKM, fragments per kilobase of transcripts per million mapped reads; Gk2, glycerol kinase 2; GSE, gene expression omnibus series accession; NES, normalized enrichment score; *Sik1*, salt-inducible kinase 1;

Building on our finding that direct tumor cell–macrophage contact promoted tumor survival following IR, we sought to identify specific kinases mediating this macrophage-mediated radioresistance. To this end, we performed a kinome-wide siRNA screening using a coculture system of MC38-luc colon cancer cells and BMDMs under irradiated and nonirradiated conditions (Fig. 2A). By measuring post-IR tumor cell viability using bioluminescence, the screen identified 167 reproducible candidate genes with concordant fold change patterns across 2 independent screens. Pathway enrichment analysis of these genes revealed significant overrepresentation of the ephrin receptor signaling pathway (Fig. 2B). Radiosensitizer genes were then defined as those showing reduced viability under IR (RLU < 0.7) together with a decreased RLU ratio between irradiated and nonirradiated conditions (<0.6) (Fig. 2C and Table S3). Given that ephrin receptor–ligand interactions are activated by cell–cell contact and have been implicated in tumor progression and therapy resistance [38], we hypothesized that this pathway mediates contact-dependent crosstalk between macrophages and irradiated tumor cells. Among ephrin receptors, *Ephb4* mRNA was most strongly up-regulated in tumor cells following IR, with a modest increase in ephrin receptor a1 (*Epha1*) (Fig. 2D). Further analysis of colorectal cancer patient data (GSE87211) revealed significantly higher mRNA expression of ephrin receptor B2 (*EPHB2*) and *EPHB4* in RT nonresponders compared to responders (Fig. 2E). However, subsequent functional inhibition experiments indicated that macrophage-mediated tumor cell survival after IR was selectively associated with Ephb4 rather than Ephb2 (Fig. S3). This observation, together with our in vitro finding that Ephb4 is up-regulated in tumor cells following IR, led us to prioritize Ephb4 for further study. At the cellular level, IR significantly increased Ephb4 protein expression in tumor cells regardless of macrophage presence (Fig. S4A), suggesting that Ephb4 up-regulation is primarily driven by a tumor cell-intrinsic response to IR. Because radiation is known to induce hypoxic stress and HIF-1α signaling [39,40], we next examined whether this pathway contributes to Ephb4 induction. Pharmacological inhibition of HIF-1α using YC-1 significantly suppressed radiation-induced Ephb4 expression (Fig. S4B), suggesting that hypoxia-associated signaling contributes to the regulation of Ephb4 expression following IR. Consistent with these in vitro findings, immunohistochemical analysis confirmed increased Ephb4 protein expression in irradiated MC38 tumors in vivo (Fig. 2F). As Ephb4 functions through interaction with its ligand ephrinb2 [38], we next examined the spatial relationship between Ephb4-expressing tumor cells and ephrinb2-expressing macrophages within the irradiated TME. Ephb4-expressing tumor cells were surrounded by infiltrating macrophages, with ephrinb2-expressing macrophages localized in close proximity to, or in direct contact with, tumor cells (Fig. S5).

To explore the role of Ephb4 in tumor–macrophage interactions, we generated *Ephb4*-KO tumor cells using CRISPR/Cas9 (Fig. S6A). In a 3D microfluidic coculture system, irradiated WT tumor cells maintained close physical interactions with macrophages, whereas *Ephb4*-KO cells exhibited significantly reduced contact (Fig. 2G). WT and *Ephb4**-***KO tumor cells exhibited comparable radiosensitivity in monoculture (Fig. S6B and C). In contrast, in the presence of macrophages, WT tumor cells maintained survival and proliferation at levels comparable to nonirradiated conditions following IR, whereas *Ephb4**-***KO cells showed markedly increased cell death and impaired proliferation (Fig. 2H and I). We next examined whether this protective effect required direct cell–cell contact. We compared direct coculture conditions with Transwell systems that permit paracrine signaling but prevent physical contact (Fig. 2J). Notably, physical separation attenuated the differential radiation response between WT and *Ephb4*-KO tumor cells observed under macrophage coculture conditions following IR (Fig. 2K). These findings suggest that radiation induces Ephb4 expression in tumor cells, enabling contact-dependent crosstalk with macrophages to promote tumor cell survival.

### Ephb4 deficiency in tumor cells enhanced radiosensitivity in vivo

**Fig. 3.**
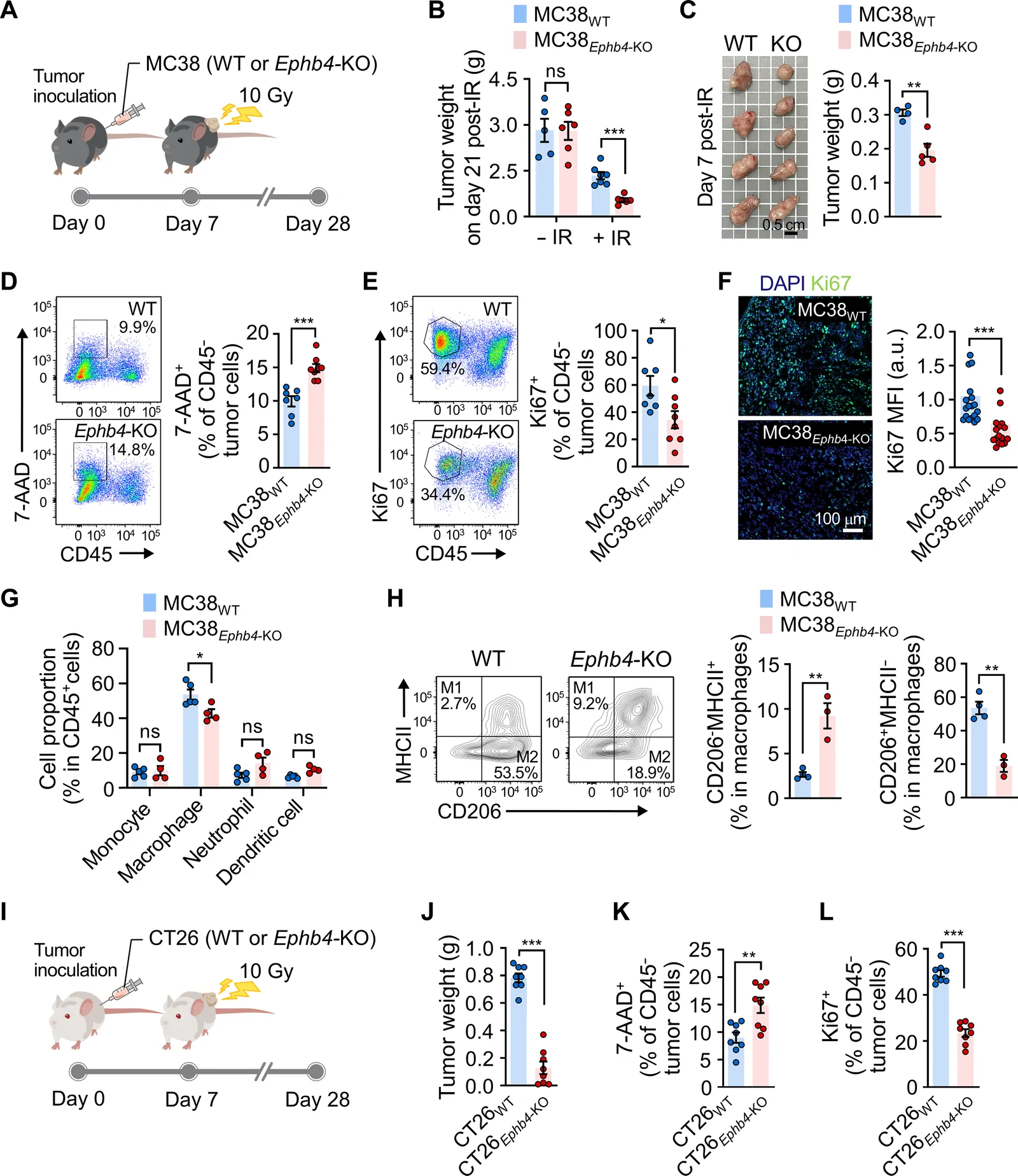
Ephrin receptor b4 (*Ephb4*) deficiency enhances tumor radiosensitivity in vivo. (A) Schematic illustration of the in vivo CRC mouse model. Wild-type (WT) or *Ephb4*-knockout (KO) MC38 tumor cells were subcutaneously implanted into C57BL/6 mice, followed by 10-Gy irradiation (IR) on day 7. (B) Tumor weight of WT and *Ephb4*-KO MC38 mouse model under nonirradiated (*n* = 5 to 6 per group) and irradiated conditions (21 d post-IR) (*n* = 6 to 7 per group). Data were pooled from 2 independent experiments. (C) Representative images of tumor (left) and quantification of the tumor weight (right) of MC38 WT (*n* = 4) and *Ephb4*-KO (*n* = 5) tumors 7 d post-IR. (D and E) Percentage of 7-aminoactinomycin D (7-AAD)^+^ dead cells (D), and Ki67^+^ cells (E) in the CD45^−^ tumor cell-enriched population derived from MC38 WT (*n* = 7) and *Ephb4*-KO (*n* = 8) tumor on day 7 post-IR. (F) Representative immunofluorescence (IF) images of Ki67 (green) and 4′,6-diamidino-2-phenylindole (DAPI) (blue) in tumor tissues from WT and *Ephb4*-KO MC38 mouse models (left) and the quantification of Ki67 mean fluorescence intensity (MFI) were shown on the right. (G) Flow cytometric analysis of tumor-infiltrating immune cell populations in WT (*n* = 5) and *Ephb4*-KO (*n* = 4) MC38 tumors 7 d post-IR. (H) Representative flow cytometry plots and percentage of M1-like (CD206^−^MHCII^+^) and M2-like (CD206^+^MHCII^−^) macrophage subsets in irradiated WT (*n* = 4) and *Ephb4*-KO (*n* = 5) tumors. (I) Schematic illustration of the CT26 tumor model. WT or *Ephb4*-KO CT26 tumor cells were subcutaneously implanted into BALB/c mice, followed by IR (10 Gy) on day 7. (J to L) Tumor weight (J), and percentage of 7-AAD^+^ dead cells (K), and Ki67^+^ cells (L) in the CD45^−^ tumor cell-enriched population derived from CT26 WT or *Ephb4*-KO tumor on 7 d after IR (*n* = 8 per group). Data are presented as means ± standard error of the mean (SEM) (B to H and J to L). ns, not significant, *P < 0.05, **P < 0.01, ***P < 0.001 by unpaired 2-tailed Student’s *t* test (B to F, H, and J to L) or 2-way analysis of variance (ANOVA) (G). a.u., arbitrary units; MHCII, major histocompatibility complex class II.

To evaluate whether Ephb4-mediated tumor cell–macrophage crosstalk contributes to tumor radiosensitivity, we used a syngeneic colorectal tumor model implanted with either WT or *Ephb4*-KO MC38 tumor cells (Fig. 3A). Without IR, tumor growth was comparable between groups. However, following IR, *Ephb4-*KO tumors exhibited significant reduction in tumor weight than WT controls, with differences emerging as early as day 7 and persisting through day 21 (Fig. 3B and C). Therefore, day 7 was selected for subsequent mechanistic analyses. Consistent with the increased tumor radiosensitivity, *Ephb4-*KO tumors exhibited increased cell death and decreased proliferation compared with WT controls (Fig. 3D to F).

We next examined immune alterations associated with *Ephb4* loss. The infiltration levels of monocyte, neutrophil, and dendritic cell remained unchanged, whereas macrophage infiltration was significantly reduced in *Ephb4-*KO tumors compared with WT controls (Fig. 3G). Tumor-infiltrating macrophages also displayed a shift toward a proinflammatory M1-like phenotype in *Ephb4-*KO tumors, characterized by a significantly increased proportion of CD206^−^MHCII^+^ cells and a significantly decreased proportion of immunosuppressive CD206^+^MHCII^−^ M2-like macrophages compared with WT controls (Fig. 3H). This phenotypic reprogramming was associated with significantly decreased expression of M2 macrophage-associated genes, such as mannose receptor c-type 1 (*Mrc1*) and arginase 1 (*Arg1*), in irradiated *Ephb4-*KO tumors compared to WT tumors, while vascular endothelial growth factor a (*Vegfa*) levels remained unchanged (Fig. S7A).

Lymphoid profiling further revealed enhanced antitumor immunity in *Ephb4-*KO tumors, marked by significantly increased infiltration of CD8^+^ T cells compared to WT tumors (Fig. S7B). Notably, CD8^+^ T cells in *Ephb4-*KO tumors also exhibited enhanced effector activity relative to those in WT tumors, as demonstrated by increased expression of IFN-γ and granzyme B (Fig. S7C and D). To determine whether the differential tumor radiosensitivity between WT and *Ephb4-*KO tumors was dependent on CD8^+^ T cells, CD8^+^ T cell depletion experiments were performed. Despite efficient depletion (Fig. S7E), the difference in tumor response to IR was preserved, with *Ephb4-*KO tumors remaining significantly smaller than WT tumors (Fig. S7F), indicating that CD8^+^ T cells were not the primary drivers of the enhanced tumor radiosensitivity.

These findings were also observed in the CT26 colorectal tumor model (Fig. 3I). Compared to WT controls, *Ephb4*-deficient CT26 tumors similarly exhibited enhanced radiosensitivity, characterized by significantly reduced tumor weight, increased tumor cell death, and decreased Ki67 expression following IR (Fig. 3J to L).

These results demonstrate that *Ephb4* deficiency enhances tumor radiosensitivity in a microenvironment-dependent manner and is associated with reduced macrophage infiltration, macrophage phenotypic reprogramming, and increased tumor cell death following IR.

### Macrophage-induced juxtacrine activation of Ephb4 protected tumor cells from radiation-induced ferroptosis

**Fig. 4.**
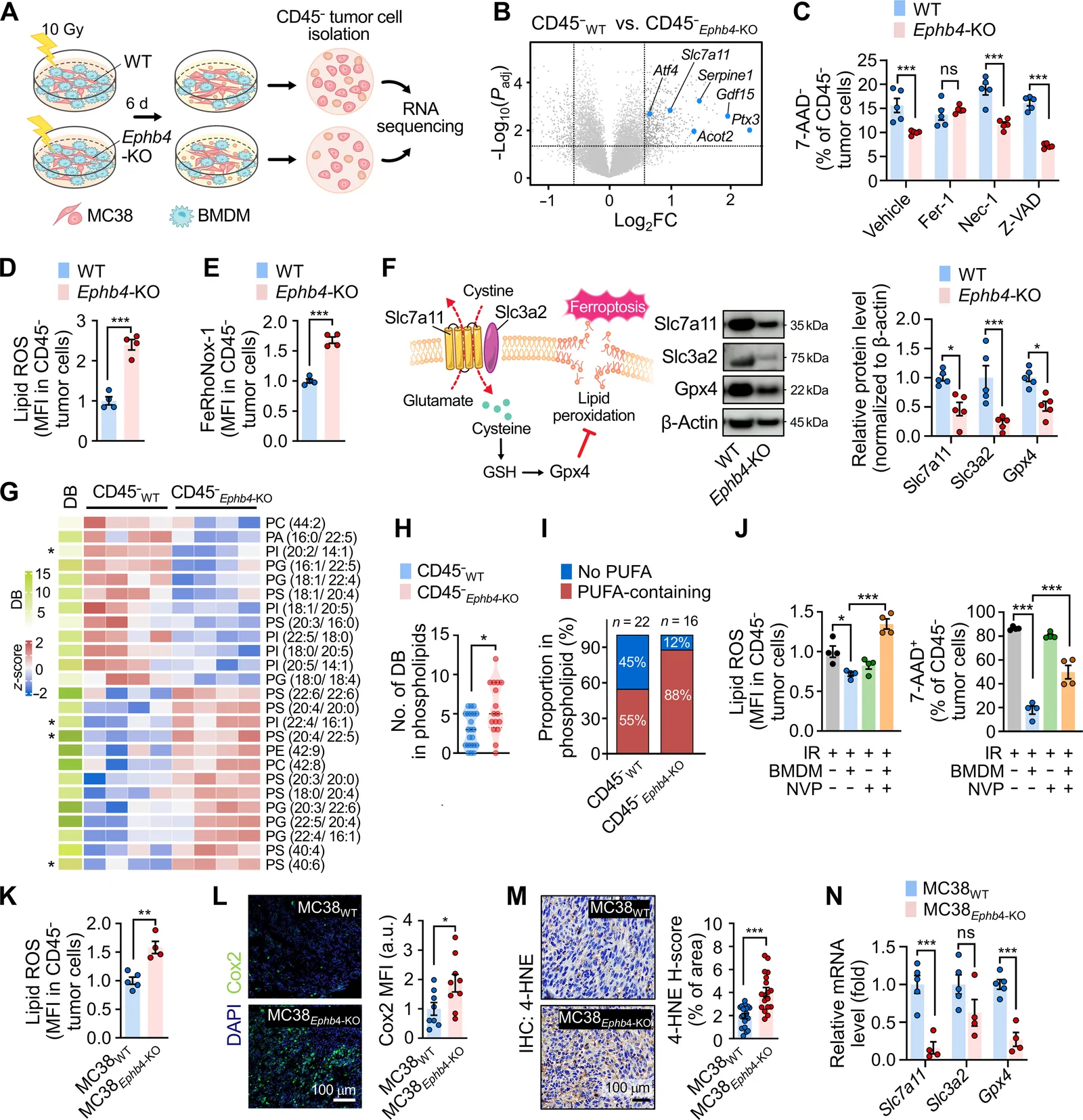
Ephrin receptor b4 (Ephb4) conferred irradiation (IR) resistance by suppressing tumor cell ferroptosis. (A) Schematic illustration of the RNA sequencing experimental setup. Wild-type (WT) or *Ephb4-*knockout (KO) MC38 cells were cocultured with bone marrow-derived macrophages (BMDMs) for 6 d following IR. Subsequently, CD45^−^ tumor cells were isolated from the coculture using anti-mouse CD45 magnetic microbeads, and isolated WT or *Ephb4-*KO MC38 cells were subjected to transcriptomic profiling. (B) Volcano plot showing the differentially expressed gene profiles of isolated WT vs. *Ephb4*-KO CD45^−^ tumor cells (*n* = 3 per group). (C) Percentage of viable tumor cells (7-AAD^−^CD45^−^) in WT or *Ephb4*-KO tumor cells at 4 d post-IR under BMDM coculture conditions. Cells were treated with vehicle or the indicated inhibitors: ferrostatin-1 (Fer-1, 10 μmol/L), necrostatin-1 (Nec-1, 10 μmol/L), or Z-VAD-FMK (Z-VAD, 5 μmol/L), *n* = 5 per group. (D) Relative lipid reactive oxygen species (ROS) in WT and *Ephb4*-KO tumor cells (CD45^−^) at 3 d post-IR under BMDM coculture conditions (*n* = 5 per group). (E) Intracellular iron levels measured using FeRhoNox-1 in WT and *Ephb4*-KO tumor cells (CD45^−^) at 3 d post-IR under BMDM coculture conditions (*n* = 4 per group). (F) Schematic illustration of established antiferroptotic mechanisms adapted from prior studies [77] (left), in which the system χ_c_^−^ transporter (solute carrier family 7 member 11 [Slc7a11]/solute carrier family 3 member 2 [Slc3a2]) imports cystine to support glutathione synthesis and glutathione peroxidase 4 (Gpx4)-mediated detoxification of lipid peroxides, thereby suppressing ferroptosis. Immunoblotting analysis of Slc7a11, Slc3a2, and Gpx4 expression in WT and *Ephb4*-KO tumor cells at 3 d post-IR under BMDM coculture conditions, following magnetic isolation using anti-CD45 microbeads, as shown in (A). Right, quantification of relative protein levels normalized to β-actin (*n* = 5 per group). (G) WT or *Ephb4-*KO MC38 cells were cocultured with BMDMs for 3 d post-IR, followed by CD45-based magnetic isolation and lipidomic profiling. Focused heatmap of significantly altered phospholipids. Asterisks (*) indicate phospholipid species that are significantly different between WT and *Ephb4-*KO groups based on the Wilcoxon rank-sum test (*P* < 0.01). (H) Total unsaturation burden per sample. Violin plot showing the summed number of double bonds across all quantified phospholipid species in WT or *Ephb4-*KO MC38 cells isolated 3 d post-IR under BMDM coculture. (I) Proportion of polyunsaturated fatty acyl chain (PUFA)-containing phospholipid species (≥2 double bonds) in WT or *Ephb4-*KO MC38 cells isolated 3 d post-IR under BMDM coculture. Bar graph represents the proportion of PUFA-containing and non-PUFA-containing species among the detected phospholipids, with a total of 22 species in WT and 16 species in *Ephb4-*KO cells. (J) Relative lipid ROS levels (left) and percentage of 7-aminoactinomycin D (7-AAD)^+^ dead cells (right) in WT tumor cells (CD45^−^) cultured alone or cocultured with BMDMs, treated with vehicle or the Ephb4 inhibitor NVP-BHG712 (NVP) (1 μmol/L) prior to IR (*n* = 4 per group). Lipid ROS was measured 3 d post-IR, and cell death was assessed 4 d post-IR. (K) Relative lipid ROS levels in CD45^−^ tumor cell-enriched populations from WT (*n* = 5) and *Ephb4*-KO (*n* = 4) MC38 tumors on day 7 post-IR. (L) Representative immunofluorescence (IF) images of cyclooxygenase 2 (Cox2) (green) and 4′,6-diamidino-2-phenylindole (DAPI) (blue) in WT (*n* = 5) and *Ephb4*-KO (*n* = 4) MC38 tumors on day 7 post-IR (10 Gy, left), with quantification of Cox2 mean fluorescence intensity (MFI) shown on the right. For quantification, 1 to 3 fields per tumor section were analyzed. (M) Representative immunohistochemistry (IHC) images (left) and quantification (right) of 4-hydroxynonenal (4-HNE) in WT (*n* = 5) and *Ephb4*-KO (*n* = 4) MC38 tumors 7 d post-IR (10 Gy). For quantification, 3 to 5 fields per tumor section were analyzed. (N) Relative mRNA levels of Slc7a11, Slc3a2, and Gpx4 in WT and *Ephb4*-KO MC38 tumors 7 d post-IR (10 Gy) (*n* = 4 per group for *Slc7a11* and *Gpx4; n* = 5 for *Slc3a2*). Data are presented as means ± standard error of the mean (SEM) (C to F, H, and J to N). ns, not significant, *P < 0.05, **P < 0.01, ***P < 0.001 by unpaired 2-tailed Student’s *t* test (D and E and K to M), 1-way analysis of variance (ANOVA) with Tukey’s multiple comparison test (J), Wilcoxon rank-sum test (G and H), or 2-way ANOVA (C, F, and N). a.u., arbitrary units; DB, double bond; FC, fold change; GSH, glutathione; PA, phosphatidic acid; PC, phosphatidylcholine; PE, phosphatidylethanolamine; PG, phosphatidylglycerol; PI, phosphatidylinositol; PS, phosphatidylserine.

To investigate how macrophage contact promotes tumor cell resistance to radiation via Ephb4 signaling, we performed bulk RNA-seq on isolated WT and *Ephb4*-KO tumor cells after coculturing with BMDMs and IR (Fig. 4A). Transcriptomic analysis revealed decreased expression of ferroptosis-inhibitory genes, including *Slc7a11* [41,42], *growth differentiation factor 15* (*Gdf15*) [43], and pentraxin 3 (*Ptx3*) [44], in isolated *Ephb4-*KO tumor cells compared to those in WT tumor cells (Fig. 4B), suggesting that Ephb4 may be involved in the regulation of ferroptosis in tumor cells following IR.

We next examined whether macrophages influence ferroptotic stress in tumor cells after IR. Measurement of lipid ROS, a key indicator of ferroptosis [45], showed that tumor cells cocultured with macrophages exhibited significantly reduced lipid ROS levels following IR, compared with tumor cells cultured alone. This reduction in lipid ROS was significantly attenuated when tumor cells and macrophages were separated using a Transwell system, indicating a contact-dependent mechanism (Fig. S8A). Moreover, treatment with the ferroptosis inhibitor ferrostatin-1 eliminated the difference in lipid ROS levels between cocultured and monocultured tumor cells (Fig. S8B).

To further clarify whether the differential survival of WT and *Ephb4*-deficient tumor cells under macrophage coculture conditions was attributable to ferroptosis rather than other forms of cell death, we treated cocultures with inhibitors targeting ferroptosis (ferrostatin-1), necroptosis (necrostatin-1), or apoptosis (Z-VAD-FMK). Only ferrostatin-1 abolished the survival difference between WT and *Ephb4*-deficient tumor cells (Fig. 4C), indicating that ferroptosis is the primary mode of cell death underlying the differential survival observed under macrophage coculture conditions. Consistently, *Ephb4-*deficient tumor cells accumulated significantly higher levels of lipid ROS and intracellular iron following IR in macrophage coculture compared with WT cells (Fig. 4D and E and Fig. S8C and D). To further assess the functional impact of Ephb4 activation on ferroptotic stress, we treated irradiated WT tumor cells with recombinant ephrinb2-Fc. This treatment reduced lipid ROS levels and protected tumor cells from radiation-induced death (Fig. S8E and F).

Analysis of the RNA-seq dataset further revealed that the transcription factor Atf4, a known regulator of ferroptosis resistance [46], was prominently down-regulated in *Ephb4*-KO tumor cells compared to WT tumor cells following IR under macrophage coculture conditions (Fig. 4B). Consistently, Atf4 expression was significantly higher in isolated WT tumor cells than in *Ephb4*-deficient cells following macrophage coculture and IR (Fig. S8G), and was further increased upon activation of Ephb4 signaling by ephrinb2-Fc treatment (Fig. S8H). In line with the role of Atf4 in regulating ferroptosis-protective genes, expression of canonical antiferroptotic genes [46], including *Slc7a11*, *Slc3a2*, and *Gpx4*, was significantly higher in irradiated WT tumor cells than in *Ephb4*-deficient cells, as confirmed by immunoblotting and quantitative PCR (Fig. 4F and Fig. S8I).

We next asked whether Ephb4 signaling alters lipid composition associated with ferroptosis susceptibility. Targeted lipidomics using liquid chromatography-mass spectrometry (LC-MS) was performed on irradiated tumor cells isolated after macrophage coculture (Fig. S9A to C). Lipidomic profiling revealed that *Ephb4*-KO tumor cells exhibited a lipid composition consistent with increased susceptibility to ferroptosis, characterized by a marked enrichment of polyunsaturated phospholipid species containing a high number of double bonds (>4) [47–49] (Fig. 4G to I and Fig. S9D). Several ferroptosis-associated phospholipids [48,49], including PS (20:4/22:5) and PS (40:6), were significantly increased in *Ephb4*-deficient cells (Fig. 4G and Fig. S9E). Pharmacological inhibition of Ephb4 with NVP-BHG712 recapitulated the *Ephb4*-deficient phenotype, resulting in significantly increased lipid ROS and enhanced radiation-induced cell death (Fig. 4J).

We next validated these findings in vivo. In both MC38 and CT26 tumor models, *Ephb4*-deficient tumors exhibited significantly elevated lipid peroxidation in tumor cell-enriched populations following IR (Fig. 4K and Fig. S10). *Ephb4*-KO tumors also showed significantly higher expression of ferroptosis markers, including cyclooxygenase 2 (Cox2) and 4-hydroxynonenal, along with reduced expression of *Slc7a11*, *Slc3a2*, and *Gpx4* compared to WT tumors following IR (Fig. 4L to N).

Collectively, these findings indicated that Ephb4-mediated macrophage–tumor cell contact promoted a protective transcriptional and metabolic program that suppressed ferroptosis and enhanced resistance to radiation.

### Repeated IR in the presence of macrophages drove the acquisition of stable radioresistance through Ephb4-dependent ferroptosis evasion

**Fig. 5.**
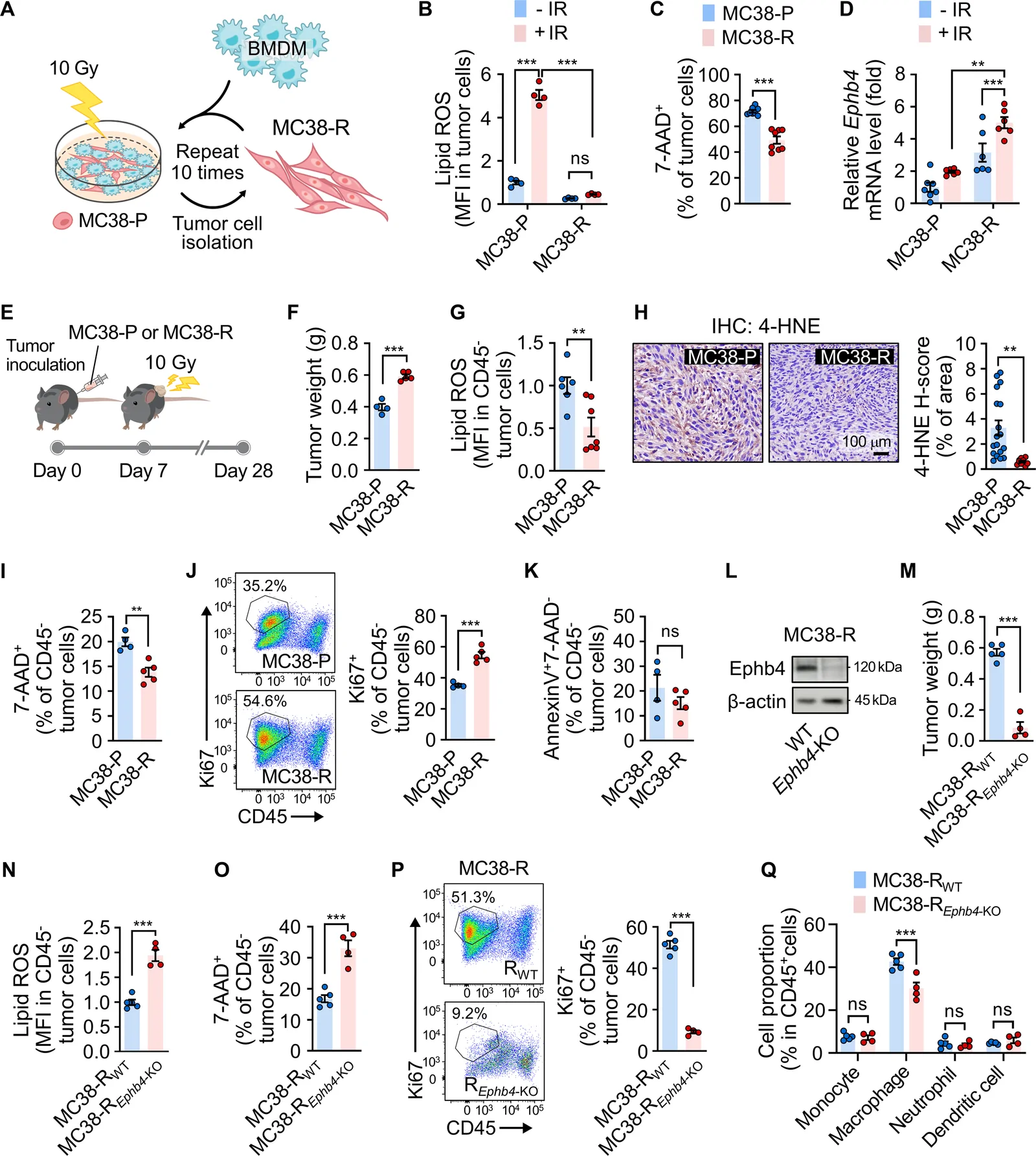
Macrophage-induced Ephrin receptor b4 (Ephb4) signaling in tumor cells promoted adaptive radioresistance through ferroptosis evasion. (A) Schematic illustration of the in vitro model for generating macrophage-mediated radioresistant tumor cells. Parental MC38 (MC38-P) tumor cells were cocultured with bone marrow-derived macrophages (BMDMs) and subjected to 10 rounds of 10-Gy irradiation (IR), followed by isolation of radioresistant tumor cells (MC38-R). (B) Relative lipid reactive oxygen species (ROS) in parental and radioresistant tumor cells at 3 d with or without IR (10 Gy; *n* = 4 per group). (C) Percentage of 7-aminoactinomycin D (7-AAD)^+^ dead cells (right) in parental and radioresistant tumor cells at 4 d post-IR (*n* = 8 per group). (D) Relative *Ephb4* mRNA levels in parental and radioresistant tumor cells with (*n* = 6 per group) or without IR (*n* = 7 for MC38-P and *n* = 6 for MC38-R). (E) Schematic illustration of the in vivo mouse model. C57BL/6 mice were implanted with MC38-P or MC38-R tumor cells and treated with IR on day 7. (F and G) Tumor weight (7 d post-IR, *n* = 4 for MC38-P; *n* = 5 for MC38-R; F) and relative lipid ROS (21 d post-IR, *n* = 6 for MC38-P; *n* = 7 for MC38-R; G) in tumors derived from MC38-P and MC38-R mouse models. Data presented in (G) have been pooled from 2 independent experiments. (H) Representative immunohistochemistry (IHC) images (left) and quantification (right) of 4-hydroxynonenal (4-HNE) staining in tumors derived from MC38-P and MC38-R mouse models. (I-K) Percentage of 7-AAD^+^ dead cells (I), Ki67^+^ cells (J), and AnnexinV^+^7-AAD^−^ apoptotic cells (K) in CD45^−^ tumor cell-enriched populations from the tumor of MC38-P (*n* = 4) and MC38-R (*n* = 5) mouse models 7 d post-IR. (L) Immunoblotting analysis confirming Ephb4 protein expression in MC38-R cells (wild-type [WT] vs. *Ephb4-*knockout [KO]). (M) Weight of tumors derived from mice implanted with WT MC38-R cells (MC38-R_WT_) and *Ephb4*-KO MC38-R cells (MC38-R_*Ephb4-*KO_) 7 d post-IR (*n* = 4 to 5 per group). (N) Relative lipid ROS in CD45^−^ tumor cell-enriched populations from WT (*n* = 5) and *Ephb4*-KO (*n* = 4) MC38-R tumors. (O and P) Percentage of 7-AAD^+^ dead cells (O) and Ki67^+^ cells (P) in CD45^−^ tumor cell-enriched populations from WT (*n* = 5) and *Ephb4*-KO (*n* = 4) MC38-R tumors. (Q) Flow cytometric analysis of tumor-infiltrating immune populations in MC38-R_WT_ (*n* = 5) vs. MC38-R_*Ephb4*-KO_ (*n* = 4) tumors. All IR experiments were performed using a single dose of 10 Gy. Data are presented as means ± standard error of the mean (SEM) (B to D, F to K, M to Q). ns, not significant, **P < 0.01, ***P < 0.001 by 2-way analysis of variance (ANOVA) (B, D, and Q) or unpaired 2-tailed Student’s *t* test (C and F to P).

We next explored whether prolonged exposure to a macrophage-rich environment could enable tumor cells to acquire stable resistance to radiation-induced cell death. To model this process, we subjected tumor cells to 10 consecutive cycles of macrophage coculture and IR, generating a stably radioresistant subpopulation (Fig. 5A). Compared to parental tumor cells, radioresistant tumor cells exhibited significantly reduced lipid peroxidation and cell death after IR (Fig. 5B and C). Notably, *Ephb4* mRNA expression was significantly up-regulated in radioresistant tumor cells relative to parental tumor cells following IR (Fig. 5D). Importantly, parallel tumor cells exposed to repeated IR in the absence of macrophages developed only modest resistance and failed to exhibit comparable suppression of lipid peroxidation, compared to tumor cells that acquired radioresistance through macrophage coculture (Fig. S11), indicating that stable ferroptosis resistance is predominantly driven by macrophage-dependent conditioning rather than IR alone.

We then assessed whether this macrophage-mediated radioresistance phenotype persisted in vivo. Upon implantation into syngeneic mice and subsequent IR (Fig. 5E), radioresistant tumors displayed significantly greater tumor burden (Fig. 5F), lower lipid ROS levels (Fig. 5G), and reduced 4-hydroxynonenal staining (Fig. 5H), consistent with ferroptosis evasion. Radioresistant tumors also exhibited significantly reduced cell death and increased Ki67^+^ tumor cells (Fig. 5I and J), without significant changes in apoptosis (Fig. 5K).

To determine whether Ephb4 is required to maintain this stable resistance, we generated *Ephb4*-KO radioresistant cells (Fig. 5L) and evaluated their behavior in vivo. Loss of *Ephb4* resensitized radioresistant tumors to radiation, as evidenced by significantly reduced tumor weight (Fig. 5M), elevated lipid peroxidation (Fig. 5N), increased cell death (Fig. 5O), and decreased proliferation (Fig. 5P) compared with radioresistant control tumors. Furthermore, *Ephb4*-KO radioresistant tumors showed reduced infiltration of TAMs compared to WT radioresistant tumors (Fig. 5Q).

Collectively, these findings demonstrated that macrophage presence during IR facilitated the acquisition of a radioresistant phenotype in tumor cells, in association with Ephb4-mediated suppression of ferroptosis.

### Ephrinb2 reverse signaling activated the Tlr2–NF-κB axis in macrophages, driving IL-6 secretion and reinforcing paracrine ferroptosis resistance

**Fig. 6.**
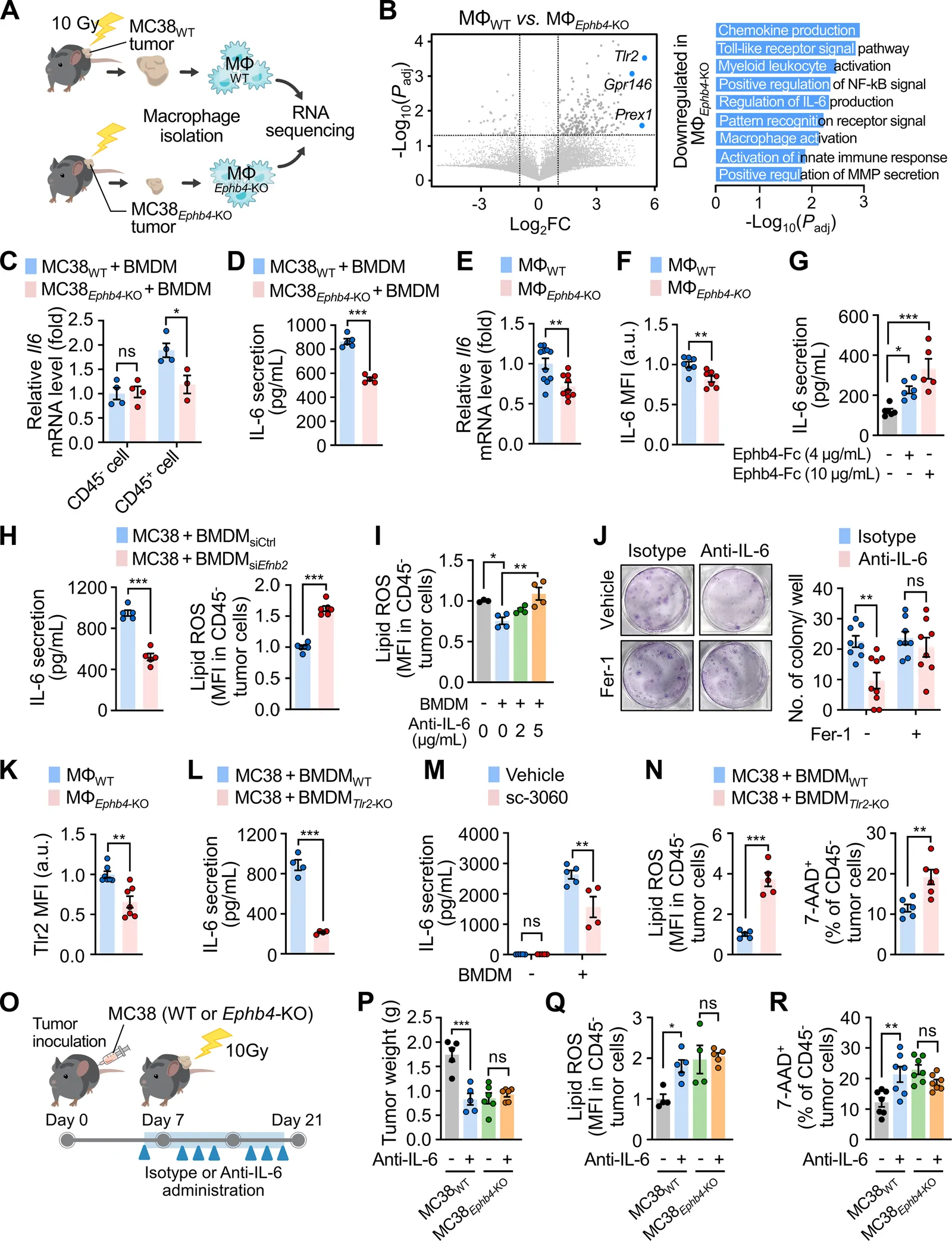
Ephrinb2 reverse signaling activated the toll-like receptor 2 (Tlr2)-nuclear factor-kappa B (NF-κB) axis in macrophages, driving interleukin-6 (IL-6) secretion and reinforcing paracrine ferroptosis resistance. (A) Schematic illustration of RNA sequencing (RNA-seq) experimental setup. MC38 wild-type (WT) or Ephrin receptor b4 (*Ephb4*)-knockout (KO) tumor cells were subcutaneously implanted into C57BL/6 mice, followed by irradiation (IR) on day 7. Tumor-infiltrating macrophages were isolated by flow cytometry at day 21 post-IR for RNA-seq. (B) Volcano plot (left) and pathway enrichment analysis (right) comparing gene expression profiles in macrophages from WT versus *Ephb4*-KO tumors. Blue dots indicate the top down-regulated genes in macrophages isolated from *Ephb4*-KO tumors compared to WT tumors. (C) *Il6 mRNA* levels in CD45^−^ tumor cells and CD45^+^ bone marrow-derived macrophages (BMDMs) following 6 d of coculture between MC38 tumor cells (WT or *Ephb4*-KO) and BMDMs after IR. Tumor cells and macrophages were separated using anti-CD45 magnetic microbeads prior to analysis (*n* = 3 to 4 per group). (D) IL-6 secretion measured by enzyme-linked immunosorbent assay (ELISA) in supernatants from cocultures of WT or *Ephb4*-KO MC38 cells with BMDMs, 2 d post-IR (*n* = 5 per group). (E) *Il6* mRNA levels in tumor-infiltrating macrophages isolated by fluorescence-activated cell sorting (FACS) from WT (*n* = 11) or *Ephb4*-KO (*n* = 9) MC38 tumors. (F) Mean fluorescence intensity (MFI) of IL-6 in tumor-infiltrating macrophages from WT or *Ephb4*-KO tumors, analyzed on day 21 post-IR (*n* = 7 per group). (G) IL-6 secretion measured by ELISA in supernatants from BMDMs treated with recombinant Ephb4-Fc at 4 μg/mL (*n* = 6) or 10 μg/mL (*n* = 5) 2 d post-IR. (H) IL-6 secretion (left) and relative lipid reactive oxygen species (ROS) in CD45^−^ tumor cells (right) in cocultures of MC38 tumor cells with BMDMs transfected with control or *Efnb2* small interfering RNA (siRNA) following IR (*n* = 5 per group). IL-6 levels were measured in coculture supernatants at 2 d post-IR, and lipid ROS was assessed in CD45^−^ tumor cells at 3 d post-IR. (I) Relative lipid ROS in MC38 tumor cells cocultured with BMDMs treated with anti-IL-6 neutralizing antibody (2 or 5 μg/mL) (*n* = 3 to 4 per group). (J) Representative images (left) and quantification (right) of colony formation in MC38 tumor cells in coculture with BMDMs post-IR. Following IR, cocultures were treated with isotype control (*n* = 8), anti-IL-6 (10 μg/mL, *n* = 9), Fer-1 (10 μmol/L, *n* = 8), or Fer-1 + anti-IL-6 (*n* = 8). Colonies were quantified 10 d post-IR. Data were pooled from 3 independent experiments. (K) MFI of Tlr2 in tumor-infiltrating macrophages from WT or *Ephb4*-KO tumors, analyzed on day 21 post-IR (*n* = 7 per group). (L) IL-6 secretion in cocultures of MC38 tumor cells with WT or *Tlr2*-KO BMDMs, measured 2 d post-IR. (*n* = 4 per group). (M) IL-6 secretion measured by ELISA in supernatants from MC38 tumor cells cultured alone or cocultured with BMDMs, with or without NF-κB inhibitor sc-3060 treatment (10 μmol/L), 2 d post-IR (*n* = 4 to 5 per group). (N) Relative lipid ROS (left, *n* = 5 per group) and percentage of 7-aminoactinomycin D (7-AAD)^+^ cell death (right, *n* = 6 per group) in CD45^−^-gated MC38 tumor cells directly analyzed by flow cytometry under coculture conditions with WT or *Tlr2*-KO BMDMs. Lipid ROS and cell death were assessed by flow cytometry at 3 and 4 d post-IR, respectively. (O) Schematic of the in vivo experimental design. WT or *Ephb4*-KO MC38 tumor-bearing mice were irradiated and treated with either isotype control or anti-IL-6 antibody (300 μg). (P to R) Tumor weight (P, *n* = 5 to 6 per group), relative lipid ROS (Q, *n* = 4 to 5 per group), and percentage of 7-AAD^+^ cell death (R, *n* = 7 per group) in CD45^−^ tumor cell-enriched populations from WT or *Ephb4*-KO MC38 tumors with or without anti-IL-6 treatment, measured 14 d post-IR. All IR experiments were performed using a single dose of 10 Gy. Data are presented as means ± standard error of the mean (SEM) (C to N, P to R). ns, not significant, *P < 0.05, **P < 0.01, ***P < 0.001 by unpaired 2-tailed Student’s *t* test (D to F, H, K and L, and N), 1-way analysis of variance (ANOVA) with Tukey’s multiple comparison test (G, I, and P to R) or 2-way ANOVA (C, J, and M). Ctrl, FC, fold change; MΦ, macrophage.

Ephb4–ephrinb2 signaling is a canonical bidirectional pathway activated by cell–cell contact, wherein Ephb4-expressing cells initiate forward signaling and ephrinb2-expressing cells engage in reverse signaling [38]. Thus, we hypothesized that radiation-induced tumor cell–macrophage interactions not only activate Ephb4 in tumor cells but also trigger ephrinb2 reverse signaling in macrophages. To test this, we performed bulk RNA-seq on fluorescence-activated cell sorting-sorted macrophages from tumors of mice bearing either WT or *Ephb4*-KO cells, following tumor IR (Fig. 6A). Macrophages from *Ephb4*-KO tumors showed decreased expression of genes associated with Toll-like receptor signaling, NF-κB signaling, and IL-6 production compared to those from WT tumors (Fig. 6B). Among these findings, the up-regulation of IL-6 was particularly notable, given its reported roles in suppressing ferroptosis [50] and mitigating oxidative stress in irradiated tumor cells [51]. These observations led us to hypothesize that the down-regulated IL-6 production observed in macrophages from *Ephb4*-KO tumors is driven by ephrinb2-mediated reverse signaling and functionally contributes to macrophage-dependent suppression of ferroptosis in tumor cells.

Supporting this hypothesis, *Il6* mRNA levels remained unchanged in tumor cells but were significantly reduced in macrophages cocultured with *Ephb4*-KO tumor cells compared to those cocultured with WT tumor cells (Fig. 6C). This reduction was also evident at the protein level in coculture supernatants (Fig. 6D). Importantly, macrophages isolated in vivo from *Ephb4*-KO tumors exhibited significantly lower *Il6* mRNA levels than those from WT tumors (Fig. 6E), and flow cytometric analysis confirmed decreased IL-6 production (Fig. 6F), further validating a potential role for Ephb4-dependent macrophage reverse signaling in regulating IL-6 production in vivo. Moreover, stimulation of macrophages with recombinant Ephb4-Fc in the absence of tumor cells induced IL-6 secretion (Fig. 6G), whereas treatment of tumor cells with recombinant ephrinb2-Fc failed to induce IL-6 expression (Fig. S12A). To directly examine the importance of ephrinb2 reverse signaling, we silenced *Efnb2* in macrophages. *Efnb2* knockdown significantly reduced IL-6 production and increased lipid peroxidation in irradiated tumor cells (Fig. 6H), implicating ephrinb2 reverse signaling in macrophage-mediated ferroptosis protection. Similarly, IL-6 neutralization increased lipid ROS accumulation and impaired clonogenic survival of irradiated tumor cells, while Ferrostatin-1 treatment partially rescued the reduced clonogenic survival (Fig. 6I and J).

We next sought to identify the signaling cascade linking ephrinb2 activation to IL-6 induction. RNA-seq data revealed *Tlr2* as one of the most strongly down-regulated genes in macrophages from *Ephb4*-KO tumors (Fig. 6B), with enrichment of toll-like receptor and NF-κB pathways. Consistently, flow cytometric analysis of tumor-infiltrating macrophages demonstrated higher Tlr2 expression in macrophages from WT tumors compared to those from *Ephb4*-KO tumors (Fig. 6K). Considering the established role of the Tlr2–NF-κB pathway in regulating IL-6 expression [52,53], we tested whether ephrinb2 activation directly engages this pathway in macrophages. Ephb4-Fc-mediated stimulation of ephrinb2 increased Tlr2 expression and activated NF-κB signaling in macrophages (Fig. S12B). Moreover, IL-6 secretion was markedly reduced in *Tlr2*-deficient macrophages and in macrophages treated with an NF-κB inhibitor (Fig. 6L and M). Functionally, *Tlr2*-deficient macrophages failed to suppress lipid peroxidation and were unable to protect tumor cells from radiation-induced death (Fig. 6N).

To validate these findings in vivo, we treated irradiated tumor-bearing mice with anti-IL-6 antibodies (Fig. 6O). IL-6 blockade sensitized WT tumors to radiation, as compared with tumors treated with isotype control antibodies, leading to reduced tumor growth (Fig. 6P), increased lipid peroxidation (Fig. 6Q), and elevated tumor cell death (Fig. 6R). These effects were not observed in *Ephb4*-KO tumors. Mechanistically, IL-6 neutralization also decreased the expression of key ferroptosis-protective genes (*Slc7a11*, *Slc3a2*, and *Gpx4*) (Fig. S12C). Importantly, cotreatment with the ferroptosis inhibitor liproxstatin-1 attenuated the tumor-suppressive effect of IL-6 blockade (Fig. S12D), indicating that the therapeutic benefit of IL-6 inhibition is mediated through enhanced ferroptosis.

Collectively, these findings delineate a bidirectional signaling axis in which Ephb4 activation in tumor cells promotes intrinsic ferroptosis resistance, while ephrinb2 signaling in macrophages triggers IL-6 production through the Tlr2–NF-κB pathway (Fig. S12E). This IL-6-driven paracrine loop further reinforces ferroptosis evasion in irradiated tumors.

### Pharmacological Ephb4 inhibition restored ferroptosis sensitivity by disrupting both tumor-intrinsic and macrophage-mediated resistance programs

**Fig. 7.**
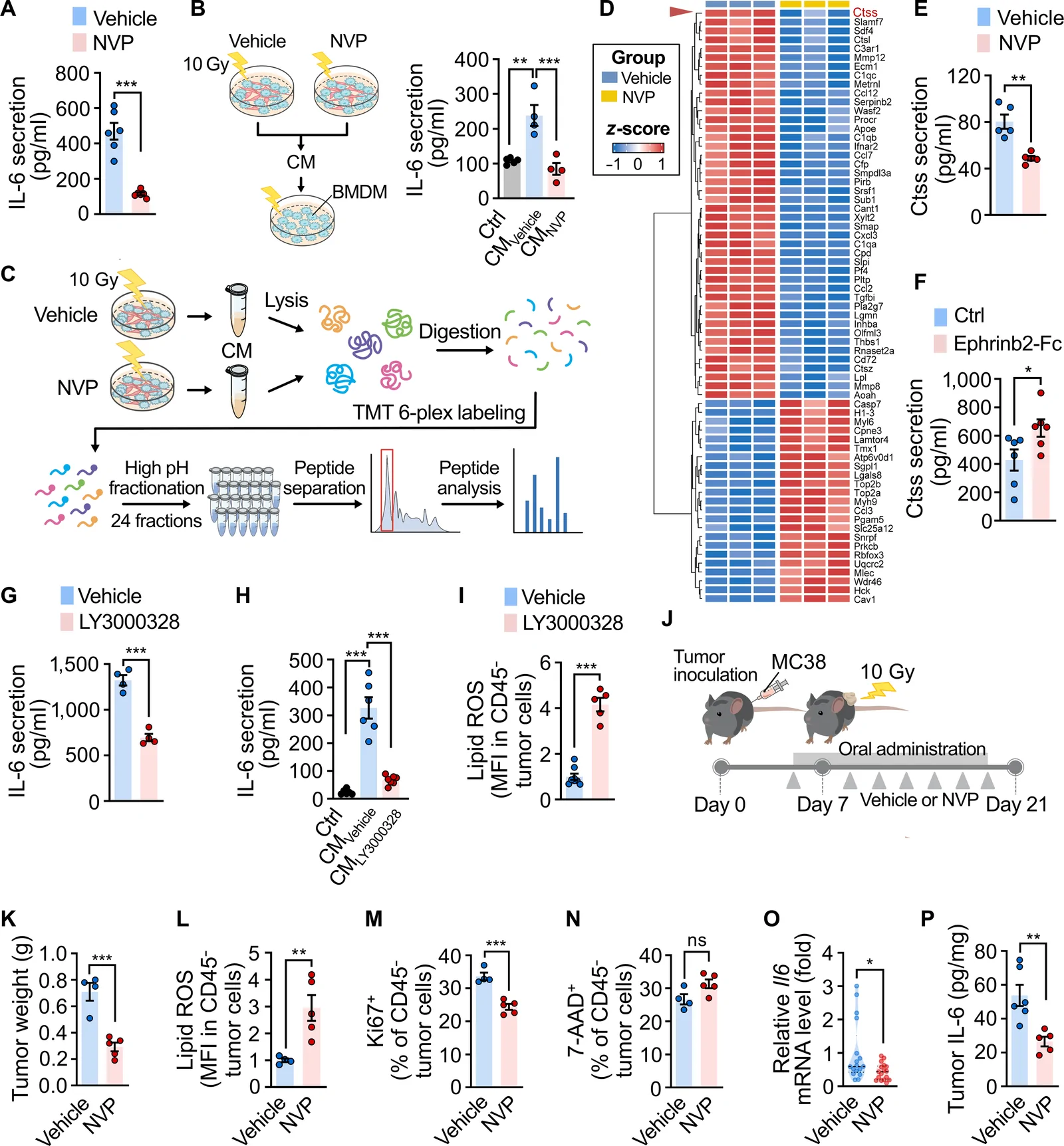
Pharmacological inhibition of Ephrin receptor b4 (Ephb4) restored ferroptotic sensitivity and enhanced radiotherapy (RT) efficacy through dual disruption of intrinsic and extrinsic resistance mechanisms. (A) Interleukin-6 (IL-6) secretion measured by enzyme-linked immunosorbent assay (ELISA) in supernatants from cocultures of MC38 tumor cells with bone marrow-derived macrophages (BMDMs) treated with NVP-BHG712 (NVP) (1 μmol/L) or vehicle, 2 d post-irradiation (IR) (*n* = 6 per group). (B) Schematic illustration of experimental setup (left). CM from tumor cell–BMDM cocultures treated with vehicle or NVP were transferred to naive BMDMs, and IL-6 secretion was measured at 2 d post-IR (right). Naive BMDMs without CM treatment served as control (Ctrl; *n* = 4 to 5 per group). (C) Schematic illustration of the workflow for tandem mass tag (TMT)-based quantitative secretome profiling from supernatants of irradiated tumor cells-BMDMs cocultures treated with vehicle or NVP, collected at 2 d post-IR. (D) Heatmap showing differentially secreted proteins in CM from irradiated tumor cell–BMDM cocultures treated with vehicle or NVP, as quantified by TMT-based quantitative proteomics. Proteins were considered differentially secreted based on a log_2_ fold change ≥ 0.5 and an false discovery rate (FDR) < 0.05. The color scale represents *z*-score protein abundance. (E) ELISA quantification of cathepsin S (Ctss) in CM from irradiated tumor cell–BMDM cocultures treated with vehicle or NVP (*n* = 5 per group). (F) Ctss secretion in MC38 tumor cells treated with recombinant ephrinb2-Fc (10 μg/mL) or untreated control, measured by ELISA in supernatants at 2 d post-IR. Data were pooled from 2 independent experiments (*n* = 6 per group). (G) IL-6 secretion measured by ELISA in supernatants from cocultures of MC38 tumor cells and BMDMs with Ctss inhibitor LY3000328 (40 μmol/L) or vehicle, 2 d post-IR (*n* = 4 per group). (H) CM from (G) were transferred to naive BMDMs; IL-6 secretion was measured by ELISA in recipient BMDMs at 2 d post-IR (*n* = 6 to 7 per group). (I) Relative lipid reactive oxygen species (ROS) in CD45^−^-gated MC38 tumor cells, analyzed by flow cytometry under coculture conditions with BMDMs, with (*n* = 5) or without (*n* = 8) LY3000328 treatment (40 μmol/L). Measurements were performed at 3 d post-IR. (J) Schematic illustration of the in vivo experimental setup. Mice bearing MC38 tumors were treated with vehicle or NVP (50 mg/kg) by oral gavage starting on day 6 after tumor implantation, followed by 10-Gy IR. (K to N) Tumor weight (K) and relative lipid ROS (L), and percentage of Ki67^+^ cells (M) and 7-aminoactinomycin D (7-AAD)^+^ dead cells (N) in CD45^−^ tumor cell-enriched populations from MC38 tumors treated with vehicle (*n* = 4) or NVP (*n* = 5) 14 d post-IR. (O and P) *Il6* mRNA expression (O) and IL-6 protein levels (P, measured by ELISA) in tumor lysates from irradiated MC38 mouse model treated with (*n* = 5) or without (*n* = 6) NVP. Data pooled from 2 independent experiments. All IR experiments were performed using a single dose of 10 Gy. Data are presented as means ± standard error of the mean (SEM) (A and B, E to I, K to P). ns, not significant, *P < 0.05, **P < 0.01, ***P < 0.001 by unpaired 2-tailed Student’s *t* test (A, E to G, I, K to P) or 1-way analysis of variance (ANOVA) with Tukey’s multiple comparison test (B and H). CM, conditioned medium.

Given our prior demonstration that ephrinb2 reverse signaling in macrophages drives IL-6 production (Fig. 6G), we initially assumed that blocking Ephb4 forward signaling with NVP-BHG712 would have little impact on IL-6 secretion in macrophages. However, unexpectedly, NVP-BHG712 significantly reduced IL-6 levels in irradiated tumor cell–macrophage cocultures (Fig. 7A). Flow cytometric analysis confirmed that IL-6 reduction was confined to macrophages, with no detectable change in IL-6 expression in tumor cells (Fig. S13A). Moreover, CM from vehicle-treated cocultures significantly induced IL-6 production in naive macrophages, whereas CM from NVP-BHG712-treated cultures did not (Fig. 7B). Direct treatment of macrophages with NVP-BHG712 had no effect on IL-6 secretion (Fig. S13B), suggesting the involvement of a tumor-derived soluble factor regulated by Ephb4.

To identify this factor, we performed quantitative secretome profiling of CM from irradiated tumor–macrophage cocultures treated with vehicle or NVP-BHG712 using mass spectrometry (Fig. 7C). Protein interaction network analysis revealed several affected functional clusters, including pathways related to the complement cascade, lysosomal function, translation, and chemotaxis (Fig. S13C). Notably, Ctss, a lysosomal cysteine protease known to regulate IL-6 production [54,55], emerged as the most prominently down-regulated candidate in CM from NVP-BHG712-treated tumor–macrophage cocultures (Fig. 7D). ELISA confirmed a significant reduction in Ctss levels in CM following Ephb4 inhibition (Fig. 7E). Conversely, stimulation of tumor cells with recombinant ephrinb2-Fc significantly enhanced Ctss secretion even in the absence of macrophages (Fig. 7F), indicating that Ctss acts as a tumor-intrinsic effector downstream of Ephb4.

Functionally, pharmacological inhibition of Ctss significantly reduced IL-6 secretion in irradiated tumor–macrophage cocultures (Fig. 7G). In addition, CM from Ctss-inhibited cocultures induced lower IL-6 secretion in naive macrophages than CM from vehicle-treated cocultures (Fig. 7H). Ctss inhibition also increased lipid peroxidation in irradiated tumor cells (Fig. 7I), consistent with reduced ferroptosis resistance.

We next examined how tumor-derived Ctss stimulates macrophage IL-6 production. Previous studies have shown that Ctss can engage Par2 signaling, which promotes IL-6 expression [56,57]. Consistent with this mechanism, CM from *Ephb4*-deficient tumor–macrophage cocultures resulted in reduced Par2 protein levels in macrophages compared with CM from WT cocultures (Fig. S14A). Ctss inhibition in irradiated tumor–macrophage cocultures reduced the ability of CM to induce Par2 expression in macrophages (Fig. S14B). Moreover, pharmacological blockade of Par2 significantly reduced CM-induced IL-6 secretion (Fig. S14C), indicating that tumor-derived Ctss promotes macrophage IL-6 production through Par2 signaling.

To evaluate the therapeutic potential of pharmacological Ephb4 inhibition in overcoming radioresistance in vivo, MC38 tumor-bearing mice were treated with NVP-BHG712 in combination with IR (Fig. 7J). Compared to vehicle-treated controls, NVP-BHG712 treatment significantly reduced tumor burden (Fig. 7K), increased lipid ROS (Fig. 7L), and decreased Ki67^+^ proliferative capacity (Fig. 7M), while showing a modest, nonsignificant increase in tumor cell death (Fig. 7N). Consistent with in vitro findings (Fig. 7A), NVP-BHG712 treatment led to significant reductions in *Il6* transcript and IL-6 protein levels in tumors following IR (Fig. 7O and P), a slight, nonsignificant decrease in Ctss protein levels in irradiated tumors (Fig. S15A). NVP-BHG712 treatment also reshaped the tumor immune landscape, reducing macrophage infiltration (Fig. S15B) and increasing infiltration of cytotoxic CD8^+^ T cells expressing IFN-γ and granzyme B (Fig. S15C to E). Similar patterns were observed in CT26 tumors (Fig. S16).

Together, these findings identified Ctss as a tumor-secreted mediator downstream of Ephb4 that promotes paracrine IL-6 production by macrophages. Accordingly, pharmacological inhibition of Ephb4 suppresses the Ephb4–Ctss–IL-6 axis, thereby enhancing radiation-induced ferroptosis and promoting an antitumoral immune microenvironment.

### Elevated expression of EPHB4 was associated with increased radioresistance and reduced ferroptotic sensitivity in esophageal and colorectal cancers

**Fig. 8.**
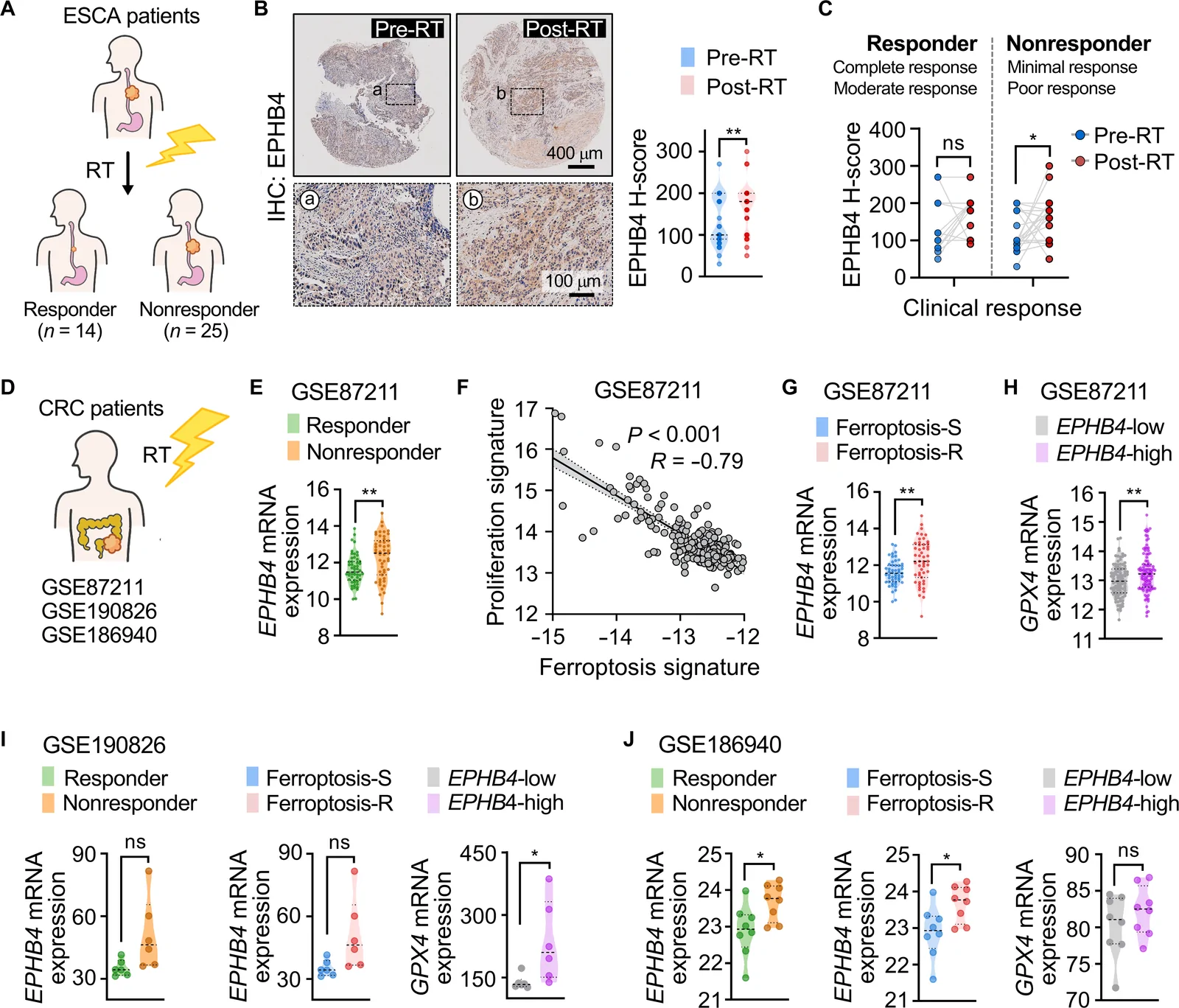
Elevated expression of Ephrin receptor b4 (EPHB4) is associated with increased radioresistance and reduced ferroptotic sensitivity in esophageal and colorectal cancers. (A) Schematic illustration of esophageal squamous cell carcinoma (ESCA) patient cohort stratified by radiotherapy (RT) response: responders (*n* = 14) and nonresponders (*n* = 25). (B) Representative immunohistochemistry (IHC) images of EPHB4 staining in ESCA tumors pre- and post-RT (left). Quantification of H-scores shown at right (*n* = 39 paired samples). (C) EPHB4 H-scores in ESCA responders and nonresponders before and after RT, based on clinical classification. (D) Schematic illustration of public data of post-RT colorectal cancer (CRC) cohorts analyzed in this study: GSE87211, GSE190826, and GSE186940. (E to H) Analyses based on the GSE87211 dataset. (E) *EPHB4* mRNA expression in RT responders and nonresponders (*n* = 51 per group). (F) Correlation between ferroptosis and proliferation signature scores across individual tumors. Each dot represents a single patient. (G) *EPHB4* expression in ferroptosis-sensitive (ferroptosis-S) vs. ferroptosis-resistance (ferroptosis-R) tumors (*n* = 51 per group). (H) Glutathione peroxidase 4 (*GPX4*) expression in *EPHB4* expression low (*n* = 100) vs. *EPHB4* expression high tumors (*n* = 102). Tumors were stratified by median *EPHB4* mRNA expression. (I and J) *EPHB4* and *GPX4* expression in additional datasets, GSE190826 (rectal cancer, *n* = 6 per group, I) and GSE186940 (CRC, *n* = 8 per group, J), stratified by response to RT, ferroptosis sensitivity status, or *EPHB4* expression level. Tumors were stratified by median *EPHB4* mRNA expression. Data are presented as means ± standard error of the mean (SEM) (B and C, E, and G to J). ns, not significant, *P < 0.05, **P < 0.01, by unpaired 2-tailed Student’s *t* test (B, E, and G to J) or 2-way analysis of variance (ANOVA) (C).

To assess the clinical relevance of EphB4-driven ferroptosis resistance, we first examined *EPHB4* transcript levels across tumor and normal tissues using The Cancer Genome Atlas. Excluding low-prevalence, sex-specific tumors such as testicular germ cell tumors [58], *EPHB4* expression was elevated in colorectal adenocarcinomas (colon adenocarcinoma and rectum adenocarcinoma) (Fig. S17), consistent with our findings in MC38 and CT26 models. Notably, esophageal carcinoma (ESCA) exhibited the next highest EPHB4 expression among the analyzed tumor types. Based on this, we next examined ESCA tissues to determine how EPHB4 expression is modulated by RT and whether it is associated with RT response. Analysis of matched tumor samples from ESCA patients pre- and post-RT revealed a significant increase in EPHB4 expression following treatment (Fig. 8A and B). Notably, stratification by clinical response showed that EPHB4 up-regulation occurred predominantly in nonresponders (Fig. 8C), suggesting a potential link between EPHB4 up-regulation and treatment resistance.

We then extended this analysis to colorectal cancer cohorts with annotated RT outcomes (Fig. 8D). In these datasets, nonresponders exhibited significantly higher *EPHB4* mRNA expression following treatment compared to responders (Fig. 8E). Across patient samples, ferroptosis signature scores were inversely correlated with proliferative gene signature (Fig. 8F), suggesting that ferroptosis evasion may facilitate tumor regrowth post-RT. Supporting this, EPHB4 expression was significantly elevated in tumors classified as ferroptosis-resistant as defined by the median expression score of a curated ferroptosis gene set (Fig. 8G). Additionally, when tumors were stratified by *EPHB4* expression level, *GPX4* mRNA levels were significantly higher in the *EPHB4*-high group (Fig. 8H). These observations were validated across 2 independent colorectal cancer cohorts (GSE190826 and GSE186940), where *EPHB4*-high tumors showed a trend toward reduced ferroptosis activity and increased *GPX4* expression, along with features associated with radioresistance, although not all comparisons reached statistical significance (Fig. 8I and J).

Collectively, these data suggest that EPHB4 expression is associated with ferroptosis-related features and may be linked to radioresistance in colorectal cancer.

## Discussion

RT imposes substantial oxidative and metabolic stress on tumor cells, yet a subset can persist in a therapy-tolerant state [7,8,59]. The mechanisms by which these persister cells adapt to radiation-induced stress and acquire radioresistance remain incompletely understood, particularly in the context of cellular interactions within the TME that suppress cell death post-RT. Here, we identified a macrophage-dependent, contact-initiated ferroptosis evasion pathway mediated by bidirectional Ephb4–ephrinb2 signaling. This interaction enables radiation-surviving tumor cells to withstand ferroptotic death and progressively adopt a resistant phenotype. Notably, given that Ephb4 up-regulation is mediated by HIF-1α signaling, this pathway may also be linked to hypoxia-associated radioresistance.

Ferroptosis is an iron-dependent form of regulated cell death characterized by lipid peroxide accumulation [60,61]. Ionizing radiation can trigger this pathway by elevating intracellular ROS and inducing oxidative membrane damage [45,62]. To counteract this lethal stress, tumor cells activate antioxidant defenses such as the solute carrier family 7 member 11 (SLC7A11)–glutathione peroxidase 4 (GPX4) axis [42,45,63] or the nicotinamide adenine dinucleotide phosphate–ubiquinone–ferroptosis suppressor protein 1 system [64,65], both of which preserve redox homeostasis and prevent lipid peroxidation. The therapeutic relevance of ferroptosis has garnered interest in the context of drug resistance, as drug-tolerant persister cells are susceptible to ferroptotic death due to compromised antioxidant defenses [66–68]. Likewise, radiation-surviving tumor cells may transiently exhibit impaired redox capacity, placing them in a ferroptosis-prone state. To survive oxidative pressure during treatment-free intervals, they increasingly depend on ferroptosis-suppressive mechanisms [42,45]. Therefore, early suppression of ferroptosis may represent a decisive checkpoint that determines whether residual cells progress toward durable radioresistance.

**Fig. 9.**
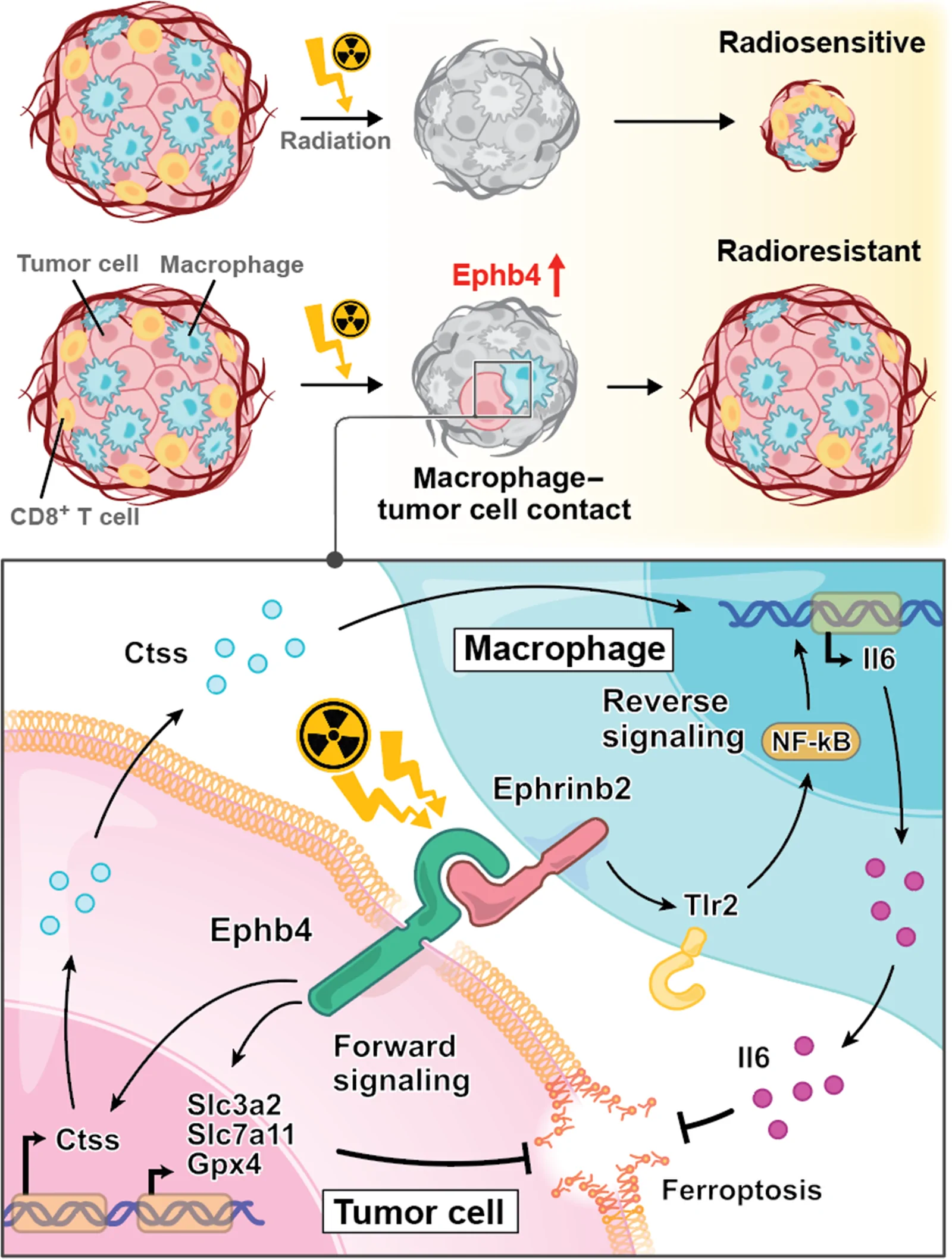
Schematic illustration of the macrophage–tumor cell juxtacrine-paracrine loop driving ferroptosis resistance of tumor cells after irradiation. Upon irradiation, tumor cells up-regulate Ephrin receptor b4 (Ephb4), which engages with ephrinb2 on macrophages through direct contact, initiating bidirectional Ephb4–ephrinb2 signaling. Forward signaling in tumor cells induces the expression of antiferroptotic genes (solute carrier family 7 member 11 [*Slc7a11*], solute carrier family 3 member 2 [*Slc3a2*], and glutathione peroxidase 4 [*Gpx4*]), while reverse signaling in macrophages activates the toll-like receptor 2 (Tlr2)-nuclear factor-kappa B (NF-κB) pathway, leading to IL-6 secretion. In parallel, Ephb4-driven secretion of cathepsin S (Ctss) by tumor cells further enhances macrophage IL-6 production through a paracrine mechanism. Elevated macrophage-derived IL-6, in turn, sustains antiferroptotic gene expression in tumor cells, thereby suppressing irradiation-induced lipid peroxidation, protecting against ferroptotic death, and promoting the emergence of radioresistant tumor cells.

In this context, we demonstrated that tumor cells engage macrophages via direct contact to initiate a juxtacrine-paracrine circuit that cooperatively reprograms both compartments (Fig. 9). Ephb4 forward signaling in tumor cells induces the expression of ferroptosis-protective genes, including *Slc7a11*, *Slc3a2*, and *Gpx4*, and promotes the secretion of Ctss. Concurrently, ephrinb2 reverse signaling in macrophages activates a Tlr2–NF-κB pathway, leading to IL-6 secretion. Tumor-derived Ctss further enhances IL-6 production, creating a paracrine feedback loop that reinforces ferroptosis resistance throughout the tumor niche. Supporting these findings, single-cell transcriptomic analysis of irradiated ESCA tissues showed IL-6 enrichment in myeloid populations, particularly macrophages [18], confirming the clinical relevance of macrophage-derived IL-6 as a key stromal cue post-RT.

This macrophage–tumor cell crosstalk, initiated by physical contact, exerts paracrine effects across the TME. The Ephb4–ephrinb2-Ctss-IL-6 axis disseminates ferroptosis-protective signals, protecting neighboring tumor cells and contributing to immune evasion through IL-6-mediated CD8^+^ T cell suppression [69,70]. Consequently, therapeutic disruption of Ephb4 impairs both tumor-intrinsic antioxidant defenses and macrophage-derived IL-6 support, thereby restoring lipid peroxidation and radiosensitivity. Furthermore, Ephb4 inhibition remodels the immune landscape by restricting the infiltration of immunosuppressive macrophages and reinvigorating CD8^+^ T cell effector responses. The clinical relevance of this axis is underscored by transcriptomic analysis of patients with colorectal cancer. Notably, nonresponders to RT exhibit a coordinated up-regulation of *EPHB4* and *GPX4* alongside repressed ferroptosis signatures, suggesting that this survival circuit is conserved in human colorectal cancers and may serve as a biomarker of therapeutic failure.

However, several limitations of this study should be considered. First, the TME is highly complex, and the contribution of stromal components was not fully addressed. While our study primarily focused on tumor-intrinsic mechanisms, vascular endothelial cells are also known to express substantial levels of Ephb4, which regulates vascular remodeling and cell trafficking [38,71,72]. In particular, endothelial Eph/ephrin signaling, including Ephb4 as well as related receptors such as Epha4 and Ephb2, has been reported to promote monocyte adhesion and migration through interactions with ephrin ligands [73,74]. Therefore, it is plausible that endothelial Ephb4 cooperates with tumor-derived signals to facilitate the initial recruitment of myeloid cells into the TME, a process that was not directly examined in this study. Second, the downstream signaling cascade linking ephrinb2 reverse signaling to Tlr2–NF-κB-mediated IL-6 production in macrophages remains to be fully elucidated. Finally, as our findings were validated in murine colorectal tumor models, further validation across diverse tumor types will be required to determine the broader applicability of this signaling axis. Considering the context-dependent roles of Eph receptors in cancer [75,76], tumor-specific evaluation will be essential for the clinical translation of Ephb4–ephrinb2-targeted therapeutic strategies.

Taken together, our findings demonstrate that therapy-tolerant tumor cells actively engage macrophages to create a ferroptosis-resistant niche that enhances survival and regrowth after IR. Ephb4 signaling is crucial in sustaining this state, acting as a central regulator of macrophage-driven resistance. Targeting the Ephb4–Ctss–IL-6 axis may therefore offer a therapeutic opportunity to disrupt this protective niche and improve long-term outcomes in radioresistant colorectal tumors.

## Conclusions

Our study establishes Ephb4–ephrinb2 signaling as a mechanistic link between tumor–macrophage contact and ferroptosis resistance following IR. By coordinating tumor-intrinsic antioxidant adaptation with macrophage-derived IL-6 support, this pathway promotes the persistence of irradiated tumor cells and the development of durable radioresistance. Genetic or pharmacological disruption of this circuit restores ferroptotic vulnerability, remodels the immune microenvironment, and enhances RT efficacy. Collectively, these findings identify macrophage–tumor cell crosstalk as a key regulator of ferroptosis evasion and provide a rationale for Ephb4-directed intervention as a therapeutic strategy to overcome radioresistance.

## Ethical Approval

All animal experiments were conducted in accordance with protocols approved by the IACUC of Seoul National University (IACUC No. SNU-210122-3-3). The IRB waived the need for written informed consent under the conditions of anonymization and no additional intervention. This study was approved by the IRB of Seoul National University Hospital (IRB number H-2301-100-1396) and performed in accordance with the recommendations of the Declaration of Helsinki for biomedical research involving human subjects.

## Data Availability

The bulk RNA-seq raw data and files have been deposited in the Gene Expression Omnibus and are publicly available through accession number GSE302141. The mass spectrometry proteomics data have been deposited to the ProteomeXchange Consortium via the PRIDE partner repository with the dataset identifier PXD06583.
