# Supplementary material for: Macrophages Drive Ferroptosis Resistance after Radiotherapy
Source: Cancer Commun (Lond). 2026 Sep 4;46:0039. doi: 10.34133/cancomm.0039 (PMC13542434; doi:10.34133/cancomm.0039)
Supplement: Supplementary file 1 [file cancomm.0039.f1.pdf]

Supplementary Materials for

Macrophages drive ferroptosis resistance after radiotherapy

Hyewon Chung[1,2,†], Sang Wha Kim[3,†], Jae Won Oh[4], Gyu Mi Park[1,5], Yurim Cho[1,5], Hae Suk Choi[1,3], MinJi Kim[6], Kwang Pyo Kim[4,7], Yi Rang Na[5,8,9], Hye Seung Lee[6,10], Hak Jae Kim[6,11,*], Seung Hyeok Seok[1,2,5,6,*]

[1]Department of Microbiology and Immunology, Seoul National University College of Medicine, Seoul, South Korea
[2]Institute of Endemic Diseases, Seoul National University Medical Research Center, Seoul, South Korea
[3]College of Veterinary Medicine & Institute of Veterinary Science, Kangwon National University, Chuncheon, Gangwon, South Korea
[4]Department of Applied Chemistry, Institute of Natural Science, Global Center for Pharmaceutical Ingredient Materials, Kyung Hee University, Yongin, South Korea
[5]Department of Biomedical Sciences, Seoul National University College of Medicine, Seoul, South Korea
[6]Cancer Research Institute, Seoul National University, Seoul, South Korea
[7]Department of Biomedical Science and Technology, Kyung Hee Medical Science Research Institute, Kyung Hee University, Seoul, South Korea
[8]Translational Immunology Lab, Department of Transdisciplinary Medicine, Seoul National University Hospital, Seoul, South Korea
[9]Immunology Core Facility, Advanced Omics Research Core, Biomedical Research Institute, Seoul National University Hospital, Seoul, South Korea
[10]Department of Pathology, Seoul National University Hospital, Seoul National University College of Medicine, Seoul, South Korea
[11]Department of Radiation Oncology, Seoul National University College of Medicine and Hospital, Seoul, South Korea

[†]Hyewon Chung and Sang Wha Kim contributed equally to this study.

***Correspondence authors**
Seung Hyeok Seok

E-mail: lamseok@snu.ac.kr

Hak Jae Kim
E-mail: khjae@snu.ac.kr

Supplementary Figures

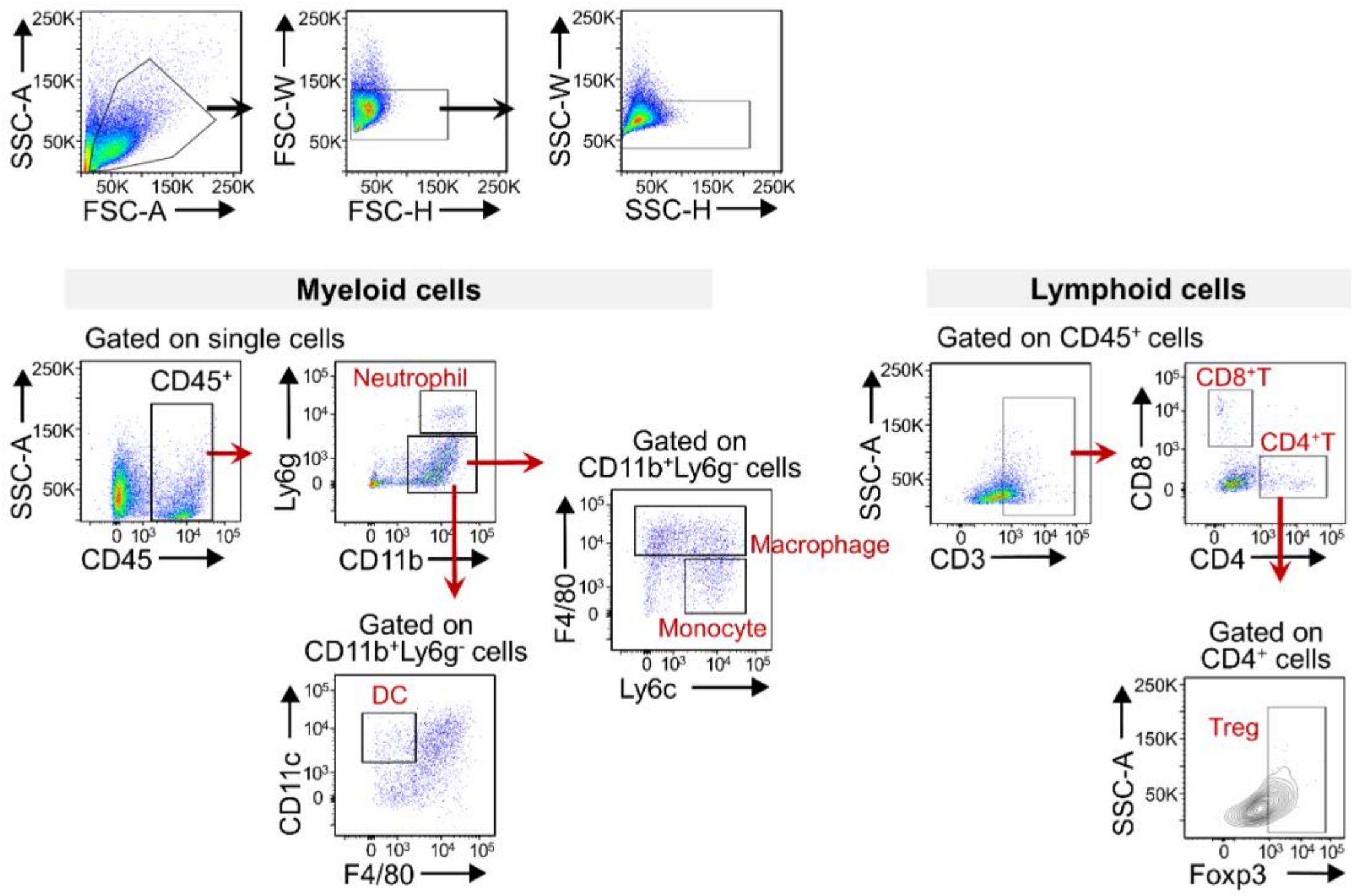

Figure S1. Gating strategy for flow cytometric analysis of tumor-infiltrating myeloid and lymphoid immune cell subsets. Abbreviations: CD, cluster of differentiation; DC, dendritic cell; Foxp3, forkhead box p3; FSC, forward scatter; SSC, side scatter; Treg, regulatory T cell.

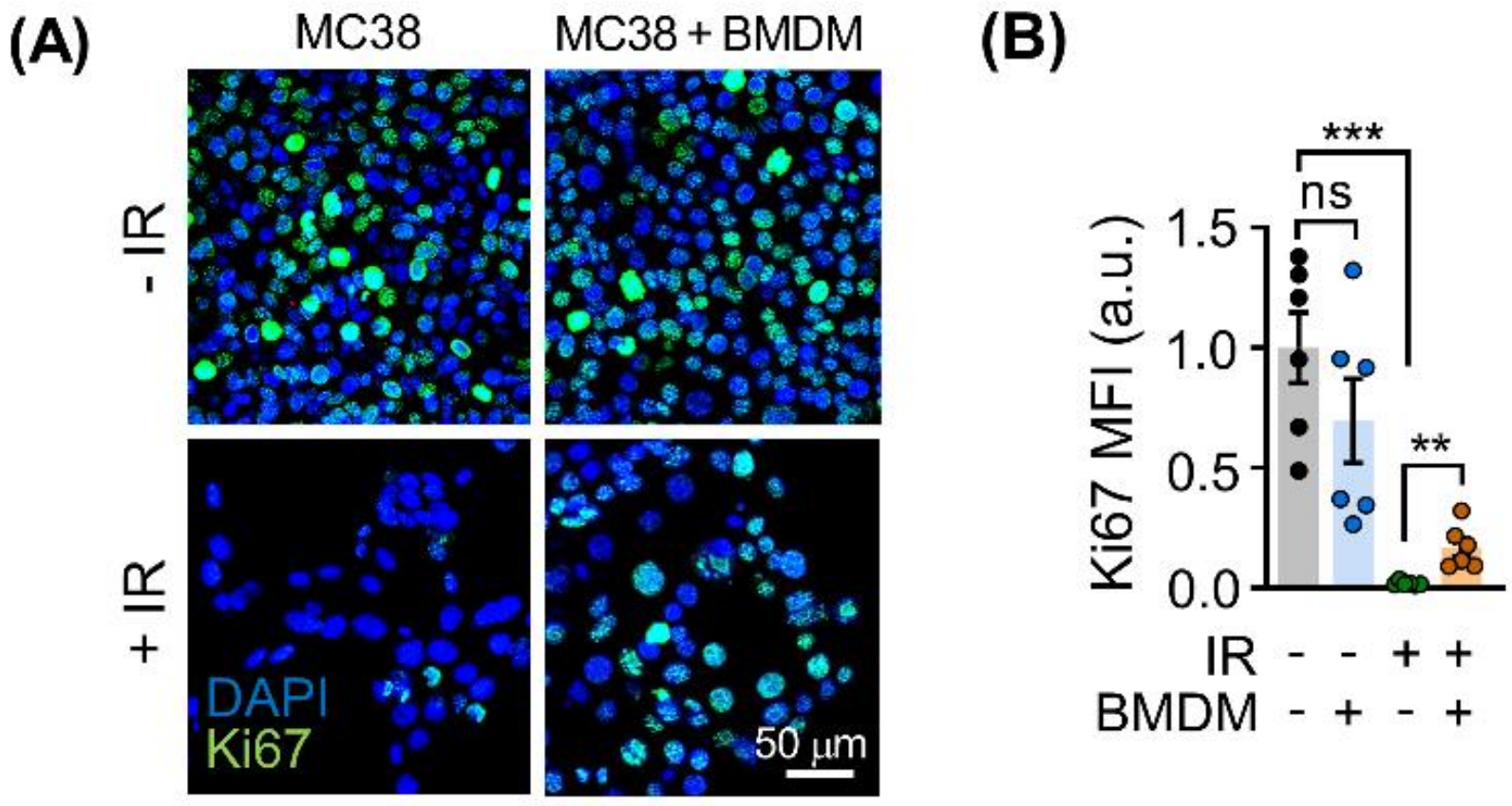

Figure S2. Macrophages promoted tumor cell proliferation after irradiation. (A) Representative IF staining using Ki67 (green) and DAPI (blue) in MC38 tumor cells cultured alone or with BMDMs, with or without IR at day 4 post-IR (10 Gy). **(B)** Quantification of Ki67 MFI in MC38 tumor cells under the indicated conditions in (A). Data are presented as mean ± SEM (B). ns, not significant, ***P* < 0.01, ****P* < 0.001 by unpaired two-tailed Student's t test (B). Abbreviations: a.u., arbitrary units; BMDM, bone marrow-derived macrophage; DAPI, 4′,6-diamidino-2-phenylindole; IF, immunofluorescence; IR, irradiation; MFI, mean fluorescence intensity; SEM, standard error of the mean.

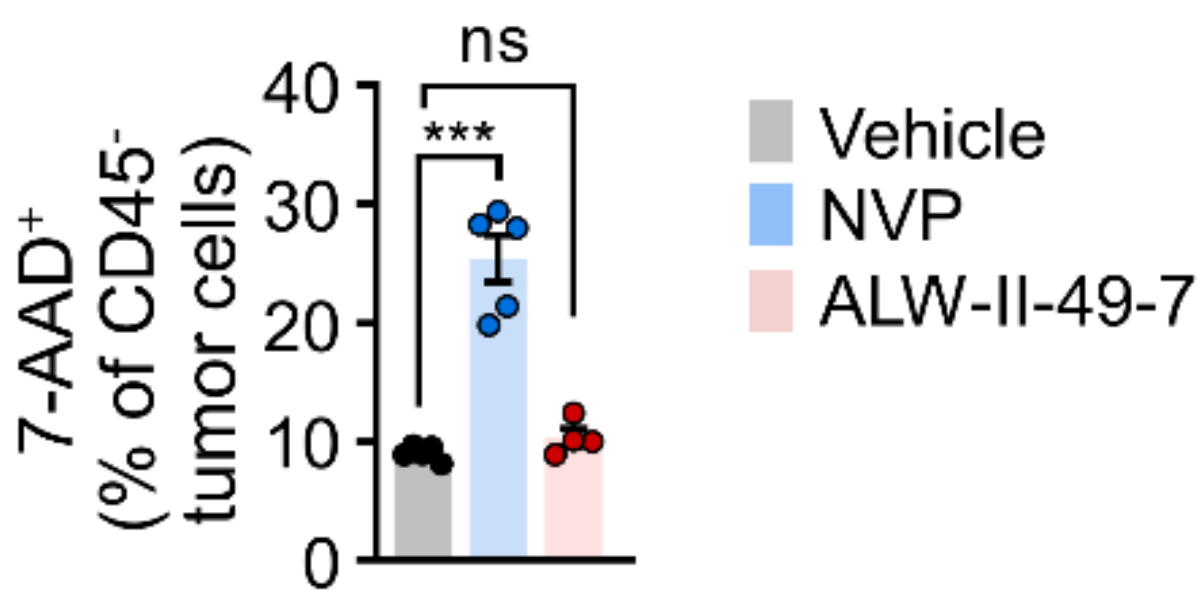

Figure S3. Ephb4, but not Ephb2, was implicated in macrophage-mediated tumor cell death after irradiation. Percentage of 7-AAD$^+$ dead cells in CD45$^-$ MC38 tumor cells co-cultured with BMDMs. MC38 tumor cells were first co-cultured with BMDMs, treated with vehicle (n = 5), the Ephb4 inhibitor NVP-BHG712 (NVP, 1 µmol/L, n = 5), or the Ephb2 inhibitor ALW-II-49-7 (1 µmol/L, n = 4), and then irradiated the following day (10 Gy). Tumor cell death was analyzed 4 days after IR. Data are presented as mean ± SEM. ns, not significant, ***P < 0.001 by unpaired two-tailed Student's t test. Abbreviations: BMDM, bone marrow-derived macrophage; IR, irradiation; NVP, NVP-BHG712; SEM, standard error of the mean.

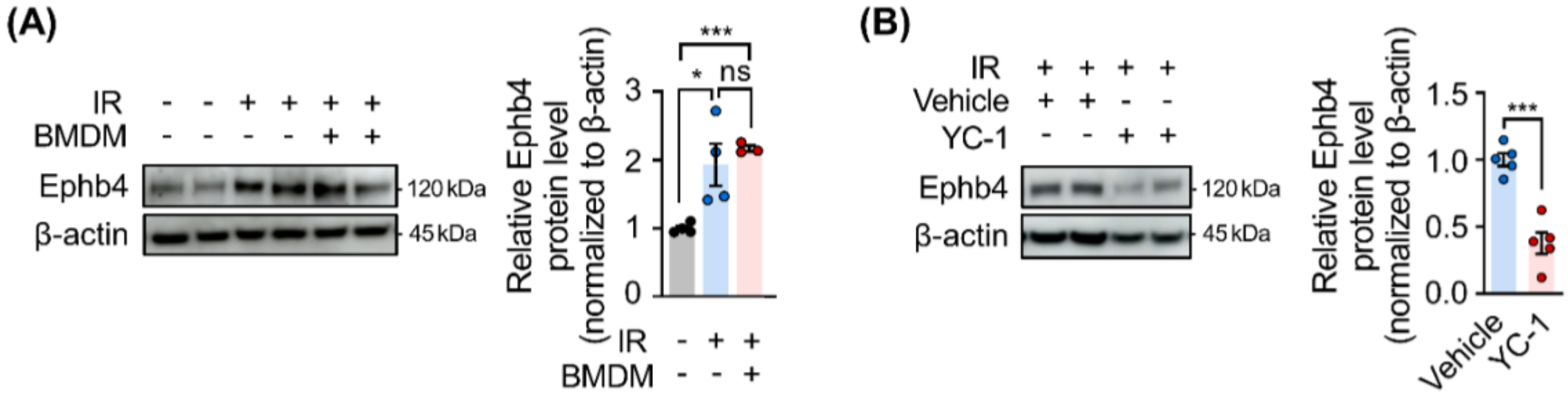

Figure S4. Irradiation induced Ephb4 expression in tumor cells in a HIF-1α-dependent manner. (A) Immunoblotting analysis of Ephb4 protein expression in MC38 tumor cells under the following conditions: non-irradiation (control; *n* = 4), irradiation following culture alone (*n* = 4), or irradiation following co-culture with BMDMs (*n* = 3). Tumor cells were collected and analyzed 48 h after irradiation (10 Gy). Representative blots (left) and quantification of Ephb4 protein levels normalized to β-actin (right) are shown. **(B)** Immunoblotting analysis of Ephb4 expression in MC38 tumor cells 48 h post-IR, with or without the HIF-1α inhibitor YC-1 (50 μmol/L) treatment. Representative blots (left) and quantification of the Ephb4 protein levels normalized to β-actin under vehicle or YC-1-treated conditions are shown (*n* = 6 per group). Data are presented as mean ± SEM (A-B). ns, not significant, **P* < 0.05, ****P* < 0.001 by one-way ANOVA with Tukey's multiple comparison test (A) or unpaired two-tailed Student's t test (B). Abbreviations: BMDM, bone marrow-derived macrophage; Ephb4, ephrin receptor b4; IR, irradiation; SEM, standard error of the mean.

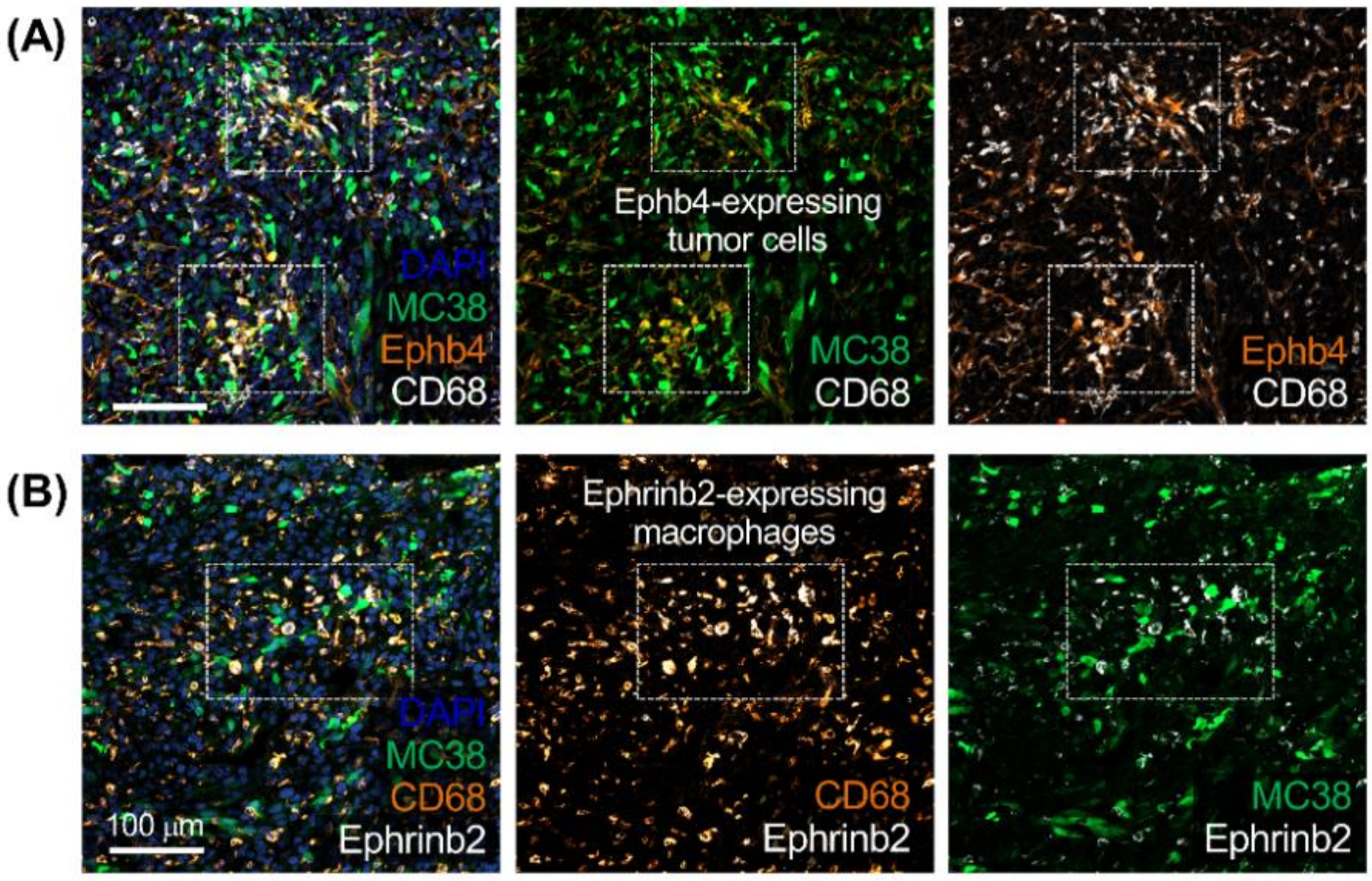

Figure S5. Spatial association of Ephb4-expressing tumor cells and ephrinb2-expressing macrophages in irradiated tumors in vivo.

(A) Representative IF images of GFP-expressing MC38 tumor cells (green), Ephb4 (orange), CD68 (macrophage marker, white), and DAPI (blue) in irradiated MC38-GFP tumors at day 14 post-IR (10 Gy). Ephb4-expressing tumor cells were frequently observed in regions enriched with $CD68^+$ macrophages, with close spatial proximity highlighted in dashed boxes. **(B)** Representative IF images of GFP-expressing MC38 tumor cells (green), Ephrinb2 (white), CD68 (macrophage marker, orange), and DAPI (blue) in irradiated MC38-GFP tumors at day 14 post-IR. Ephrinb2-expressing macrophages were preferentially located in close proximity to tumor cells (dashed box). Abbreviations: DAPI, 4′,6-diamidino-2-phenylindole; Ephb4, ephrin receptor b4; GFP, green fluorescent protein; IF, immunofluorescence; IR, irradiation.

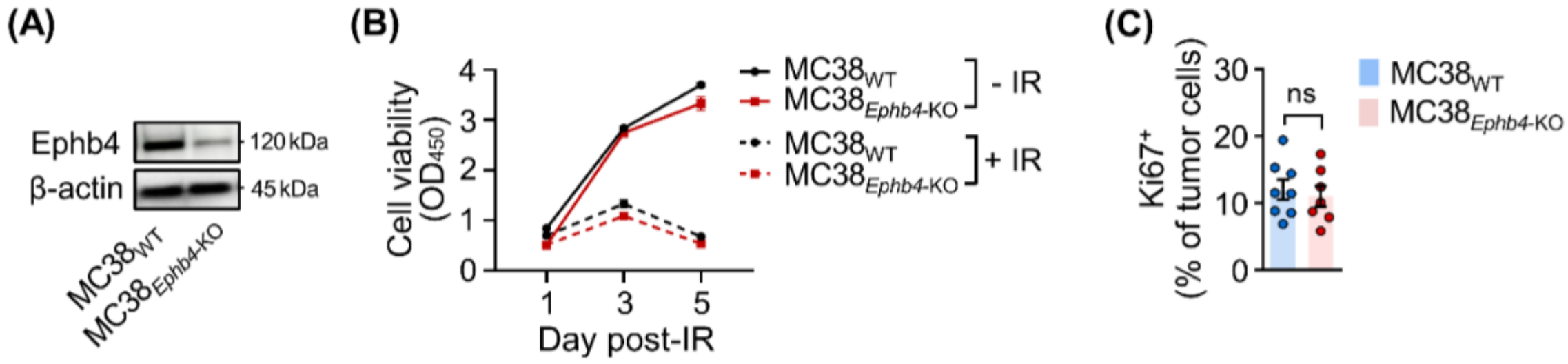

Figure S6. *Ephb4* deficiency did not affect tumor cell viability or proliferation in the absence of macrophages. (A) Immunoblotting analysis confirming the loss of Ephb4 protein expression in *Ephb4*-KO MC38 tumor cells compared to that in WT controls. **(B)** Time-course analysis of MC38 tumor cell viability over 5 days following 10 Gy irradiation, assessed by CCK-8 assay. Tumor cells were cultured without macrophages. Solid lines indicate absence of irradiation; dashed lines indicate irradiated conditions (n = 4-5 per group). **(C)** Percentage of $Ki67^+$ tumor cells in WT and *Ephb4*-KO conditions on day 4 post-IR in the absence of macrophages (n = 7 per group). Data are presented as mean ± SEM (C). ns, not significant by unpaired two-tailed Student's t test. Abbreviations: CCK-8, cell counting kit-8; Ephb4, ephrin receptor b4; IR, irradiation; KO, knockout; SEM, standard error of the mean; WT, wildtype.

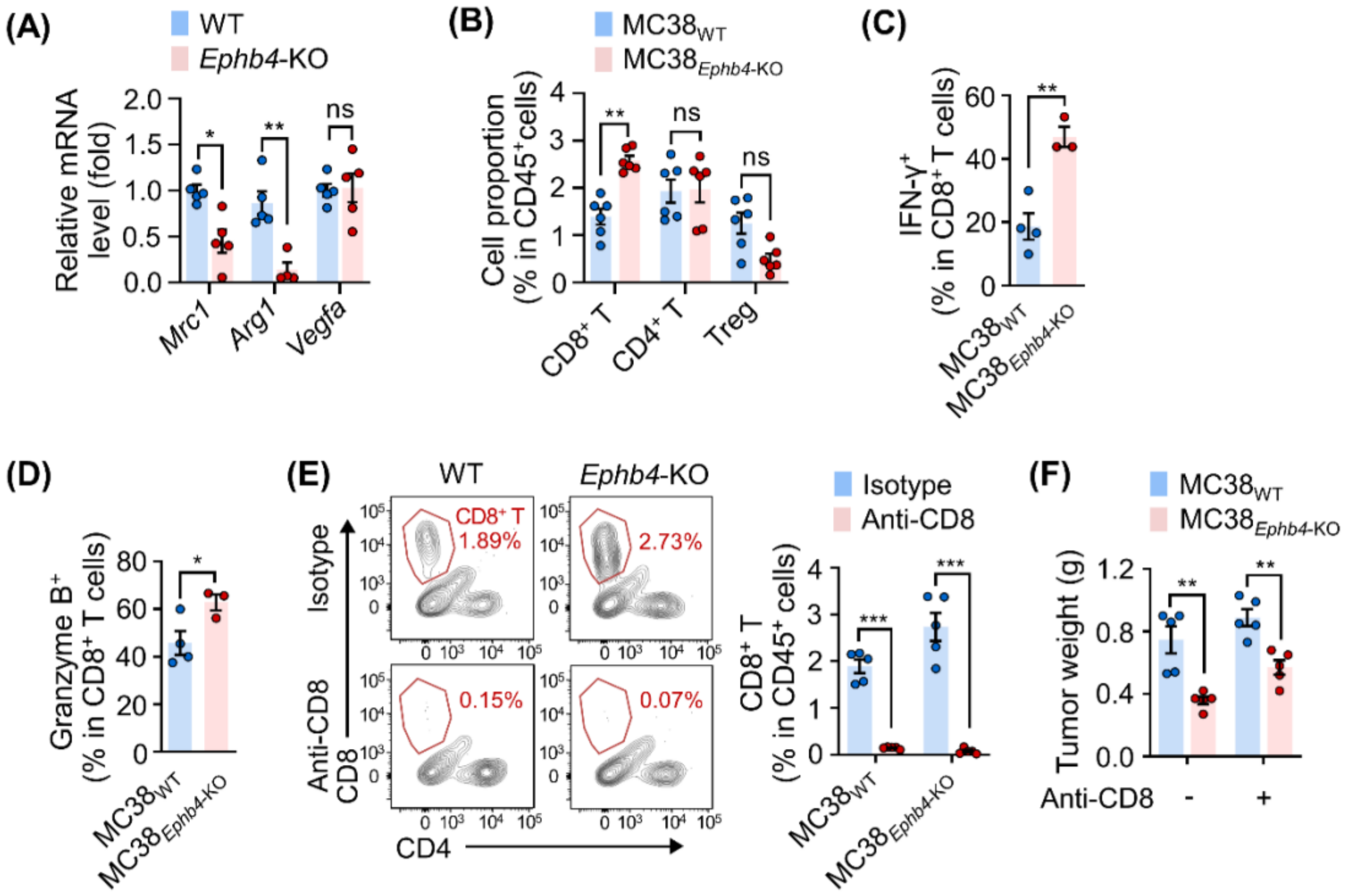

Figure S7. *Ephb4* deletion reprogrammed immune composition and enhances CD8⁺ T cell activation following irradiation.

(A) Relative mRNA expression of M2 macrophage-associated genes (*Mrc1*, *Arg1*, and *Vegfa*) in MC38 WT and *Ephb4*-KO tumors 7 days post-IR (*n* = 4-5 per group). **(B)** Flow cytometric analysis of tumor-infiltrating lymphocyte subsets (CD8⁺ T, CD4⁺ T, and Treg) in MC38 WT and *Ephb4*-KO tumors 7 days post-IR (*n* = 6 per group). **(C)** Percentage of IFN-γ⁺ in CD8⁺ T cells in MC38 WT and *Ephb4*-KO tumors 21 days post-IR (*n* = 3-4 per group). **(D)** Percentage of granzyme B⁺CD8⁺ T cells in MC38 WT and *Ephb4*-KO tumors 21 days post-IR (*n* = 3-4 per group). **(E)** Representative flow cytometry plots and percentage of CD8⁺ T cells at day 21 post-IR in mice bearing WT or *Ephb4*-deficient MC38 tumors with or without CD8⁺ T cell depletion using anti-CD8 antibody or isotype, respectively (*n* = 5 per group). **(F)** Tumor weight at day 21 post-IR in mice bearing WT or *Ephb4*-deficient MC38 tumors with or without CD8⁺ T cell depletion using anti-CD8 antibody or isotype, respectively (*n* = 5 per group). Data are presented as mean ± SEM (A-F). ns, not significant, *$P < 0.05$, **$P < 0.01$, ***$P < 0.001$ by two-way ANOVA (A-B, E, F) or unpaired two-tailed Student's *t* test (C-D). Abbreviations: Arg1, arginase 1; Ephb4, ephrin receptor b4; IFN-γ, interferon-γ; IR, irradiation; KO, knockout; Mrc1, mannose receptor c-type 1; SEM, standard error of the mean; Treg, regulatory T cell; Vegfa, vascular endothelial growth factor a; WT, wildtype.

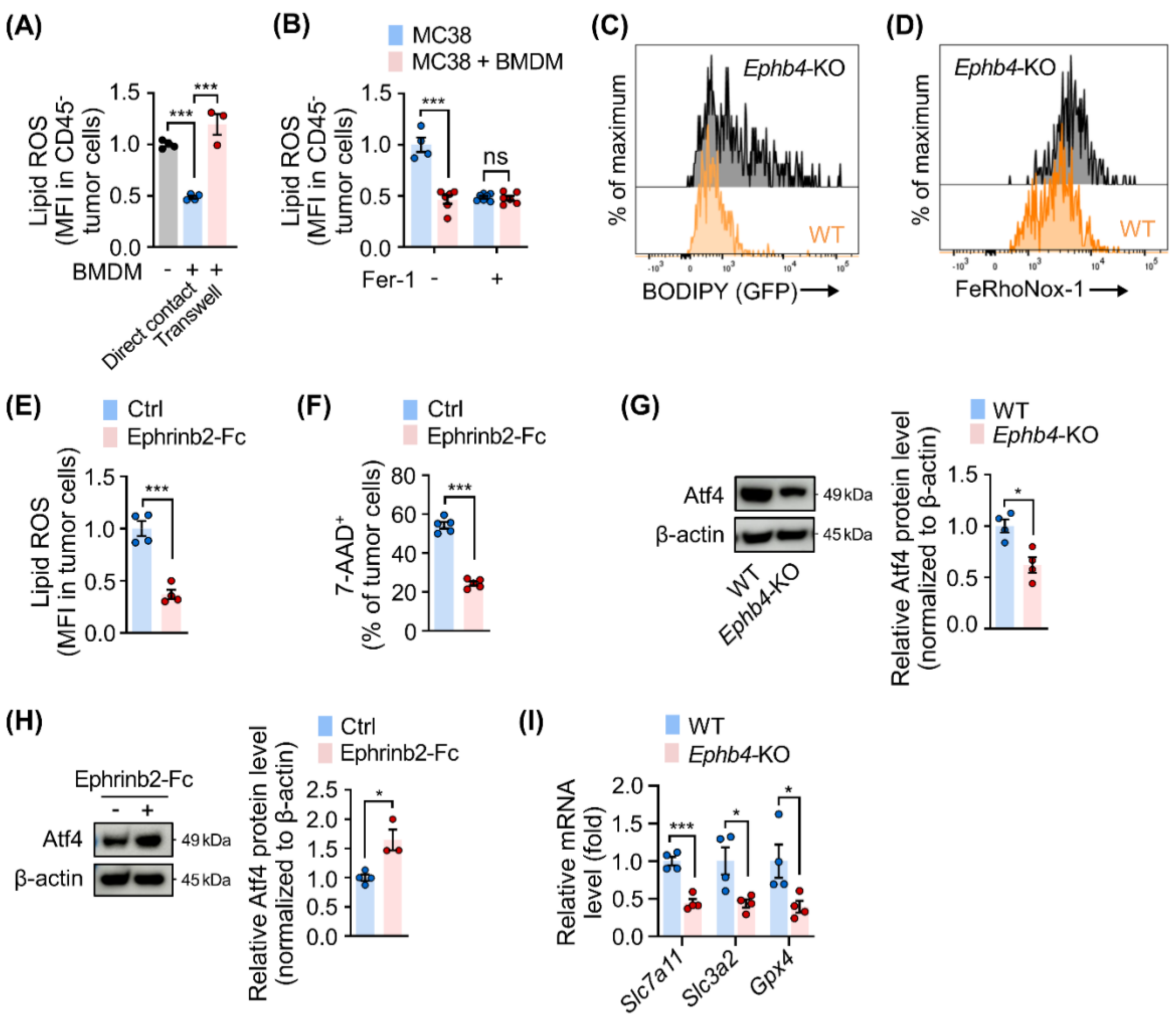

Figure S8. Ephb4–ephrinb2 signaling suppressed radiation-induced lipid peroxidation in tumor cells in an Atf4-dependent manner. (A) Relative lipid ROS in $CD45^-$ MC38 tumor cells cultured alone or co-cultured with BMDMs under either direct contact or Transwell-separated conditions, 3 days post-IR (*n* = 3–4 per group). **(B)** Relative lipid ROS in tumor cells co-cultured with or without BMDMs and treated with or without ferroptosis inhibitor ferrostatin-1 (Fer-1, 10 μmol/L; *n* = 4-6 per group), 3 days post-IR. **(C-D)** Representative flow cytometry histograms showing lipid ROS levels (C) and intracellular iron levels (D) in $CD45^-$-gated WT and *Ephb4*-KO tumor cells analyzed directly under co-culture conditions with BMDMs, 3 days post-IR. Lipid ROS and intracellular iron were measured using BODIPY-C11 and FeRhoNox-1, respectively. **(E-F)** Relative lipid ROS (E, *n* = 4 per group) and percentage of 7-AAD^+ dead cells (F, *n* = 5 per group) in MC38 WT tumor cells treated with or without recombinant ephrinb2-Fc (10 μg/mL). Lipid ROS was measured 3 days post-IR, and cell death was assessed 4 days post-IR. **(G)** Immunoblotting analysis of Atf4 expression in WT and *Ephb4*-KO tumor cells isolated by magnetic separation after 3 days of co-culture with

BMDMs following IR (as shown in Figure 4A). Right, quantification of Atf4 protein levels normalized to β-actin (*n* = 4 per group). **(H)** Immunoblotting analysis of Atf4 expression in MC38 tumor cells treated with recombinant ephrinb2-Fc (10 μg/mL) or vehicle control, 24 h post-IR. Right, quantification of Atf4 protein levels normalized to β-actin (*n* = 4 for vehicle control; *n* = 3 for ephrinb2-Fc). **(I)** Relative mRNA expression of ferroptosis-suppressing genes (*Slc7a11*, *Slc3a2*, and *Gpx4*) in WT and *Ephb4*-KO tumor cells isolated by magnetic separation after 3 days of co-culture with BMDMs following IR, as shown in Figure 4A (*n* = 4 per group). All IR experiments were performed using a single dose of 10 Gy. Data are presented as mean ± SEM (A-B, E-I). ns, not significant, *$P < 0.05$, ***$P < 0.001$ by unpaired two-tailed Student's *t* test (E-H), one-way ANOVA with Tukey's multiple comparison test (A) or two-way ANOVA (B, I). Abbreviations: Atf4, activating transcription factor 4; BMDM, bone marrow-derived macrophage; Ctrl, control; Ephb4, ephrin receptor b4; Fer-1, ferrostatin-1; FITC, Fluorescein isothiocyanate; Gpx4, glutathione peroxidase 4; IR, irradiation; KO, knockout; MFI, mean fluorescence intensity; ROS, reactive oxygen species; SEM, standard error of the mean; Slc3a2, solute carrier family 3 member 2; Slc7a11, solute carrier family 7 member 11; WT, wildtype.

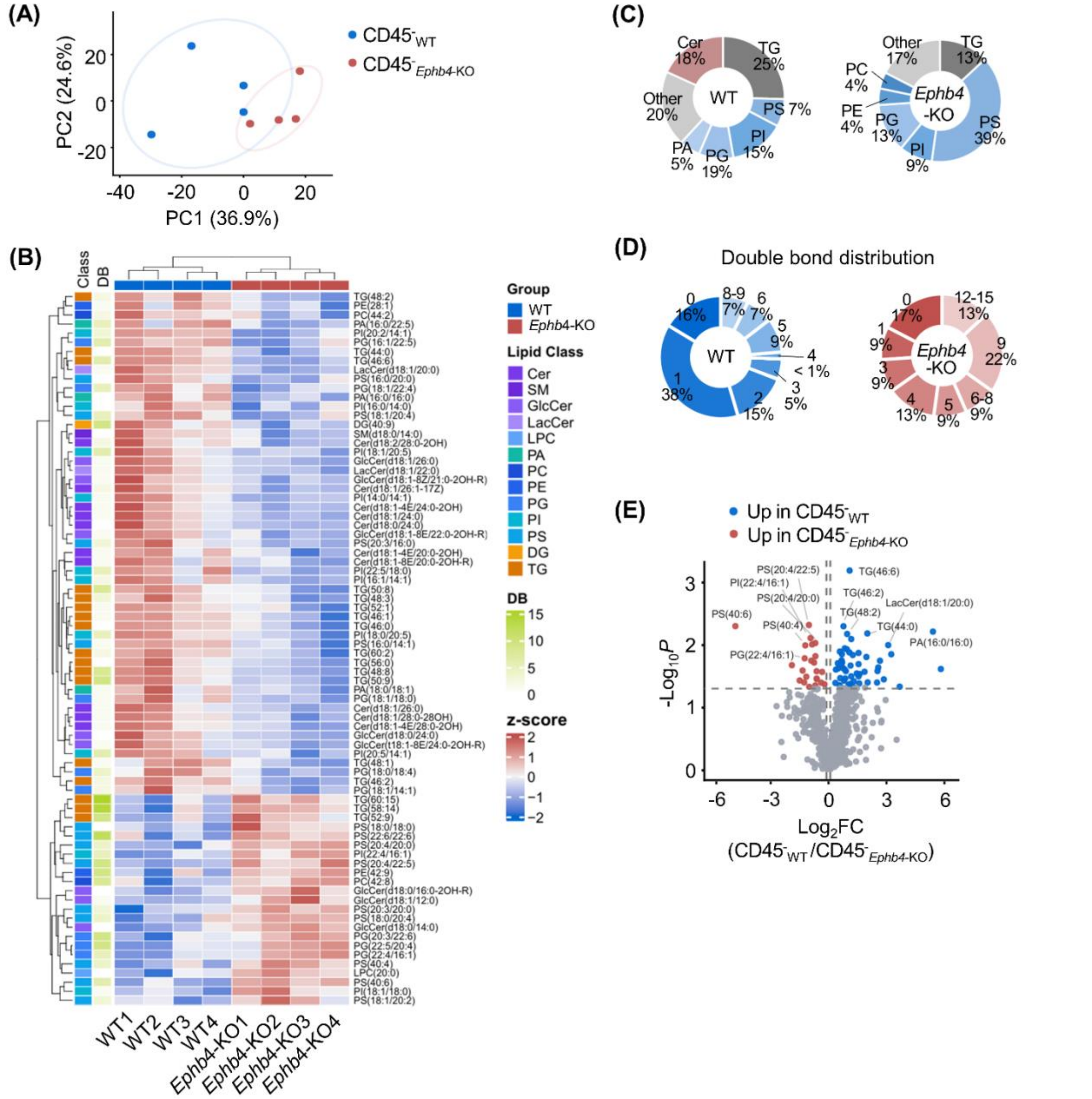

Figure S9. ***Ephb4*** **deficiency remodeled the tumor lipidome toward a ferroptosis-prone state. (A)** Targeted lipidomic profiling was performed on CD45$^-$ tumor cells isolated from WT and *Ephb4*-KO MC38 tumor cells (*n* = 4 per group) following 3 days of co-culture with BMDMs after 10 Gy IR. Tumor cells were purified from co-cultures using anti-mouse CD45 magnetic microbeads prior to lipidomic analysis. PCA of targeted lipidomic profiles was performed using normalized relative abundances of the 768 optimized MRM transitions. Each dot represents an individual biological replicate. Ellipses represent 95% confidence intervals for each group. **(B)** Hierarchical clustering heatmap of significantly (P < 0.05) altered lipid species across individual samples in WT and *Ephb4*-KO MC38 tumor cells (*n* = 4 per group). Relative abundances were log-transformed and converted to z-scores for each lipid species

prior to clustering. Both rows (lipid species) and columns (samples) were hierarchically clustered using Euclidean distance and complete linkage, revealing group-dependent lipid clustering patterns. **(C)** Class distribution of significantly altered lipid species. Pie charts summarize the proportion of lipid classes among species significantly altered between WT and *Ephb4*-KO groups (Wilcoxon rank-sum test, $P < 0.05$). Percentages represent the fraction of significant lipid species belonging to each class relative to the total number of significantly changed lipids within each genotype. **(D)** Distribution of phospholipid species by total double bond number. **(E)** Volcano plot of differentially regulated lipid species in WT and *Ephb4*-KO MC38 tumor cells. The x-axis represents the $\log_2$ fold change in lipid abundance (WT vs. *Ephb4*-KO), and the y-axis shows $-\log_{10} P$ value derived from Wilcoxon rank-sum testing. Blue and red dots indicate lipid species significantly elevated in WT and *Ephb4*-KO groups, respectively (Wilcoxon rank-sum test, $P < 0.05$). Gray dots represent lipid species that did not reach statistical significance. Selected lipid species with the highest significance are annotated. Abbreviations: Cer, ceramide; DB, double bond; DG, diacylglycerol; Ephb4, ephrin receptor b4; FC, fold change; GlcCer, glucosylceramide; IR, irradiation; KO, knockout; LacCer, lactosylceramide; LPC, lysophosphatidylcholine; MRM, multiple reaction monitoring; PA, phosphatidic acid; PC, phosphatidylcholine; PCA, principal component analysis; PE, phosphatidylethanolamine; PG, phosphatidylglycerol; PI, phosphatidylinositol; PS, phosphatidylserine; SM, sphingomyelin; TG, triacylglycerol; WT, wildtype.

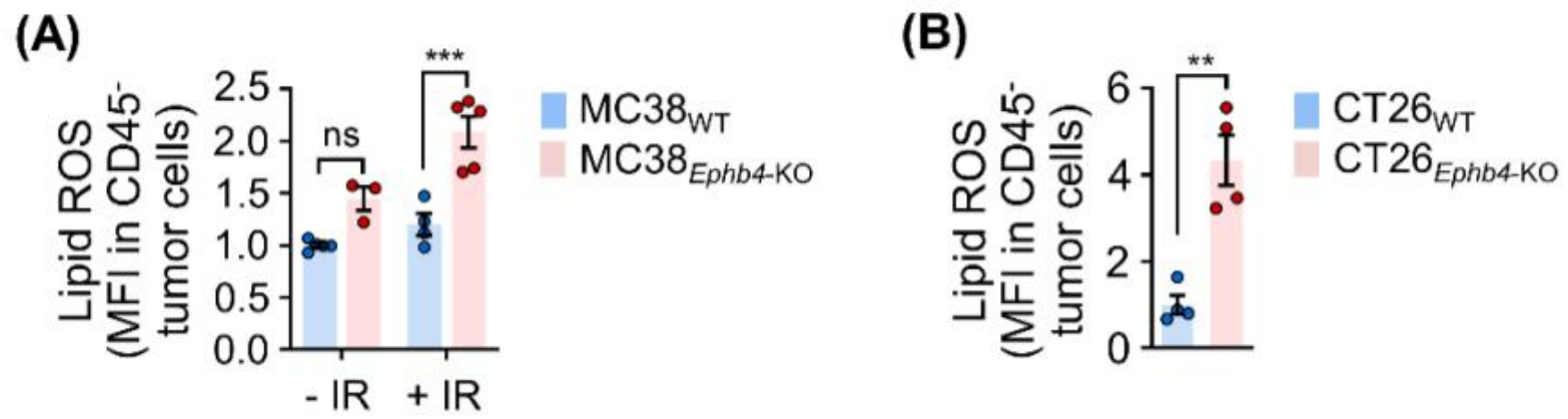

Figure S10. *Ephb4* deletion increased radiation-induced lipid peroxidation in vivo. **(A)** Relative lipid ROS levels in $CD45^{-}$ tumor cell–enriched populations from WT and *Ephb4*-KO MC38 tumors under non-irradiated and irradiated conditions (7 days post-IR, 10 Gy) (n = 3–5 per group). **(B)** Relative lipid ROS levels in $CD45^{-}$ tumor cell–enriched populations from WT and *Ephb4*-KO CT26 tumors 7 days post-IR (10 Gy, n = 4 per group). Data are presented as mean ± SEM (A-B). ns, not significant, $^{**}P < 0.01$, $^{***}P < 0.001$ by two-way ANOVA (A) or unpaired two-tailed Student's t test (B). Abbreviations: Ephb4, ephrin receptor b4; IR, irradiation; KO, knockout; MFI, mean fluorescence intensity; ROS, reactive oxygen species; SEM, standard error of the mean; WT, wildtype.

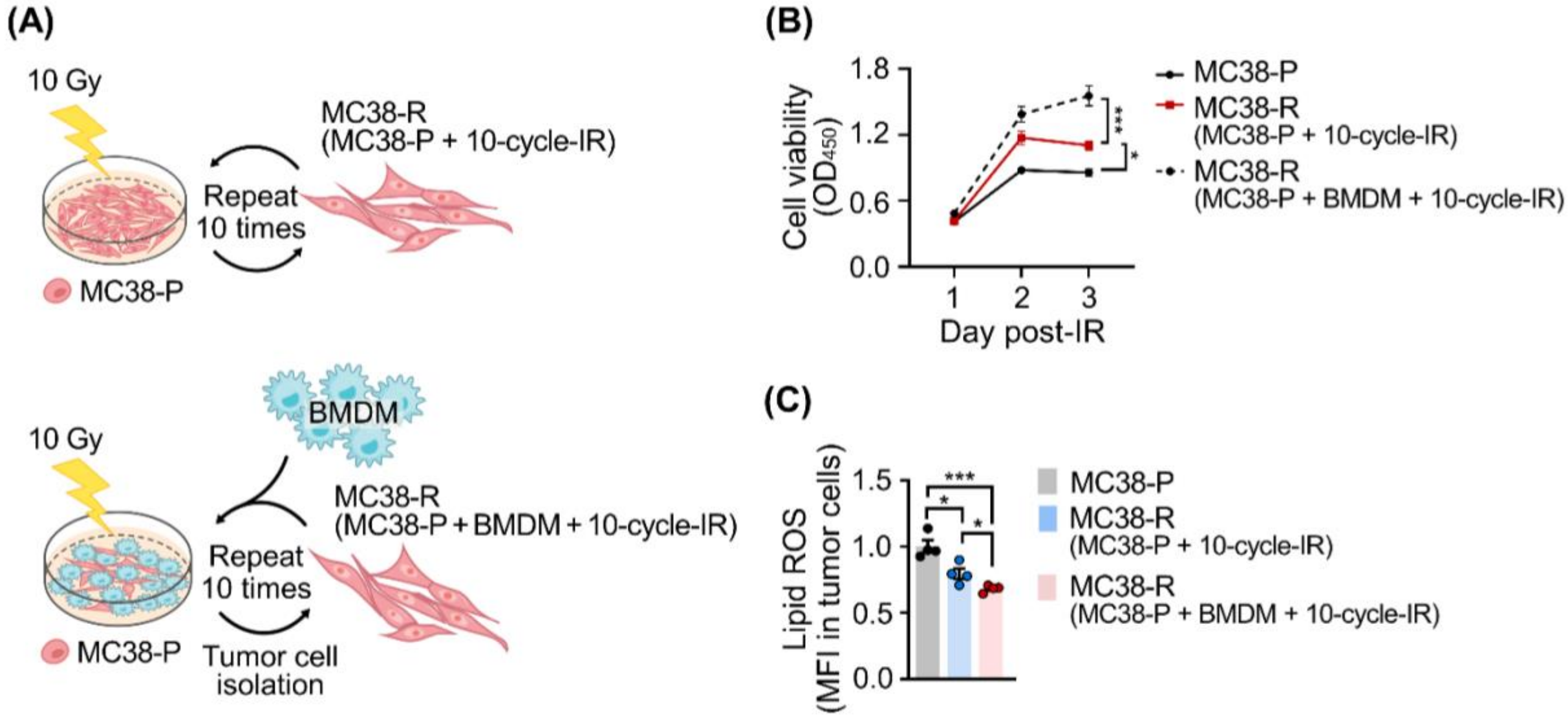

Figure S11. Repeated irradiation exposures were insufficient to induce stable ferroptosis-resistant phenotypes in the absence of macrophages.

(A) Schematic illustration of the experimental design. MC38 parental tumor cells were subjected to repeated cycles of IR (10 Gy, 10 cycles) either alone or in co-culture with BMDMs to generate radioresistant populations (R-tumor cell only and R–co-cultured with BMDMs). **(B)** Cell viability of parental (P), tumor cell-only irradiation–selected (R-tumor cell only), and macrophage co-culture irradiation-selected (R-co-cultured with BMDMs) MC38 tumor cells post-IR (10 Gy), assessed by CCK-8 assay at the indicated time points (*n* = 4 per group). **(C)** Relative lipid ROS levels measured 3 days after 10 Gy IR in P, R-tumor cell only, and R-co-cultured with BMDMs MC38 tumor cells (*n* = 4 per group). Data are presented as mean ± SEM (B-C). *$P < 0.05$, ***$P < 0.001$ by one-way ANOVA with Tukey's multiple comparison test (B-C). Abbreviations: BMDM, bone marrow-derived macrophage; CCK-8, cell counting kit-8; IR, irradiation; MFI, mean fluorescence intensity; P, parental; R, radioresistant; ROS, reactive oxygen species; SEM, standard error of the mean.

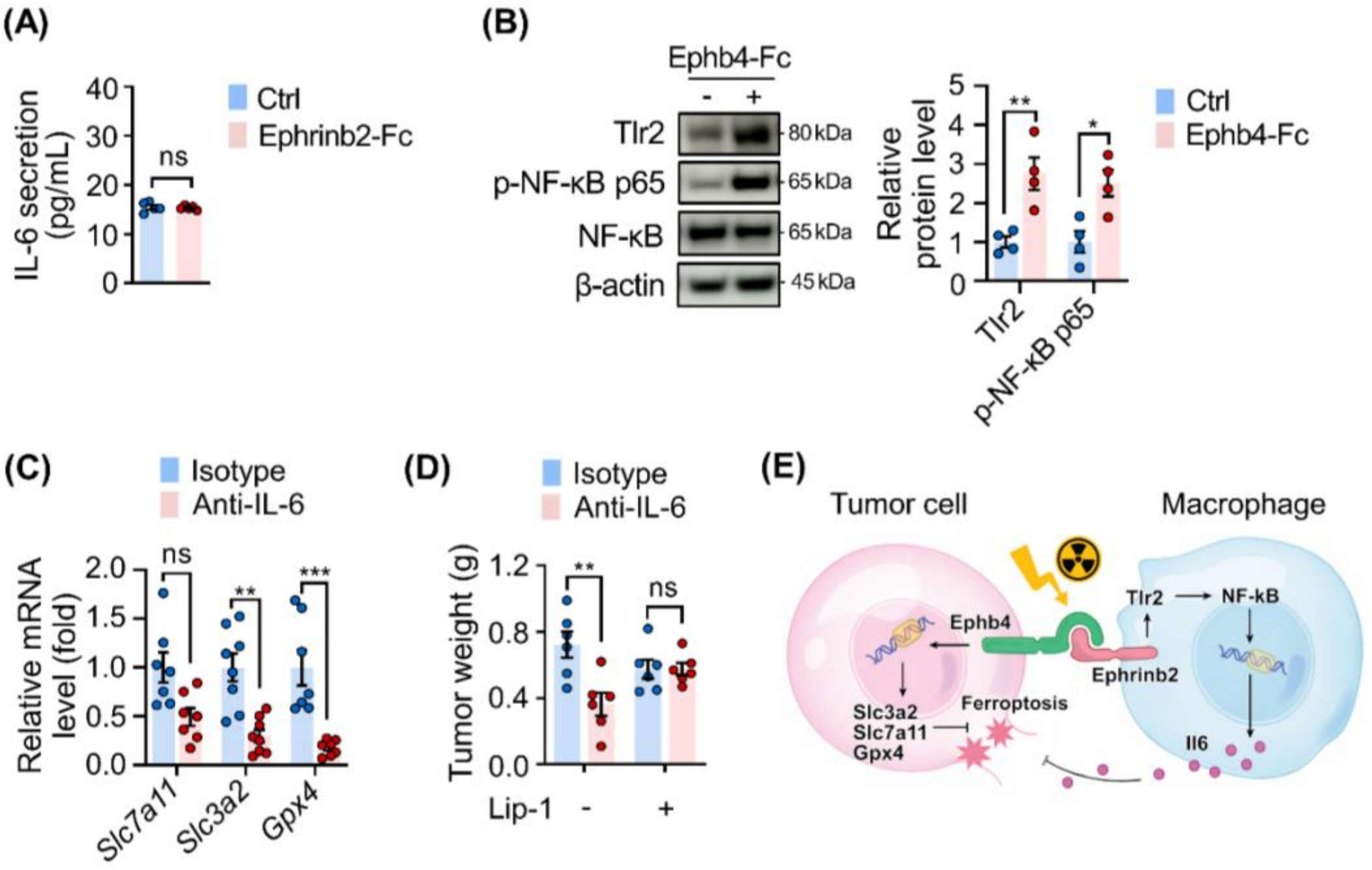

Figure S12. Macrophage ephrinb2–Tlr2–NF-κB–IL-6 signaling mediated protection against radiation-induced ferroptosis in tumor cells.

(A) ELISA quantification of IL-6 in MC38 tumor cells treated with or without recombinant ephrinb2-Fc (10 μg/mL) following IR (n = 5 per group). **(B)** Immunoblotting analysis of Tlr2, p–NF-κB p65, total NF-κB, and β-actin in irradiated BMDMs treated with or without recombinant Ephb4-Fc (10 μg/mL), 24 h post-IR. Right, quantification of Tlr2 protein levels normalized to β-actin and p–NF-κB p65 levels normalized to total NF-κB (n = 4 per group). **(C)** Relative mRNA expression of ferroptosis-suppressive genes (*Slc7a11*, *Slc3a2*, and *Gpx4*) in WT MC38 tumors treated with isotype control or anti-IL-6 after IR (as shown in Figure 6O) (n = 7 per group for *Slc7a11* and *Gpx4*; n = 8 per group for *Slc3a2*). **(D)** Tumor weight at day 14 post-IR (10 Gy) in mice bearing MC38 tumors treated with isotype control or anti-IL-6, with or without ferroptosis inhibitor liproxstatin-1 (Lip-1). Data were pooled from two independent experiments (n = 6 per group). **(E)** Schematic illustration of the bidirectional Ephb4–ephrinb2 signaling axis between tumor cells and macrophages under IR. All irradiation experiments were performed using a single dose of 10 Gy. Data are presented as mean ± SEM (A-D). ns, not significant, *$P < 0.05$, **$P < 0.01$, ***$P < 0.001$ by unpaired two-tailed Student's t test (A) or two-way ANOVA (B-D). Abbreviations: BMDM, bone marrow-derived macrophage; ELISA, enzyme-linked immunosorbent assay; Ephb4, ephrin receptor b4; Gpx4, glutathione peroxidase 4; IL-6, interleukin-6; IR, irradiation; Lip-1, liproxstatin-1; SEM, standard error of

the mean; Slc3a2, solute carrier family 3 member 2; Slc7a11, solute carrier family 7 member 11; Tlr2, toll-like receptor 2; WT, wildtype.

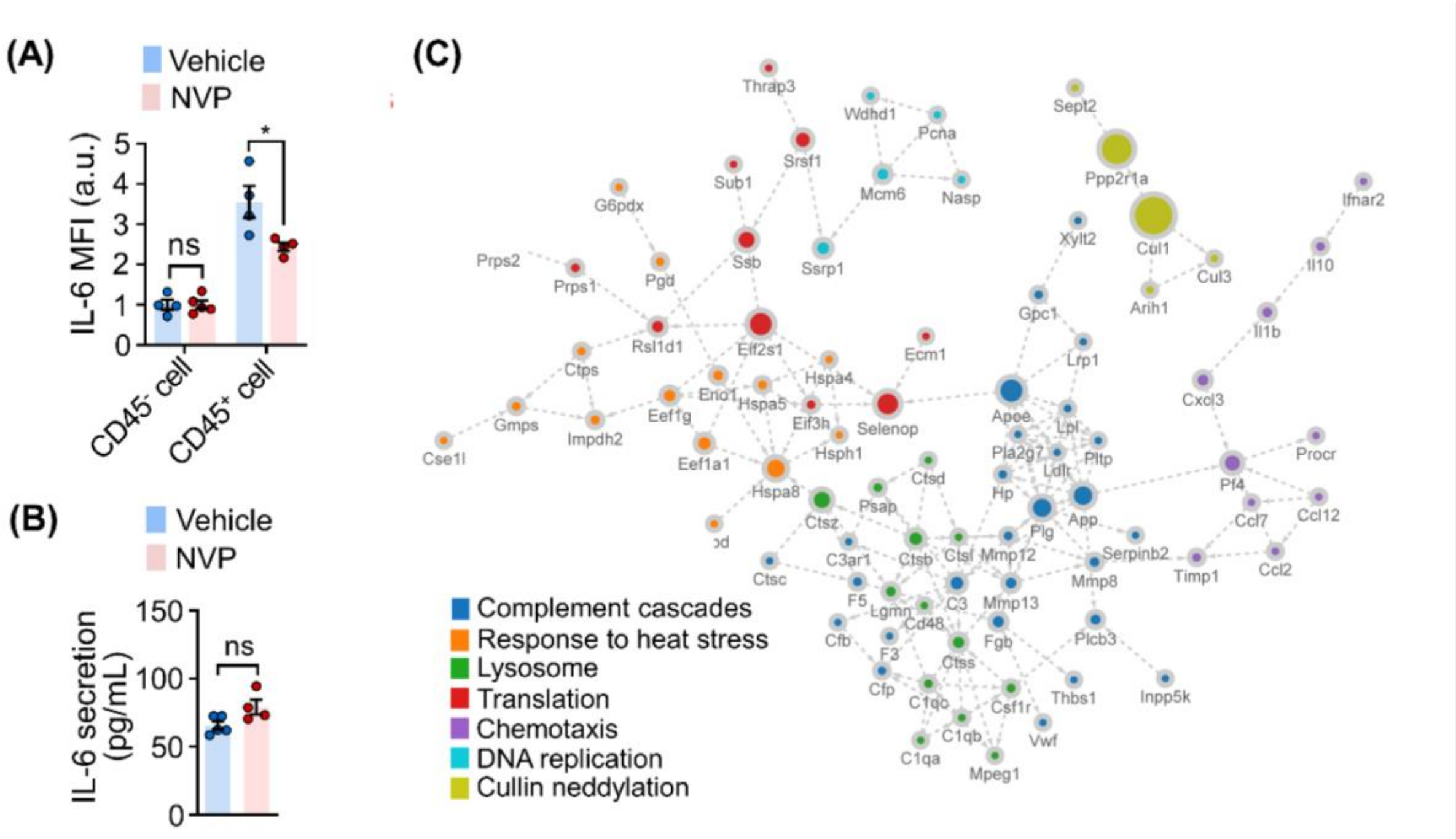

Figure S13. Ephb4 inhibition suppressed macrophage IL-6 and reshaped the tumor secretomes. (A) MFI of IL-6 in $CD45^{-}$ tumor cells and $CD45^{+}$ BMDMs from irradiated MC38 tumor cell–BMDM co-cultures treated with vehicle or NVP (1 μmol/L), as measured by flow cytometry following 10 Gy IR (n = 4–5 per group). **(B)** IL-6 secretion in BMDMs treated with vehicle or NVP, in the absence of tumor cells (*n* = 4–5 per group). **(C)** PPI network of differentially expressed proteins was constructed using Cytoscape based on STRING experimental interaction data, excluding text-mining associations. K-means clustering (k = 7) was applied based on adjusted *P* values to identify functionally distinct protein modules, which were annotated according to representative biological processes. Node size represents betweenness centrality; node color indicates assigned clusters: complement cascades (blue), response to heat stress (orange), lysosomal function (green), translation (red), chemotaxis (purple), DNA replication (cyan), and Cullin neddylation (yellow). Data are presented as mean ± SEM (A-B). ns, not significant, *$P < 0.05$ by two-way ANOVA (A) or unpaired two-tailed Student's t test (B). Abbreviations: a.u., arbitrary units; BMDM, bone marrow-derived macrophage; IL-6, interleukin-6; IR, irradiation; MFI, mean fluorescence intensity; NVP, NVP-BHG712; PPI, protein–protein interaction; SEM, standard error of the mean; STRING, search tool for the retrieval of interacting genes/proteins.

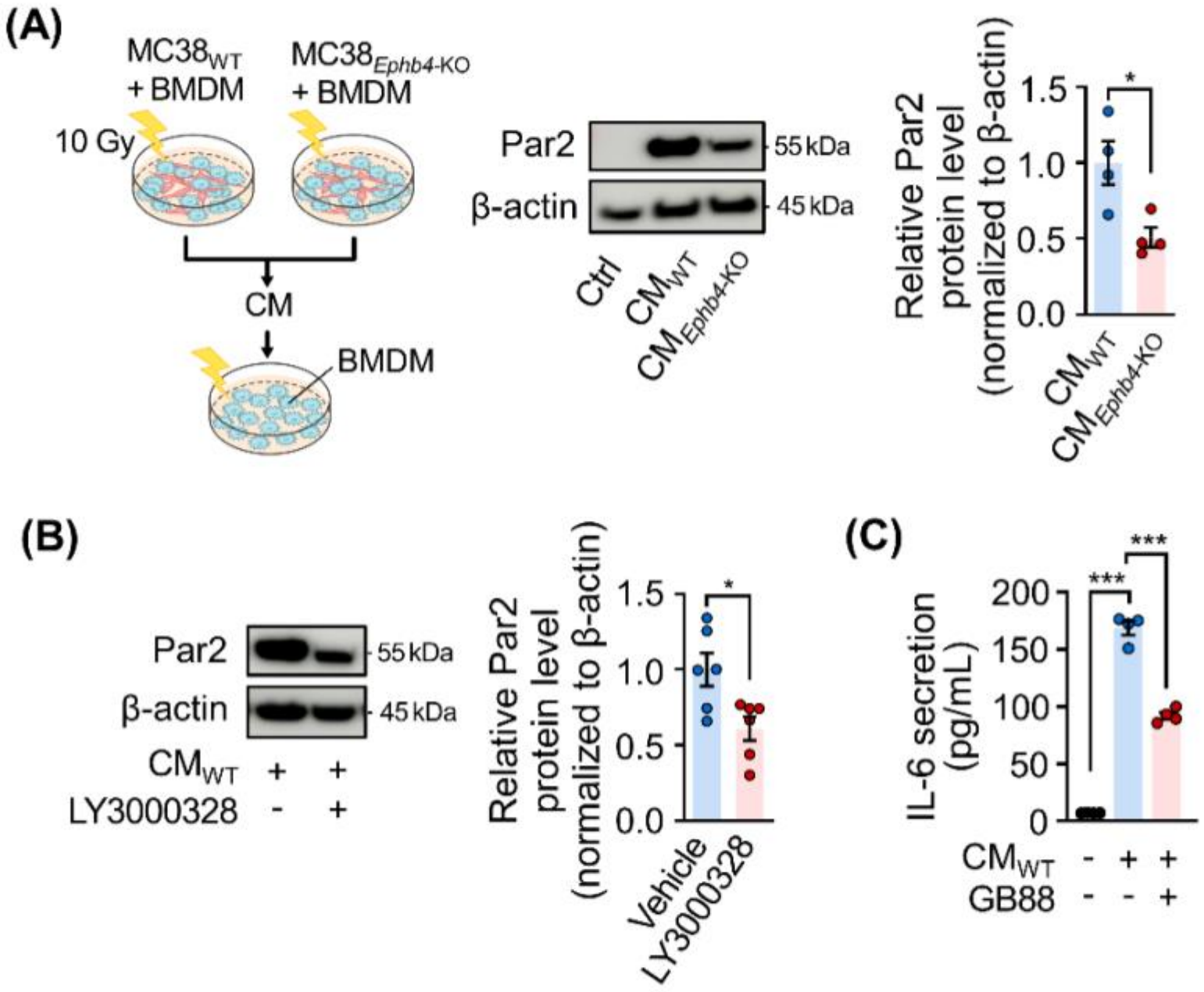

Figure S14. Tumor-derived Ctss engaged macrophage Par2 to induce IL-6 production. **(A)** Schematic illustration of the experimental setup (left). CM collected from irradiated MC38 tumor cell–BMDM co-cultures containing WT (CM_{WT}) or *Ephb4*-KO ($CM_{Ephb4\text{-}KO}$) tumor cells were transferred to irradiated naive BMDMs. Untreated BMDMs (no CM) were used as a control (Ctrl). Par2 expression in BMDMs was analyzed by immunoblotting 24 h after CM treatment (*n* = 4 per group). **(B)** Immunoblotting analysis of Par2 expression in irradiated BMDMs treated with CM_{WT} as shown in (A), in the presence or absence of the Ctss inhibitor LY3000328 (40 μmol/L). Right, quantification of Par2 protein levels normalized to β-actin (*n* = 6 per group). **(C)** ELISA quantification of IL-6 in irradiated BMDMs treated with CM_{WT} as shown in (A), in the presence or absence of the Par2 inhibitor GB88 (10 μmol/L) for 24 h (*n* = 4 per group). All irradiation experiments were performed using a single dose of 10 Gy. Data are presented as mean ± SEM (A-C). *$P < 0.05$, ***$P < 0.001$ by unpaired two-tailed Student's *t* test (A-B), one-way ANOVA with Tukey's multiple comparison test (C). Abbreviations: BMDM, bone marrow-derived macrophage; CM, conditioned medium; Ctrl, control; ELISA, enzyme-linked immunosorbent assay; Ephb4, ephrin receptor b4; IL-6, interleukin-6; KO, knockout; Par2, protease-activated receptor 2; SEM, standard error of the mean; WT, wildtype.

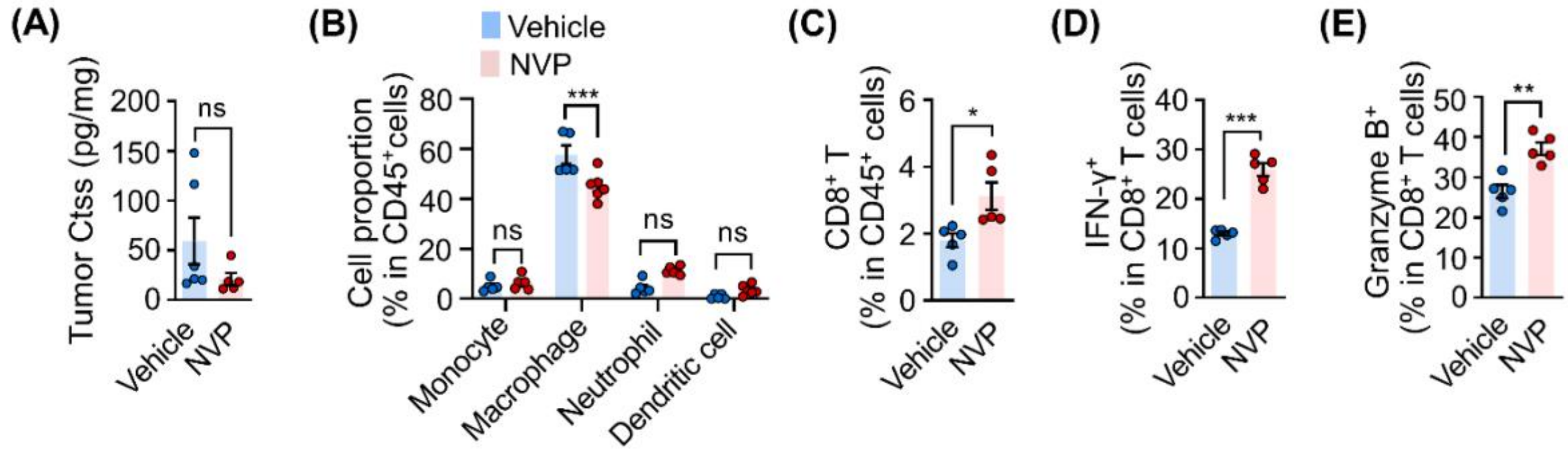

Figure S15. Ephb4 inhibition altered myeloid and lymphoid immune composition in irradiated MC38 tumors.

(A) ELISA quantification of Ctss protein levels in tumor lysates from irradiated MC38 mouse models treated with vehicle or NVP (*n* = 5–6 per group). **(B)** Flow cytometric analysis of tumor-infiltrating myeloid populations in irradiated MC38 tumors treated with vehicle or NVP: monocytes, macrophages, neutrophils, and dendritic cells, shown as a percentage of $CD45^+$ cells (*n* = 4-5 per group). **(C)** Percentage of $CD8^+$ T cells in tumors from vehicle- or NVP-treated mice (*n* = 5 per group). **(D)** Percentage of IFN-$\gamma^+$$CD8^+$ T cells in tumors from vehicle- or NVP-treated mice (*n* = 5 per group). **(E)** Percentage of granzyme $B^+$$CD8^+$ T cells in tumors from vehicle- or NVP-treated mice (*n* = 5 per group). Data are presented as mean ± SEM (A-E). ns, not significant, *$P < 0.05$, **$P < 0.01$, ***$P < 0.001$ by unpaired two-tailed Student's t test (A, C-E) or two-way ANOVA (B). Abbreviations: Ctss, cathepsin S; ELISA, enzyme-linked immunosorbent assay; IFN-γ, interferon-γ; NVP, NVP-BHG712; SEM, standard error of the mean.

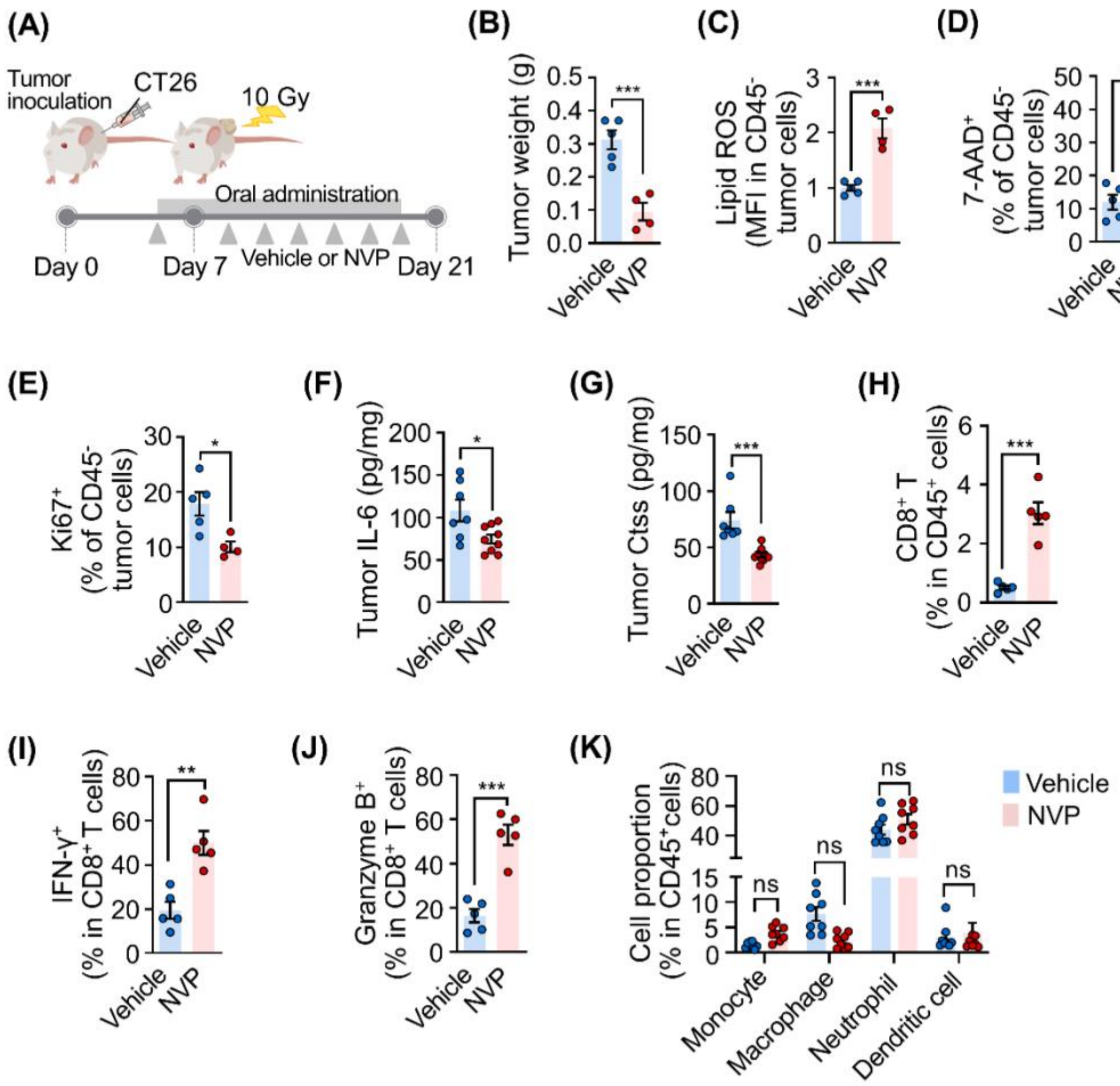

Figure S16. Blocking Ephb4 enhanced ferroptosis and antitumor immunity in irradiated CT26 tumors.

(A) Schematic illustration of the in vivo experimental setup. Mice bearing CT26 tumors were treated with vehicle or NVP (50 mg/kg) by oral gavage starting on day 6 after tumor implantation, followed by 10 Gy irradiation. **(B)** Tumor weight at day 14 post-IR in CT26 tumor–bearing mice treated with vehicle or NVP. **(C)** Relative lipid ROS in $CD45^{-}$ tumor cell–enriched populations from CT26 tumors treated with vehicle or NVP (*n* = 4–5 per group). **(D)** Percentage of 7-AAD^{+} dead cells in $CD45^{-}$ tumor cell–enriched populations from CT26 tumors treated with vehicle or NVP (*n* = 4–5 per group). **(E)** Percentage of $Ki67^{+}$ cells in $CD45^{-}$ tumor cell–enriched populations from CT26 tumors treated with vehicle or NVP (*n* = 4–5 per group). **(F)** ELISA quantification of IL-6 protein levels in tumor lysates from irradiated CT26 mouse models treated with vehicle (*n* = 7) or NVP (*n* = 9). **(G)** ELISA quantification of Ctss protein levels in tumor lysates from irradiated CT26 mouse models treated with vehicle (*n* = 7) or NVP (*n* = 9). **(H)** Percentage of $CD8^{+}$ T cells in tumors from vehicle- or NVP-treated

mice (n = 5 per group). **(I)** Percentage of IFN-γ$^+$CD8$^+$ T cells in tumors from vehicle- or NVP-treated mice (n = 5 per group). **(J)** Percentage of granzyme B$^+$CD8$^+$ T cells in tumors from vehicle- or NVP-treated mice (n = 5 per group). **(K)** Flow cytometric analysis of tumor-infiltrating myeloid populations in irradiated CT26 tumors treated with vehicle or NVP: monocytes, macrophages, neutrophils, and dendritic cells, shown as a percentage of CD45$^+$ cells (n = 8 per group). Data are presented as mean ± SEM (B-K). ns, not significant, *P < 0.05, **P < 0.01, ***P < 0.001 by unpaired two-tailed Student's t test (B-J) or two-way ANOVA (K). Abbreviations: Ctss, cathepsin S; IFN-γ, interferon-γ; IL-6, interleukin-6; MFI, mean fluorescence intensity; NVP, NVP-BHG712; SEM, standard error of the mean; ROS, reactive oxygen species.

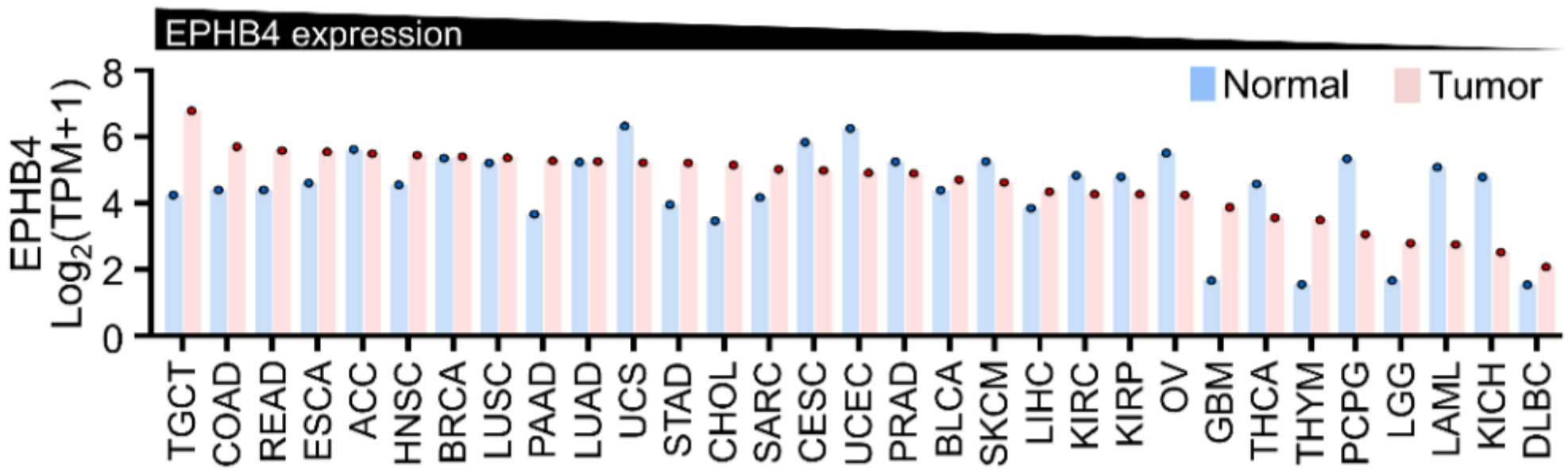

Figure S17. Comparison of *EPHB4* mRNA expression in normal and tumor tissues across various cancer types using TCGA datasets. Expression data were obtained from the GEPIA2 platform and represent median $\log_2$[TPM + 1]. Cancer types are arranged in descending order of *EPHB4* expression in tumor samples, from left to right. Abbreviations: ACC, adrenocortical carcinoma; BLCA, bladder urothelial carcinoma; BRCA, breast invasive carcinoma; CESC, cervical squamous cell carcinoma and endocervical adenocarcinoma; CHOL, cholangiocarcinoma; COAD, colon adenocarcinoma; DLBC, diffuse large B-cell lymphoma; EPHB4, ephrin receptor B4; ESCA, esophageal carcinoma; GBM, glioblastoma multiforme; GEPIA2, gene expression profiling interactive analysis 2; HNSC, head and neck squamous cell carcinoma; KICH, kidney chromophobe; KIRC, kidney renal clear cell carcinoma; KIRP, kidney renal papillary cell carcinoma; LAML, acute myeloid leukemia; LGG, brain lower grade glioma; LIHC, liver hepatocellular carcinoma; LUAD, lung adenocarcinoma; LUSC, lung squamous cell carcinoma; MESO, mesothelioma; OV, ovarian serous cystadenocarcinoma; PAAD, pancreatic adenocarcinoma; PCPG, pheochromocytoma and paraganglioma; PRAD, prostate adenocarcinoma; READ, rectum adenocarcinoma; SARC, sarcoma; SKCM, skin cutaneous melanoma; STAD, stomach adenocarcinoma; TCGA, The Cancer Genome Atlas; TGCT, testicular germ cell tumors; THCA, thyroid carcinoma; THYM, thymoma; TPM, transcripts per million; UCEC, uterine corpus endometrial carcinoma; UCS, uterine carcinosarcoma; UVM, uveal melanoma.

Supplementary Tables

Table S1. List of antibodies used in this study

Antibodies	Manufacturer	Catalog number	Application	Dilution
Ephb4	R&D Systems	AF446	Western blotting	1:1000
Par2	Abcam	AB180953	Western blotting	1:1000
Atf4	Cell Signaling Technology	11815S	Western blotting	1:1000
Tlr2	Invitrogen	MA5-16200	Western blotting	1:1000
Slc7a11	Cell Signaling Technology	12691S	Western blotting	1:1000
Slc3a2	Cell Signaling Technology	47213S	Western blotting	1:1000
Gpx4	Abcam	AB125066	Western blotting	1:1000
phosphor-NF-κB p65	Cell Signaling Technology	3033S	Western blotting	1:1000
NF-κB p65	Cell Signaling Technology	4764S	Western blotting	1:1000
β-actin	Santa Cruz	sc-47778	Western blotting	1:5000
Ki67	Cell Signaling Technology	12202S	Immunofluorescence	1:2000
F4/80	Abcam	AB6640	Immunofluorescence	1:1000
Pan-cytokeratin	Invitrogen	53-9003-82	Immunofluorescence	1:500
CD68	Abcam	AB53444	Immunofluorescence	1:400
Ephrinb2	R&D Systems	AF496	Immunofluorescence	1:100
Ptgs2 (Cox2)	Cell Signaling Technology	12282S	Immunofluorescence	1:1000
DAPI	Invitrogen	62248	Immunofluorescence	1:10000
Ephb4	Invitrogen	37-1800	Immunofluorescence	1:100
EPHB4	Invitrogen	37-1800	Immunohistochemistry	1:100
7-AAD	BioLegend	640934	Flow cytometry	1:300
BioTracker 575 Red Fe^{2+}	Merck	SCT030	Flow cytometry	1:200
CD16/CD32	eBioscience	14-0161-82	Flow cytometry	1:500
CD45 APC-Cy7	eBioscience	47-0451-82	Flow cytometry	1:500
CD11b eFluor 450	eBioscience	48-0112-82	Flow cytometry	1:500
Ly6G FITC	eBioscience	11-9668-82	Flow cytometry	1:500

Ly6C PerCP-Cy5.5	eBioscience	45-5932-82	Flow cytometry	1:500
F4/80 PE	eBioscience	12-4801-82	Flow cytometry	1:300
CD4 PE	eBioscience	12-0041-82	Flow cytometry	1:500
Foxp3 V450	eBioscience	48-5773-82	Flow cytometry	1:300
Granzyme B PE-Cy7	eBioscience	396410	Flow cytometry	1:300
MHC Class II APC	eBioscience	17-5321-82	Flow cytometry	1:500
CD11c BV605	BioLegend	117333	Flow cytometry	1:500
F4/80 FITC	BioLegend	123108	Flow cytometry	1:300
CD3 FITC	BioLegend	100204	Flow cytometry	1:300
CD3 PE-Cy7	BioLegend	100220	Flow cytometry	1:300
CD206 PE	BioLegend	141706	Flow cytometry	1:300
IL-6 APC	BioLegend	504508	Flow cytometry	1:300
Tlr2 BV421	BioLegend	148605	Flow cytometry	1:300
IFN-γ PerCP-Cy5.5	BioLegend	505822	Flow cytometry	1:300
CD45 APC	BD Biosciences	559864	Flow cytometry	1:500
CD8a BV711	BD Biosciences	563046	Flow cytometry	1:300

Abbreviations: DAPI, 4´,6-diamidino-2-phenylindole; Ephb4, ephrin receptor b4; Foxp3, forkhead box P3; Gpx4, glutathione peroxidase 4; IFN-γ, interferon-γ; IL-6, interleukin-6; MHC, major histocompatibility complex; Par2, protease-activated receptor 2; PTGS2, prostaglandin-endoperoxide synthase 2; Slc3a2, solute carrier family 3 member 2; Slc7a11, solute carrier family 7 member 11; Tlr2, toll-like receptor 2.

Table S2. Primer sequences used for gene amplification in this study

Gene (for qRT-PCR)	Primer sequence (5'→3')
Slc7a11	Forward: TGG GTG GAA CTG CTC GTA AT<br>Reverse: AGG ATG TAG CGT CCA AAT GC
Slc3a2	Forward: ACC TCA CTC CCA ACT ACC AG<br>Reverse: ATC AGC TTT CCC ACA TCC C
Gpx4	Forward: GCA ACC AGT TTG GGA GGC AGG AG<br>Reverse: CCT CCA TGG GAC CAT AGC GCT TC
Il6	Forward: TGG AGT CAC AGA AGG AGT GGC TAA G<br>Reverse: TCT GAC CAC AGT GAG GAA TGT CCA C
Tlr2	Forward: TGC TTT CCT GCT GGA GAT TT<br>Reverse: TGT AAC GCA ACA GCT TCA GG
Mrc1	Forward: CTC GTG GAT CTC CGT GAC AC<br>Reverse: GCA AAT GGA GCC GTC TGT GC
Vegfa	Forward: GGA CGT CTA CCA GCG AAG CT<br>Reverse: CAC AGG ACG GCT TGA AGA TGT
Arg1	Forward: CAG AAG AAT GGA AGA GTC AG<br>Reverse: CAG ATA TGC AGG GAG TCA CC
Epha1	Forward: GCC TGG CCC TTT CTC CCC TG<br>Reverse: TCT CTG TCT CTG GCC TCT CC
Epha2	Forward: TGG ATG GCG AGT GGC TGG TG<br>Reverse: TTG GGG GCG AGG GTG GAC G
Epha3	Forward: AAG CAG GAG CAA GAG ACG A<br>Reverse: CAC CGG AGA TGG AGA AAG AG
Epha4	Forward: TCT TTT CGT TTC TCT TTG G<br>Reverse: GTT GTT CTG GCT GGC TTC C
Epha5	Forward: AAG GGC AAA GAA GCG GGA C<br>Reverse: GGA AGG GGC GAG AGA GAC G
Epha6	Forward: ATT CTT CCT CTT TGG TTG<br>Reverse: GGT GGG TCT TTT TCT GCC
Epha7	Forward: TCT GGC TGC TTG GCT TTG C<br>Reverse: TCT GTT TGT TGT GCT TTC G
Epha8	Forward: TCA CCA CGA ACC AGG CAG<br>Reverse: GAG AAG CAA GAG GAG CAC
Ephb1	Forward: TTA CAG CAC AGG CCG AGG GGA GTT CG<br>Reverse: AAC TGG CCC ATG ATG CTC GCC
Ephb2	Forward: ACG CCA CGG CCA TAA AAA GCC C<br>Reverse: TTG CCA CTG TAG CGC CCA TAG C
Ephb3	Forward: ATT GGG CAT CAA GCC ACC CAG C<br>Reverse: TGC TCT GTA ACC GAG GTG TCG C
Ephb4	Forward: TTG AGC CCT GGG TGG CAA TCC G<br>Reverse: AGG CAC CTC ACG GTC AGT GG
Ephb6	Forward: ACT CTA AGC TGC GAG CAG ACG C<br>Reverse: GCC AGG CTT GCC TTC TTG TCT GG
18S rRNA	Forward: GCA ATT ATT CCC CAT GAA CG<br>Reverse: GGC CTC ACT AAA CCA TCC AA

Abbreviations: Arg1, arginase 1; Epha1, ephrin receptor a1; Epha2, ephrin receptor a2; Epha3, ephrin receptor a3; Epha4, ephrin receptor a4; Epha5, ephrin receptor a5; Epha6, ephrin receptor a6; Epha7, ephrin receptor a7; Epha8, ephrin receptor a8; Ephb1, ephrin receptor b1; Ephb2, ephrin receptor b2; Ephb3, ephrin receptor b3; Ephb4, ephrin receptor b4; Ephb6, ephrin receptor b6; Gpx4, glutathione peroxidase 4; Il6, interleukin-6; Mrc1, mannose receptor C-type 1; qRT-PCR, quantitative reverse transcription-polymerase chain reaction; Slc3a2, solute carrier family 3 member 2; Slc7a11, solute carrier family 7 member 11; Tlr2, toll-like receptor 2; Vegfa, vascular endothelial growth factor A; 18S rRNA, 18S ribosomal RNA.

Table S3. Radiosensitizer genes selected via mouse siRNA kinome array analysis.

No.	Gene name	Gene full name	Membrane-expressed gene	RLU[a]	RLU ratio[b]
1	*Ripk1*	Receptor-interacting serine/threonine-protein kinase 1	-	0.490	0.155
2	*Sephs2*	Selenophosphate synthetase 2	-	0.562	0.502
3	*Tssk1*	Testis-specific serine kinase 1	-	0.618	0.524
4	*Cask*	Calcium/calmodulin-dependent serine protein kinase 3 membrane-associated guanylate kinase 2	Yes	0.318	0.300
5	*Efna3*	Ephrin A3	Yes	0.471	0.337
6	*Ephb2*	Ephrin type-B receptor 2	Yes	0.674	0.392
7	*Erbb4*	Erb-B2 receptor tyrosine kinase 4	Yes	0.479	0.232
8	*Gk2*	Glycerol kinase 2	-	0.679	0.547
9	*Gsg2*	Serine/threonine-protein kinase haspin (histone H3-associated protein kinase)	-	0.427	0.358
10	*Ikbkb*	Inhibitor of nuclear factor kappa B kinase subunit beta	-	0.395	0.163
11	*Itk*	IL-2-inducible T-cell kinase	-	0.423	0.246
12	*Jak3*	Janus kinase 3	-	0.343	0.241
13	*Lats1*	Large tumor suppressor kinase 1	-	0.286	0.134
14	*Sik1*	Salt-inducible kinase 1	Yes	0.544	0.429
15	*Pkm*	Pyruvate kinase M1/2	-	0.451	0.311
16	*Plk1*	Polo-like kinase 1	-	0.628	0.233
17	*Atr*	ATR serine/threonine kinase	Yes	0.588	0.097
18	*Cpne3*	Copine 3	-	0.494	0.301
19	*Plk2*	Polo like kinase 2	-	0.617	0.392

[a]RLU: luminescence intensity normalized to the negative control well on the same plate;
[b]RLU ratio: ratio of the RLU values of IR-positive/IR-negative groups of the same gene.
Genes with RLU under 0.7 and RLU ratio under 0.6 were selected as radiosensitizer genes.
Abbreviations: IR, irradiation; RLU, relative luminescence unit.
